# On the origin and structure of haplotype blocks

**DOI:** 10.1111/mec.16793

**Published:** 2022-12-15

**Authors:** Daria Shipilina, Arka Pal, Sean Stankowski, Yingguang Frank Chan, Nicholas H. Barton

**Affiliations:** ^1^ Evolutionary Biology Program, Department of Ecology and Genetics (IEG) Uppsala University Uppsala Sweden; ^2^ Institute of Science and Technology Austria Klosterneuburg Austria; ^3^ Swedish Collegium for Advanced Study Uppsala Sweden; ^4^ Friedrich Miescher Laboratory of the Max Planck Society Tübingen Germany

**Keywords:** ancestral recombination graph, coalescent, haplotype block, haplotype‐based methods

## Abstract

The term “haplotype block” is commonly used in the developing field of haplotype‐based inference methods. We argue that the term should be defined based on the structure of the Ancestral Recombination Graph (ARG), which contains complete information on the ancestry of a sample. We use simulated examples to demonstrate key features of the relationship between haplotype blocks and ancestral structure, emphasizing the stochasticity of the processes that generate them. Even the simplest cases of neutrality or of a “hard” selective sweep produce a rich structure, often missed by commonly used statistics. We highlight a number of novel methods for inferring haplotype structure, based on the full ARG, or on a sequence of trees, and illustrate how they can be used to define haplotype blocks using an empirical data set. While the advent of new, computationally efficient methods makes it possible to apply these concepts broadly, they (and additional new methods) could benefit from adding features to explore haplotype blocks, as we define them. Understanding and applying the concept of the haplotype block will be essential to fully exploit long and linked‐read sequencing technologies.

## 1 INTRODUCTION

One of the breakthroughs of long and linked‐read sequencing technologies is the emergence of new methods for obtaining reliable haplotype information for large data sets (Meier et al., 2021). Although most studies of genome‐wide variation still focus on single nucleotide polymorphism (SNP) data, we are approaching the stage where population‐scale haplotype information will be widely available for organisms across the tree of life. In light of this shift from site‐based to haplotype‐based inference, this article considers one of the fundamental concepts for haplotype‐based inference—the definition of the haplotype block.

“Haplotype” and “haplotype block” are widely used terms in evolutionary genetics, and have increased in importance across many disciplines (Delaneau et al., 2019; International HapMap Consortium, 2005; Leitwein et al., 2020). An important but often overlooked fact is that populations evolve through changing frequencies of blocks of the genome, not individual sites. Therefore, we should be most interested in understanding the trajectories of the underlying haplotypes, yet these are often obscured at the level of SNPs (Castro et al., 2019; Clark, 2004). Thus, disentangling the evolutionary history underlying genomic patterns can be challenging using solely site‐based statistics. For example, while whole‐genome scans for signatures of selection can reveal individual SNPs associated with fitness differences (Poelstra et al., 2014; Tavares et al., 2018), it is extremely difficult to pinpoint the causal variants (Burri, 2017; Grossman et al., 2010; Ravinet et al., 2017; Rockman, 2012; Stankowski et al., 2019; Tavares et al., 2018; Wolf & Ellegren, 2017). As another example, shifts in polygenic scores from genome‐wide association studies (GWAS) can be misinterpreted as signals of selection, as opposed to artefacts of population structure (Berg et al., 2019; Novembre & Barton, 2018; Sella & Barton, 2019), which often leave clearer signatures in shared haplotype structure. Similarly, methods for estimating population density and gene flow struggle to distinguish among a virtually infinite number of possible population structures, made worse by assuming independence between SNPs, rather than haplotypes (Richardson et al., 2016; Sousa et al., 2011; Whitlock & Mccauley, 1999).

By accounting for haplotype structure, it should be possible to make inferences more accurate and more efficient. Haplotypes carry information not only from *mutation* but also from *recombination*, which provides an additional “clock” that can help reveal past events (e.g., Ralph & Coop, 2013). Primarily for these reasons, there has been a steady increase in analytical methods that aim to infer haplotype structure from sequence data, or that exploit haplotype structure to make inferences about selection, gene flow and population structure.

Although there has been significant progress towards the broader use of haplotype information in empirical studies (see overview in Box 1), much of this lacks a unifying concept across fields spanning evolutionary and conservation genetics (Leitwein et al., 2020), human and medical genetics (Crawford & Nickerson, 2005), and animal and plant breeding (Bhat et al., 2021; Mészáros et al., 2021). There is thus often little consensus on how haplotype blocks are defined, which complicates comparison of results. Worse, it may preclude insights that may otherwise arise from spotting commonalities that emerge under vastly different population parameters.

BOX 1 **Ancestral recombination graph (ARG)**

**FIGURE A1:**
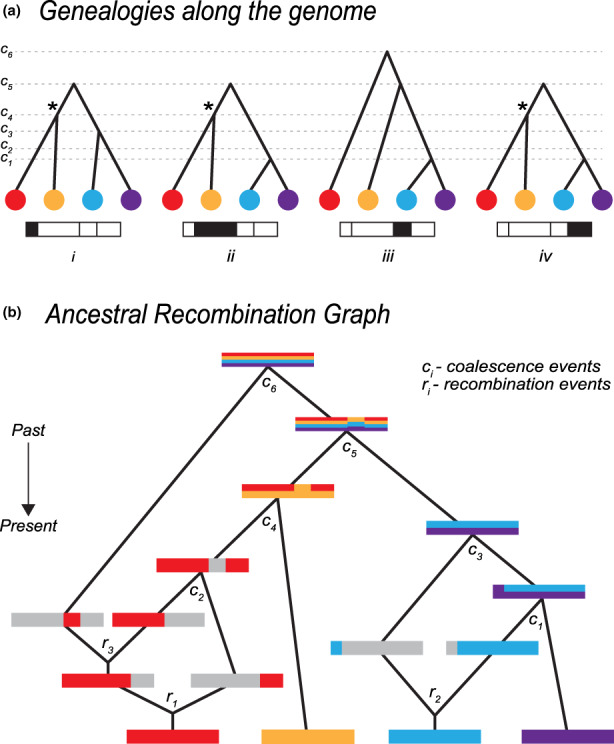
Relationship between genealogies and the ancestral recombination graph (ARG). (a) Genealogical trees along the genome, corresponding to the ARG—Each tree describes the ancestral relationship for each of the four nonrecombined regions. *c*_1_, *c*_2_, …, *c*_6_ denote time points for each coalescence event. Trees can either change, have the same topology or marginally differ by only temporal positions of coalescence nodes. An asterisk (*) denotes a unique coalescence event that is ancestral to disjunct genomic regions. (b) Full representation of the ARG. Tracing back ancestry of four genomes, there is either recombination splitting lineages or coalescence merging lineages. Inherited ancestral genomic regions are coloured corresponding to the contemporary genomes. Recombination is represented by splitting the genome into two, where grey denotes a nonancestral genomic region. Coalescence is represented by two genomes merging, with inherited genomic regions denoted by mixed colours. There are three recombination and six coalescence events in the full ancestral history of the four genomes. *c*_1_, *c*_2_, …, *c*_6_ denote time points for each coalescence event. *r*_1_, *r*_2_ and *r*_3_ denote time points for each recombination event

The ARG describes the complete ancestry of a sample of genomes through a series of real coalescence and recombination events (Griffiths & Marjoram, 1997; Hudson, 1983). At any given site on the genome, the relationship can be described through a genealogy (Kingman, 1982); all contemporary samples coalesce and eventually trace back to one single ancestor. Moving along the genome, the relationship inevitably changes due to recombination. This leads to a series of observable genealogies along the genome (Figure A1a), which are embedded in a single structure: the ARG (Figure A1b).

The full ARG (Figure A1b) is a graph structure that depicts individuals' (both ancestral and extant) lineage relationships in time. Each node in the ARG represents a real coalescence or recombination event, whilst edges represent the ancestry of a particular genomic segment, along a genetic lineage (depicted by coloured/grey segment for inherited/noninherited genetic material in Figure A1b). Altogether, an ARG describes the entire ancestral history—each recombination and each coalescence event, which imply the genealogy for each nonrecombined genomic block. Crucially, the ARG describes ancestry but not allelic state, so is independent of all the mutations that lead to the observed polymorphism in the present sample.

It is important to note that the full ARG (Figure A1b) contains more information than the series of tree sequences along the genome (Figure A1a). First, a series of tree sequences lack information on the timing of recombination events, unless these are separately stored. Second, while some recombination events lead to observable changes in genealogical trees, others might not. Figure A1a depicts such cases—some recombination events might not change the tree topologies at all (trees *ii* and *iv* are exactly the same), whereas others might only lead to temporal changes in coalescence nodes (tree *i* differs from trees *ii* and *iv* by one node position, but all have the same topology). Therefore, while there are four nonrecombining genomic regions, there are only two unique tree topologies (trees *i*, *ii* and *iv* have the same topology) and three distinct trees (trees *ii* and *iv* are exactly the same). Some coalescence events can also be entirely invisible and not be represented in any of the individual trees—coalescence at *t*_2_ in Figure A1b is not represented in the series of trees in Figure A1a. Furthermore, two disjunct blocks of the genome can be inherited from the same ancestor, so that a unique coalescence event (e.g., marked by an asterisk in Figure A1a) can generate disjunct blocks of ancestry. It should also be noted that although Figure A1 shows the inevitable coalescence of the whole genome into a single common ancestor, this typically takes an astronomically long time: each nonrecombining region of the genome coalesces at various time points, and the single lineages ancestral to each region then take an extremely long time to coalesce in one common ancestor, in a process which is in principle unobservable.

Since the ARG contains full information about the genealogy of the sample, it is in theory sufficient to infer any evolutionary process: the ARG necessarily gives more information than commonly used statistics such as site frequency spectrum (SFS), *F*_ST_ and extended haplotypes homozygosity (EHH), which are low‐dimensional summaries of the ARG (Ralph et al., 2020). Therefore, the ARG should serve as the foundation for developing new methodologies. However, we note that whilst the ARG is a sufficient statistic, it remains an open question how much the extra information it gives can improve inference: the intrinsic variability of the evolutionary process sets a bound on the accuracy of our inferences.

Motivated by the arrival of powerful new data sets and analysis methods, the main goal of this paper is to examine the fundamental definition of the haplotype block. We propose a definition of haplotype block based on the full genealogy, represented by the Ancestral Recombination Graph (ARG). Using simulations of simple but general scenarios, we explore how the characteristics of haplotype blocks relate to the origin of the samples and segregating SNP variation. We then discuss how the proposed definition relates to practical inference methods and their applications in large‐scale population studies. We consider how different methods make use of haplotype information and infer haplotype blocks, their underlying assumptions and respective limitations.

## 2 DEFINING HAPLOTYPE BLOCKS

A haplotype has a clear definition: it is simply a haploid genotype (e.g., the genotype of the sperm or egg). In contrast, the term “haplotype block” is used widely, but in many different ways (Al Bkhetan et al., 2019; Clark, 2004; International HapMap Consortium, 2005; Schwartz et al., 2003; Taliun et al., 2014; Zhang et al., 2002). Since haplotype structure arises through segregation and recombination, our understanding of “haplotype blocks” must depend on the processes of coalescence and recombination that generate it in the first place. With this in mind, we contrast alternative definitions, and settle on one, which is based on branches in the underlying genealogy.

**FIGURE 1:**
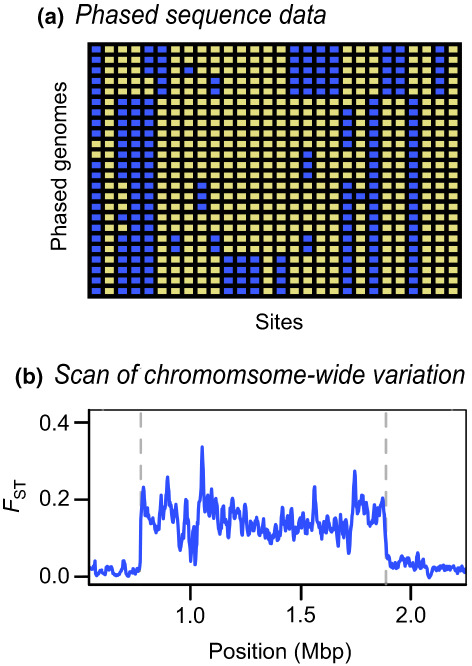
Block‐like patterns in empirical data. (a) Block‐like patterns in phased DNA sequences from *Mimulus auranticus* within the gene *MaMyb2* (Stankowski & Streisfeld, 2015). Rows show 24 individual haplotypes. Each column is a site, with yellow and blue squares representing ancestral and derived sites, respectively. (b) an *F*_ST_ scan across *Heliconius* chromosome 2 reveals a large plateau of differentiation on chromosome 2 between races of *H. erato* (Meier et al., 2021). This large block‐like pattern coincides with a chromosomal inversion, the boundaries of which are illustrated by the dashed line

In sequence data, we usually observe the diploid genotypes; resolving them into the two haploid genotypes is termed “phasing.” With *n* heterozygous sites, there are 2^*n*^ possible pairs of haplotypes—more than a million with just *n* = 20. However, in real populations there are usually far fewer haplotypes, due to linkage disequilibrium (LD) across polymorphic sites, which produces strong haplotype structure. This allows “statistical phasing,” through which one reconciles diploid genotypes into the underlying haplotype pair (Browning & Browning, 2011). Looking across individuals in larger genotype panels, the more frequent haplotypes often appear as stretches of shared, “banded” blocks of SNPs (Figure 1a). This can be especially striking when different haplotypes become fixed across populations, which can produce block‐like patterns in data even when individual haplotypes cannot be observed (Figure 1b); in some cases, they have been referred to as “haploblocks” (Todesco et al., 2020).

Whilst a block‐like structure may be apparent within empirical genetic data, we argue here that there should be a more fundamental definition of haplotype block, based on the true ancestry of the sequences, independent of the mutations that generated observable SNPs. Thus, we separate the *definition* of haplotype blocks from the *estimation* of these blocks from actual data.

**FIGURE 2:**
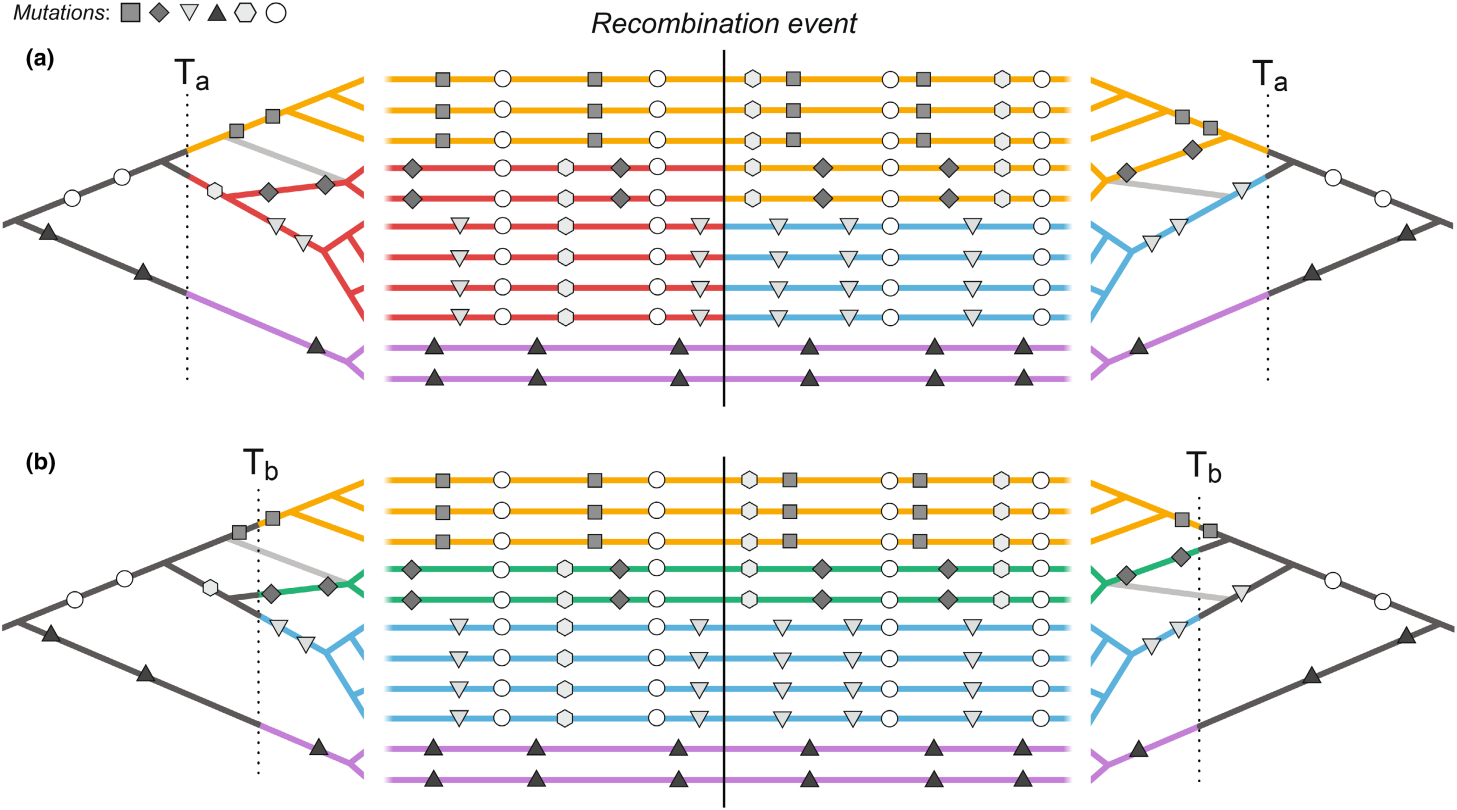
Haplotype blocks defined through identity by descent (IBD). Panels (a) and (b) show the same 11 hypothetical DNA sequences depicted as horizontal lines. The trees on the left and right sides show the genealogy for the set of sequences on either side of a recombination event (indicated by the vertical black line); the light grey branch in both trees shows how the effect of recombination changes the structure of the genealogy on either side. Mutations are shown as symbols that correspond to the branches upon which they arose. Under the IBD definition, haplotype blocks can be defined based on DNA segments that derive from a given set of ancestors, shown here by the coloured sections of branch and DNA sequence. The only difference between panels (a) and (b) is that these ancestors are defined at two different arbitrary time points, T_a_ and T_b_, yielding different haplotype structure

There have been previous attempts at defining haplotype blocks via the classical concept of identity by descent (Carmi et al., 2013; Hartl et al., 1997; Thompson, 2013). Imagine an initial population, where each founder genome is labelled by a different colour. At some later time, each region of the genome must derive from one or other founder, and so will appear as a mosaic of blocks of different colours, each corresponding to their ancestors. This naturally defines blocks that descend from a given set of founders (Figure 2). Fisher (1954) showed that the junctions between identity by descent blocks segregate like Mendelian variants, and used this idea to understand the distribution of runs of homozygosity.

In artificial populations, we can now sequence the founders, and thus directly observe blocks defined in this way (Lundberg et al., 2017; Otte & Schlötterer, 2021; Wallberg et al., 2017). Moreover, if we disregard new mutations, the evolutionary processes subsequent to the founding of the population are entirely described by the block structure. Identity by descent is usually defined with respect to a specific ancestral reference population. However, for natural populations, there is no obvious reference population, so the block structure will vary depending on our arbitrary choice of founders at an arbitrary time point (Figure 2); this complicates the common practice of representing contemporary samples by admixture between well‐mixed founder populations (e.g., structure; Pritchard et al. 2000).

To eliminate this subjectivity, we will base our definition of “haplotype block” on the full ancestry of the sampled genomes, namely on the ARG (Hudson, 1983). The ARG consists of the segments of past genomes that are ancestral to our sample; looking back in time, it is generated by a series of coalescence events that join lineages and of recombination events that split lineages (Box 2). We emphasize that these are real events: coalescence occurs when an actual individual leaves two or more offspring that are each ancestral to our sample, and recombination occurs between the two haploid parent genomes during meiosis in an ancestral individual. Together, these processes define the ARG (Figure A1).

BOX 2 **Population genetic methods that make use of haplotype information**

Many methods for inferring evolutionary processes make use of haplotype structure. These can be roughly grouped into three types based on their underlying paradigm: window‐based methods, segment‐based methods and tree‐based methods. These methods vary in complexity from simple heuristics to full statistical treatments. Here we discuss window‐ and segment‐based methods, but we reserve our discussion of tree‐based methods to the main text.

Of the three classes, window‐based methods tend to be the simplest, and primarily operate *across* sets of individuals. In the simplest form, haplotypes are operationally defined as the set of alleles observed at the segregating sites within a predefined window of an arbitrary length, say, 50 SNPs or 100 kb. Ideally, window sizes should be short enough to minimize spanning recombination breakpoints. One example is H_12_, which detects selective sweeps (Garud et al., 2015). In this test, for any given window, haplotypes are rank‐ordered by their frequencies; in the case of a selective sweep at a given locus, we expect the two most common haplotypes (H_1_ and H_2_) to dominate the population. The H_12_ test features enhanced power to detect selection, especially under competing sweeps between recurring mutations. However, the test does not attempt to capture the real haplotype block length and is rather heuristic. Other fixed window‐based applications include ones exploiting local genomic structures, especially ones showing geographical structure or associated with local adaptation—data‐driven clustering/DDC in Jones et al. (2012); see also Li and Ralph (2019); Todesco et al. (2020). While window‐based methods do not explicitly infer or use information of haplotype block length, they sometimes do take the genealogical structure into account, such as twisst (Lohse et al., 2016; Martin & Van Belleghem, 2017). Often, the simplicity of window‐based methods is also their main appeal in the era of SNP genotyping.

Segment‐based methods are more sophisticated. They operate primarily on individual sequences, with the aim to represent haplotypes as a mosaic of segments from a haplotype panel, often under some version of the Li and Stephens algorithm. These segments offer a more realistic model of recombination breakpoints and confer superior power to capture signatures due to linkage. EHH (Sabeti et al., 2002) is an excellent example of such segment‐based statistics for inferring selection. Along with its derivatives, such as integrated haplotype score (iHS) (Szpiech & Hernandez, 2014; Voight et al., 2006) and cross‐population EHH (XP‐EHH) (Sabeti et al., 2007), they have been widely used to detect selection in many systems (Cao et al., 2011; International HapMap Consortium, 2005). These methods typically seek to capture the decay of a signal, say, in the extent of haplotype sharing, from an a priori defined core SNP. More sophisticated methods based on hidden Markov models to infer the haplotype structure are especially helpful in uncovering admixture and introgression (e.g., finestructure; Lawson et al., 2012). This allows for the visualization of the haplotype‐specific ancestry and improved fine‐scale analysis of population structure that is not obvious from unlinked markers.

In large populations, and over long timescales, the ARG is approximated by the coalescent with recombination; in the simplest case, the rate of coalescence is the inverse of the effective (haploid) population size, and the rate of recombination is just the rate of crossover (Griffiths & Marjoram, 1997; Hudson, 1990). Importantly, the coalescent does not describe the entire genealogical relationship of the entire population. Rather, it only summarizes how the subset of sampled individuals are related to each other. Spatial and genetic structure can also be included: ancestral lineages carry a particular set of selected alleles (i.e., a particular genetic background), and are at a particular spatial location. Tracing back in time, lineages move between backgrounds by recombination, and between locations by migration.

**FIGURE 3:**
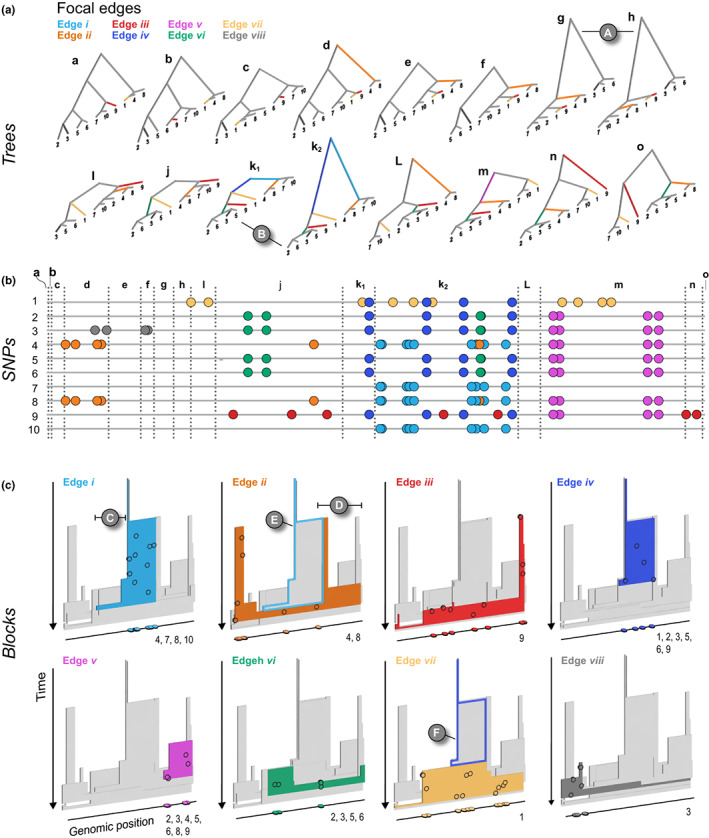
The relationship between trees (a), SNPs (b) and haplotype blocks (c) in the neutral simulation (see main text for simulation details). The ARG has been decomposed into marginal trees (a–o) to show all of the unique topologies that coincide with the genomic spans shown in the central panel (also labelled a–o). The branches for each tree are coloured according to the eight edges in the ARG that we chose to focus on (also labelled *i–vii*). A: two neighbouring topologies that differ only slightly due to recombination. B: an example of two trees (k_1_ and k_2_) that have the same topologies but different lengths. The central panel shows 10 haploid genomes (labelled 1–10, top to bottom, coinciding with the tips of the trees). The SNPs that arose on the eight focal edges are indicated by the coloured circles. The lower panel (c) shows the haplotype blocks for each edge. The coloured block in each panel is the focal edge, with the other seven blocks shown in grey. The mutations shown in the central panel are projected onto each block (black circles) at the genomic location and time that they arose. They are also plotted onto the genomic position axis to make the connection with the panel b more explicit. Similarly, the numbers at the bottom right corner indicate which DNA sequences the mutations are associated with. C and D: Examples of regions of blocks that, by chance, are not revealed by mutations arising on the corresponding edge. E and F: Examples of nested haplotype blocks, where the ancestral block is highlighted with a coloured outline

Informed by the ARG, we could define a haplotype block as a contiguous region of the genome in which all sites share the same genealogy. That is, we could decompose the ARG into marginal trees, each spanning a short region of the genome. However, adjacent genealogies differ by a single recombination event, and so blocks defined in this way will be vanishingly small (especially with large samples) and will usually differ trivially (see A in Figure 3, and Figure A1A). Moreover, as samples get larger, blocks defined in this way will become so small as to be impractical.

Instead, we define a haplotype block as the set of genomic regions that descend from a particular edge in the ARG which is defined by a unique coalescence event, and by the set of descendant samples. Before elaborating on this definition, we clarify our terminology (see Table 1: Glossary). Consistent with the literature, we continue to use “haplotype block” to refer to a region of the genome with a shared pattern of ancestry, without forcing a precise definition. By “branch,” we refer to a lineage on a genealogical tree that connects two coalescence events, or a sampled gene with a coalescence. By “edge,” we refer to the extension of a branch along the genome. Thus, a branch is one‐dimensional, with length measured in generations, whilst an edge is two‐dimensional, with dimensions measured in generations as we trace back through time, and in Morgans as we trace along the genome. An edge is associated with a specific coalescence event, and also with a specific set of descendant samples.

**TABLE 1:** A glossary of key terms

Term	Definition
Ancestral recombination graph (ARG)	A graphical representation of the complete ancestry of a sample of genomes through a series of coalescence and recombination events. The ARG can be decomposed into a series of marginal trees that give the relationships between samples within each nonrecombining region
Branch	A part of a genealogical tree at a single locus, which connects two coalescence events
Coalescence	The merging of lineages in a common ancestor, as one traces lineages backward in time
Edge	A set of genomic regions that are the immediate ancestors of a specific coalescence event, and that are ancestral to a specific set of sampled genomes. An edge has two dimensions (generations × map length). Any SNP that falls on an edge will be shared by the set of descendant genomes, and only by those genomes
Haplotype	A haploid genotype. A diploid genotype consists of a pair of haplotypes
Haplotype block	The set of genomic regions that descend from a particular edge in the ARG, which is defined by a unique coalescence event, and by the set of descendant samples
Identity by descent (IBD)	Segments of the genome are identical by descent if they descend from the same common ancestor
Lineage	A chain of genes that descends from parent to offspring, or (tracing backwards) from offspring to parent
Linkage disequilibrium (LD)	Nonrandom association of alleles at different loci
Phasing	The process of assigning alleles to the maternal and paternal chromosomes in a diploid individual
Time to most recent common ancestor (TMRCA)	The time of the most recent coalescence event from which a focal set of samples descends

This definition means that haplotype blocks exist completely independent of SNPs that may happen to arise on a given edge. However, if mutations have occurred, haplotype blocks will be associated with the set of derived SNP alleles that arise on the focal edge that just precedes the coalescence event. In other words, the set of haplotypes descending from this edge are distinct from all other sampled haplotypes in that they—and only they—share the set of SNPs occurring in the common stem lineage. If enough SNPs happen to arise on an edge, the haplotype block is revealed directly by these shared SNPs.

## 3 IMPLICATIONS OF THE DEFINITION

We next elaborate on the definition and illustrate the relationships between genealogies, SNPs and haplotype blocks using example simulations (a neutral scenario and a selective sweep; Appendix S1 and accompanying GitHub repository: https://github.com/DaSh‐bash/Suppl_Materials_On_the_origin_2022). The simulation uses the standard coalescent (Wakeley, 2009) to generate the ARG, thereby tracking the ancestors of a sample of genomes back through time, until all ancestral genomes are ancestors to the whole sample. It assumes a Wright–Fisher model with a constant population size 2 *N* haploid genomes. A region of the genome of map length *R* is followed, with the selected locus at the leftmost point (i.e., at 0). For simplicity, we allow at most one crossover per generation, with probability *R*; we simulate *R* << 1, so this is close to the case with no interference between crossovers. The simulation can be conditioned on a selective sweep, which is defined by the numbers of copies of the favourable allele in the population. Once the ARG is constructed, genealogies along the genome can be followed, and edges can be identified. Neutral SNPs can be added, assuming infinite‐sites mutation; each SNP is associated with an edge in the ARG (more details on simulations in Appendix S1).

Figure 3 shows the relationship between trees, SNPs and haplotype blocks arising from the first simulation—a neutral example capturing the ancestry of 10 genomes, sampled from a population of 100 haploid individuals, across 10 centimorgans (cM) of the genetic map (Appendix S1). SNPs were generated by infinite‐sites mutation with mutation at twice the rate of recombination. Despite the relatively short map and few individuals, this simulation is general because time and map distance both scale with population size (Hudson, 1990). Thus, the 268 generations taken for every part of the simulated genome to coalesce in a single common ancestor scales to 2.68 *N*, and the simulated map length scales to 10/*N*, where *N* is the effective size. Thus rescaled, this simulation shows a generic pattern, independent of population size.

The central panel of Figure 3 (middle panel, “SNPs”) shows the distribution of SNPs on the 10 sampled genomes, coloured according to the edge on which they arose (we illustrate eight edges with four or more SNPs each, out of 55 unique edges). Recombination events (24 in total) have divided the genome into 34 intervals due to nested recombination events (which split longer genealogies into nesting, inner intervals; Figure 3a). This illustrates how recombination modifies the coalescent (also see Figure A1 for a schematic representation of the process). The ARG can be decomposed into 24 unique marginal trees, some of which show an identical topology and differ only in timing (branch length); thus, 15 distinct topologies are shown in the Figure 3a (trees and corresponding regions on the genome labelled a–o; compare k_1_ and k_2_ for an example of genealogies that share topology but differ in depth, B in Figure 3).

The coloured blocks shown in the lower panel of Figure 3c illustrate the extent of each edge along the genome, and through time. The mutations arising on each branch are projected onto the edge at the time and genomic position that they arise. The number of SNPs arising on each edge is Poisson distributed, with the expected number proportional to the area of the edge; this area is the sum of the genomic lengths that each ancestor carries, and that is ancestral to the coalescence event that defines the branch. We emphasize that the visualized colour blocks represent *true* genealogies—and are independent from mutations. Because mutation, or SNP occurrence, is a random process, some regions may not carry any informative SNPs. For example, though edge *i* (light blue) is relatively well covered by nine SNPs, none of them fall in the shallow region to the left (C in Figure 3). Similarly, edge *ii* has only six SNPs, none of which happens to fall in the rightmost region (D). Ultimately, the distribution of SNPs sets a limit on what can be *inferred* from sequence data; edges without mutations will be invisible to us, and our ability to infer the length of a block depends entirely on where mutations happen to fall.

Each edge coincides with a specific coalescence event that brings together a specific set of lineages: in other words, edges are defined by both the coalescence event *and* the set of lineages. A single coalescence (i.e., a single ancestor) may generate multiple edges: the two genomes that come together in that event may carry a mosaic of ancestral material, in several combinations. A single coalescence event may even generate an edge that carries disjunct segments of the genome. This did not occur for any of the focal edges in the example of Figure 3, but is not unlikely, especially in a selective sweep. Conversely, two different coalescence events may happen to bring together the same sets of lineages; their edges could only be distinguished through the different times of coalescence.

Because each edge is generated by a single coalescence, it begins at the same time across its whole extent (so, edges are bounded by a horizontal line at their base in the lower panel of Figure 3). Recombination events split distal segments, thus limiting the span of the block along the map. Tracing back in time, edges must end in coalescence events that combine them with yet more descendants. These may occur at different times if there have been recombination events, so that the upper boundary is typically ragged.

Haplotype blocks overlap in their genomic extent, since multiple lineages exist at any time more recent than the most recent common ancestor (MRCA); this is shown by the overlapping 3‐D blocks in Figure 3c. Haplotype blocks will also overlap in the genome when edges are nested in the genealogy, giving rise to nested haplotype blocks. For example, edge *ii* (orange), which is ancestral to genomes 4 and 8, descends in the middle part of the genome from edge *i* (blue), which is ancestral to genomes 4, 7, 8 and 10. Thus, edge *i* is nested above block *ii* in Figure 3 (see also F for another example of nested haplotype blocks).

If we start at a particular site on the chromosome, and work along the genome, at some point an edge will be split by a recombination event. If the recombination occurs on the edge itself, the edge will persist, but probably with a different depth. If the recombination event occurs outwith the lineages that descend from the focal coalescence, but coalesces into those lineages, then the set of descendants will be augmented, and the edge will end. Conversely, if the recombination event occurs among the descendant lineages, then some descendants will be lost, and the edge will again end.

As we work out from a given locus, the incidence of recombination is proportional to the branch length, and so we expect that if a branch traces back deep into time, it will extend over a short region of the genome. Conversely, shallow branches will extend over a longer genomic span. This pattern is seen clearly in Figure 3c, where edges consist of segments that are either deep and narrow, or shallow and wide. However, this relationship is not *precisely* inverse; if it were, edges would tend to have the same area, whether they were deep or shallow, and hence would carry similar numbers of SNPs. In fact, the distribution of areas of blocks is highly skewed, and so most SNPs are on a few deep branches (see discussion on branch depth in Appendix S1).

Note that under the coalescent process, large numbers of sampled lineages rapidly coalesce down to a few, which are then likely to trace back deep into the genealogy. Thus, in a given region of the genome a substantial fraction of SNPs will fall on long, deep, branches, whereas the tips of the genealogy will be hard to resolve. Moreover, in a large sample, it is unlikely that different coalescence events will bring together exactly the same set of lineages by chance, so that we can usually identify unique coalescence events as corresponding to unique sets of lineages. This is one reason why haplotype‐based analyses can be particularly useful in disentangling genetic structure.

Figure 3 illustrates the simplest case of the standard coalescent with recombination. In reality, population structure and selection complicate genealogies. For example, in the island model, lineages either coalesce quickly within a deme, or escape to coalesce much further back in time. This exaggerates the tendency for genealogies to be dominated by a few long branches (Wakeley, 2009). Selective sweeps have a somewhat similar effect. In the classic case (Maynard Smith & Haigh, 1974), all lineages at the selected locus coalesce in the individual that carries the favoured mutation. Moving out from this locus, recombination frees lineages to coalesce much further back.

**FIGURE 4:**
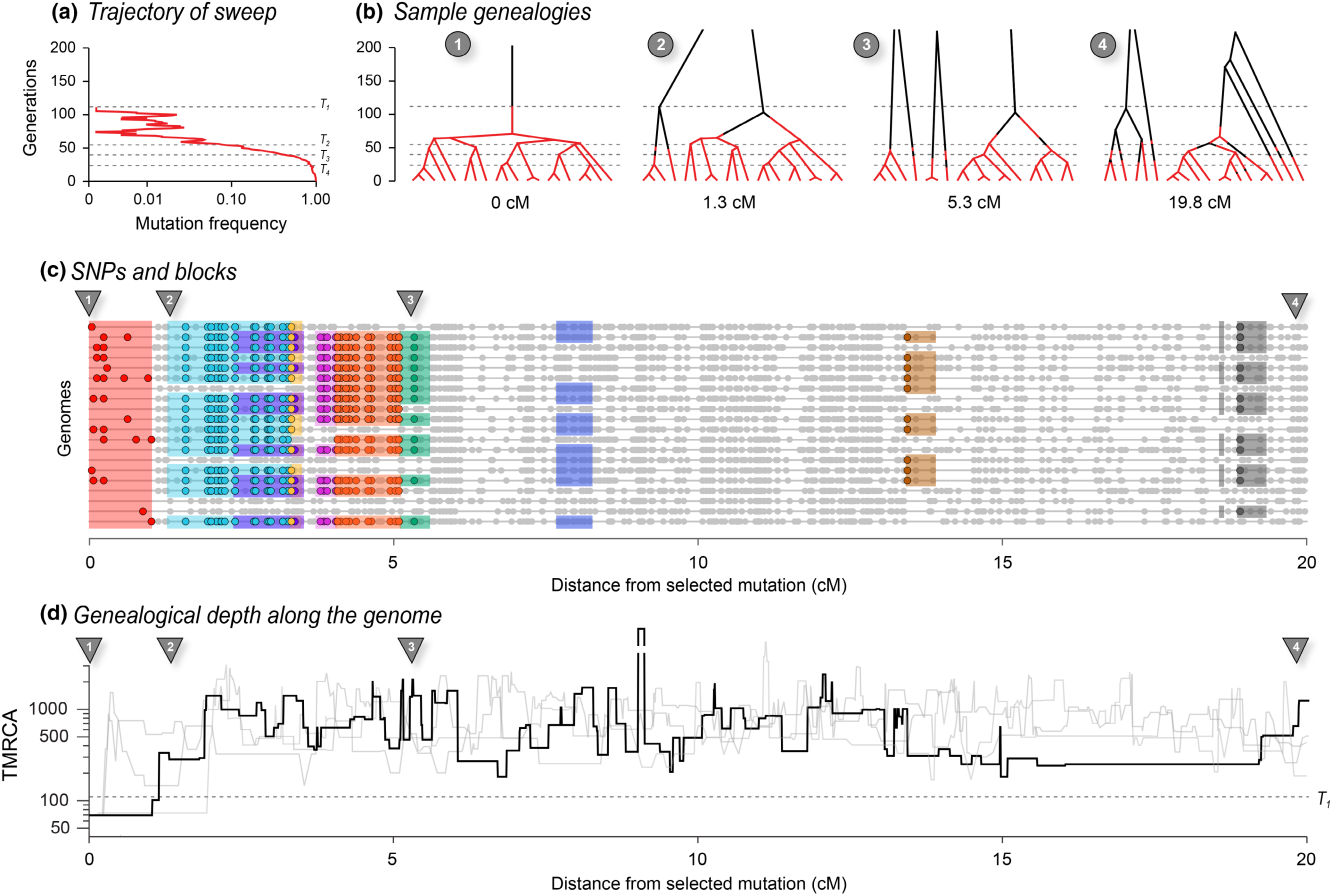
The effects of a recent selective sweep on linked genealogies. (a) A mutation with advantage 10% arose in a population of 400 haploid individuals, and swept to fixation in 110 generations, at which time 20 genomes were sampled; 20 cM of the genome is followed back in time, with the selected locus at the left; dashed lines (T_1_–T_4_) show times when the favoured allele was in one copy, at 10%, at 50% and at 90% (110, 53, 38 and 22 generations back). (b) Genealogies at positions 0, 1.3, 5.3 and 20 cM; branches are coloured in red when on the fitter background and black when on the ancestral background. Thus, changes in colour show recombination events that change the genomic background. Note that such events are unlikely when the allele is near fixation (i.e., at the base of the tree, below the lower dashed line) and, conversely, become common when the allele is rare, simply because it will almost always meet with the opposite background. Before the mutation occurs (i.e., above the upper dashed line) lineages must either trace back to that mutation (top left) or recombine out into the ancestral background; thus, all lineages must appear black above the upper dashed line (110 generations back). Note that the disjunct branches in trees 2–4 all coalesce further back in time, but only 200 generations are shown for visibility. (c) SNPs along the 20 sampled genomes. Nine of the most substantial branches are shown. (These have more than eight descendants, formed by coalescence more recently than the sweeping mutation, and have areas >0.5.) The red block at the left shows the region linked to the selected locus, which coalesces in a single common ancestor 69 generations back, just after the sweeping mutation arose. Grey dots show those SNPs that are not on these nine highlighted branches. (d) The time back to the most recent common ancestry (TMRCA) along the genome, on a log scale. The bold line shows the example simulated above, whilst the three grey lines show replicates, generated conditional on the same sweep; the break in the line shows an area where the TMRCA extends further back than the extent of the *y*‐axis. The dashed line across the plot corresponds to T_1_ in (a)

Figure 4 illustrates such a selective sweep (Appendix S1). The sweep greatly reduces diversity around the selected locus, because all lineages must trace back to the successful mutation (Figure 4b,1). This region of complete coalescence is shown in red, but note that it contains some diversity, due to mutation subsequent to the sweep. As we move away from the selected locus, lineages recombine out onto the ancestral background, and coalesce with the rest of the genealogy much further back (Figures 4b, 2, 3, 4). This process can be seen in the time to the MRCA (TMRCA) (Figure 4d), which jumps from a low value at the selected locus, through successive recombination events, back to a time that fluctuates around 4*N*_*e*_ = 800 generations, under the standard coalescent. However, the replicates in the lower panel show that there is considerable variation in this process, which sets a fundamental limit on our power to detect a sweep and estimate its properties.

At the selected locus, all lineages coalesce in the favoured mutation. Successive recombination events each free one or a few lineages from the new background, so that the exceptionally large and recent cluster gradually diminishes in size, until the genealogies follow a close to neutral distribution. Thus, edges with large numbers of descendants are associated with the sweep, and can be distinguished by the characteristic sets of SNPs that they carry; nine such edges are illustrated in Figure 4c.

We close this section by commenting on possible connections between our description of the ARG and practical inference. Stern et al. (2019) proposed a method that infers the allele frequency trajectory from the genealogy at the selected locus, which is assumed to be known. The extent of the focal edge along the genetic map gives additional information, with a predicted constant rate of recombination out into the ancestral background, at a rate equal to the frequency of the ancestral allele. Additional edges give more information: in particular, several lineages may coalescence early in the sweep, but then recombine out (e.g., the second genealogy in Figure 4b). This generates multiple long branches, whose distribution depends on 4*N*_*e*_ value (Barton & Charlesworth, 1998). There is considerable scope for using the extent of edges along the genome, as well as the genealogy at specific loci.

Nevertheless, we make two cautionary comments. First, there is considerable variability between different realizations, given the same trajectory (e.g., Figure 4d; i.e., the selective sweep itself is a stochastic process). Moreover, if the locus is identified from a genome‐wide scan, ascertainment bias will distort the ARG: indeed, sequence variation around a neutral locus that experiences a sweep *by chance* may be indistinguishable from a genuinely selected locus. This poses fundamental limits to our ability to estimate selection at a particular locus. Second, sophisticated methods based on simple scenarios will be confounded by deviations from the model. For example, the extent of reduced diversity along the genome is the inverse of the time taken to reach high frequency, but that may be greatly increased by population structure. The visualizations that we develop here may have the greatest value in allowing us to check whether the fine structure of a candidate region is actually consistent with some simple model. It remains to be seen how far the rich information contained in the structure will help us improve our inferences.

## 4 THE DEFINITION IN PRACTICE

Having defined haplotype blocks conceptually, we next consider the problem of inferring haplotype blocks from empirical data sets. Current sequencing and genotyping technologies make it straightforward to identify SNPs or small insertions/deletions, but it remains nontrivial to connect these to the haplotypes in which they are embedded. For that reason, sophisticated algorithms have been developed for phasing, imputing genotypes and inferring genealogies (Browning & Browning, 2009, 2013; Davies et al., 2016; B. Howie et al., 2011; Marchini et al., 2007). These tasks all engage different facets of the same problem, and rely to various extent on the haplotype structure. However, these methods tend to focus on phasing and stop short of inferring underlying haplotype structure and in particular the ARG, and haplotype blocks as we define them. In this section, we wish to focus on how haplotype blocks can be defined and visualized in practice. Given our ARG‐based definition, we used argweaver (Rasmussen et al., 2014) to analyse an empirical data set. We discuss other methods that use the ARG or approximations to it, the underlying assumptions of these methods and highlight where they could be extended to capture further information in light of our proposed definition of haplotype blocks as edges. Separately, in Box 3, we outline classes of simpler methods that use fixed genomic windows or genomic segments as a proxy for the haplotype block.

BOX 3 **Application and limits of the Li and Stephens model**

Li and Stephens (2003) (LS) proposed a hidden Markov model (HMM) framework that underpins a large number of existing inference methods. Originally developed to model patterns of linkage disequilibrium, it has since been widely applied to develop analytical tools and address empirical problems, such as phasing and imputation of genomic data (Browning & Browning, 2007; Howie et al., 2009; Li et al., 2010; Marchini et al., 2007; Stephens & Scheet, 2005), inference of population structure and demographic history (Hellenthal et al., 2014; Lawson et al., 2012; Steinrücken et al., 2019; Steinrücken et al., 2018), characterization of local admixture (Price et al., 2009; Sundquist et al., 2008), inference of local genealogies (Kelleher et al., 2019; Rasmussen et al., 2014; Speidel et al., 2019) and many more. The LS HMM framework is highly tractable and efficient. However, underlying assumptions make it incompatible with the haplotype definition we propose.

**FIGURE A2:**
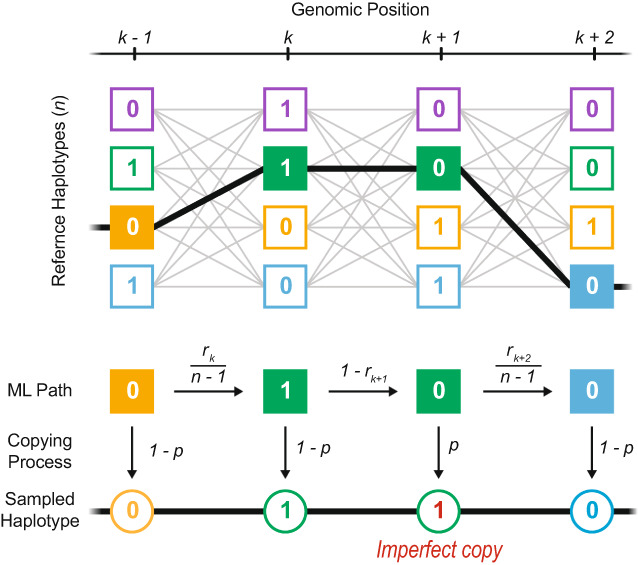
Schematic representation of Li and Stephens' (LS) hidden Markov model. A new haplotype can be sampled as an imperfect copy of *n* reference haplotypes (hidden states). To find the most likely path taken through the hidden states, the LS model works along the genome (*k* − 1, *k*, *k* + 1, …), calculating the probabilities of changes in the attributed haplotype. The transition probability to continue or switch the attributed haplotype is a function of the recombination rate (*r*) between adjacent sites, whilst the emission probability to copy the attributed allele with or without error is a function of the mutation rate (*p*). Moving along the genome, the LS model compares the probability of every possible copying path and infers the most likely one

The LS algorithm (Figure A2) requires a reference sample of haplotypes, or if presented in a sequence, previously observed haplotypes. It gives a framework to decide whether some focal haplotype represents (i) an entirely new haplotype or (ii) a mosaic of previously encountered haplotypes, and determines the breakpoints and transitions in this mosaic. Whilst the LS model captures genetic relatedness among chromosomes through recombination, it assumes that the reference haplotypes are known. This would be valid in a selection experiment, if we know the founder genomes; in this case, blocks are defined by identity by descent (IBD) to this reference population. However, if we only have contemporary genomes, the reference panel is an approximation. Second, the model assumes that genomic states depend solely on the immediately preceding site. This is also an approximation, since in the true ARG, recombinant lineages can coalesce back to any lineage that existed in the preceding genome, which yields disjunct haplotype blocks.

The full ARG contains all the information needed to apply the haplotype block definition to empirical data sets. Therefore, one could start by inferring the ARG (or even a sequence of genealogies along the genome) from a sample of sequences, identifying important edges on that ARG, and consequently the haplotype blocks that descend from those edges. argweaver (Rasmussen et al., 2014) and its extension argweaver‐d (Hubisz et al., 2020) are among the most powerful tools for direct inference of the ARG. One practical speed‐up employed by argweaver is to discretize time, effectively making the ARG space finite by limiting recombination and coalescence events to discrete time points. Further, argweaver uses an approximate model, Sequentially Markov Coalescent (SMC; McVean & Cardin, 2005; extended by Marjoram and Wall 2006) to sample from a distribution of the ARG. While making inference more tractable, the SMC precludes the inference of disjunct blocks, because only one immediately prior state is considered as one moves along the genome. However, even with these key innovations, inference of the “full” ARG remains computationally expensive, making argweaver feasible for up to ~50 samples.

**FIGURE 5:**
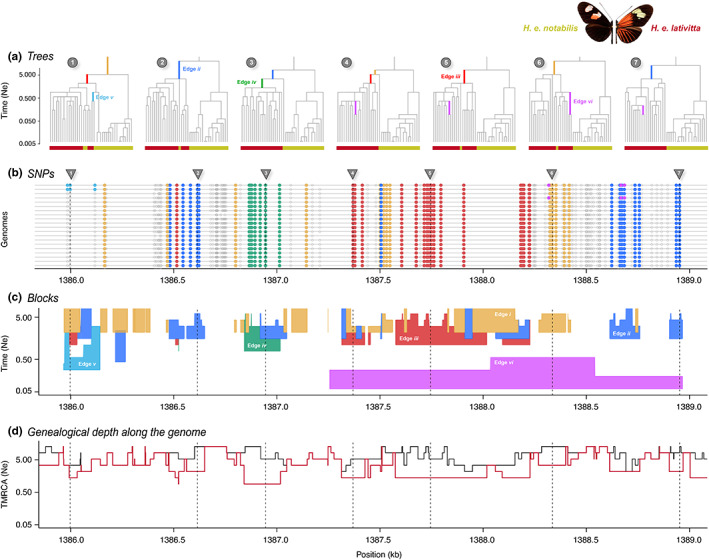
Visualization of haplotype blocks based on application of argweaver to the *optix* region of *Helconius erato* butterflies (2*n* = 20). (a) Genealogical trees at each genomic point, marked by arrows and corresponding numbers. Genomic points are chosen to show all coloured edges as branches on the corresponding marginal trees. Red labels: *H. e. lativitta* samples; yellow labels: *H. e. notabilis* samples. Branches on trees are coloured according to the corresponding edges in (c). Both highland *H. e. notabilis* (2*n* = 20) and lowland *H. e. lativitta* (2*n* = 20) populations are included in the analysis so that we can estimate the length of branches that are ancestral to all *H. e. lativitta* samples (2*n* = 20), but (b)–(d) only exhibit features related to the *H. e. lativitta* population. (b) Genomic location of SNPs for all 20 *H. e. lativitta* haploid samples. Out of a total of 137 SNPs, only those that explain the six substantial edges are coloured accordingly. Edges are defined as substantial if three or more SNPs occur on them. Two out of six edges (*v* and *vi*) are explained by doubleton SNPs, whereas the rest are fixed within the *H. e. lativitta* samples. SNPs that do not appear on any significant edge are coloured grey if they have higher allele frequency within the samples, white otherwise. (c) Visualization of haplotype blocks as edges similar to Figure 3, plotting blocks along the genomic (*x*‐axis) and temporal span (*y*‐axis). Since edges always originate at a fixed coalescent time point, the bottom line of the block is always smooth. The ragged tops of the blocks denote the length of the edges interrupted by recombination events. Note that edges can also be disjunct due to one or more samples and recombining out and back into the same lineage. (d) Relative TMRCA half‐life estimates along the genome: Black: total TMRCA to all 40 samples; red: TMRCA to only *H. e. lativitta* samples

To illustrate our concept, we applied argweaver to infer the ARG from an empirical, phased data set in *Heliconius erato* butterflies. The data set was generated by haplotagging, a technique for producing linked‐read sequence data (Meier et al., 2021). We focus on the genomic region containing the gene *optix*, where a selective sweep was previously inferred using site‐based statistics (Figure S3: Appendix S2). For comparison, we also sampled ARGs from a neutral background locus (Figure S3: Appendix S2). Figure 5 shows a focal region (~3 Mb long) located ~100 kbp upstream from *optix* that may correspond to a distal regulatory hub controlling the distinctive wing rays—“Ray” and “Dennis” elements (Wallbank et al., 2016), possibly corresponding to *obs132*, *LR1/2* and *obs214* (see Lewis et al., 2019)—at which all lowland *H. e. lativitta* butterflies (red labels in Figure 5a) share a haplotype (Meier et al., 2021). To run argweaver, we used 𝜇 = *r* = 2.9 × 10^−9^ and *N*_*e*_ = 1.94 × 10^6^ (calculated by estimating 𝝅 from the neutral region) and 30 discrete exponentially distributed time points (Figure S1: Appendix S2). The key step for visualizing haplotype blocks is identifying unique edges based on the sampled ARGs. We identified edges using custom scripts to parse marginal trees along the genome in order to identify branches that originate at a particular coalescent time point (say, *t*_1_), and are ancestral to a fixed set of individuals (say, *x*_1_, *x*_2_, *x*_3_; see Tables S1 and S2: Appendix S2). This set of branches represents an edge that is defined by {*t*_1_}, {*x*_1_, *x*_2_, *x*_3_} and the trees that contain the above branches (see branches in Figure 5a coloured according to haplotype blocks as edges in Figure 5c). Here, we visualize haplotype blocks in *H. e. lativitta* only and chose to focus on the six edges that are supported by three or more SNPs (Figure 5b: SNPs; Figure 5c: haplotype blocks as edges; see Table S2: Appendix S2, for a list of all edges supported by SNPs). We then visualized haplotype blocks based on these edges, including both their genomic span (i.e., the tree‐spans along the genome containing the edge) and temporal span (length of each tree branch that constitutes the edge; Figure 5c). To contrast these observations with the neutral background locus we additionally plotted TMRCA trajectories for both regions (Figure S3: Appendix S2). In the neutral region we did not observed regions with shallow coalescence despite some variation.

We find a rich structure of haplotype blocks in the focal region. First, in regions of shallow coalescence, we see structured uninterrupted haplotype blocks that carry SNPs fixed in all *H. e. lativitta* samples (Figure 5). For example, the shallowest coalescence region constitutes the uninterrupted haplotype block defined by edge *iv*, and is supported by nine SNPs. Adjacent to edge *iv*, we find the largest (in genomic span) haplotype block defined by edge *iii* and supported by 22 SNPs, which coincides with a putative selective sweep that was previously identified using the omega statistic (Lewis et al., 2019; Wallbank et al., 2016). Unlike edge *iv* (green), edge *iii* (red) exists as disjunct blocks separated primarily by recombination events that shift the total coalescence within *H. e. lativitta* samples to occur slightly further back in time (see Trees 3–6 for reference). Moving along in both directions from this region, we observe other haplotype blocks, exhibiting different histories (originating deeper in the past) spanning as disjunct blocks along the entire genomic region and supported by observed SNPs (edges *i* and *ii*). Although the SMC precludes argweaver from inferring disjunct coalescence events, disjunct blocks (especially, edge *i*) may be an artefact of the way we identify unique edges from the argweaver output, together with discretization of time points (in principle, they could be distinct coalescence events but are forced to coalesce at the same time). Moreover, these blocks can also stem from including the *H. e. notabilis* population in our analysis (Trees 1, 2, 5 and 6 have one or more *H. e. notabilis* individuals clustering together within the *H. e. lativitta* population), and hence are present as nested blocks within edges *iii* and *iv* in the central regions of the shallowest coalescence. In future, it would be interesting to examine if additional evidence can suggest older, historical sweep events to explain the disjunction of these blocks, or whether they are simply remnant structures/signatures from other stochastic events. It is important to note that four out of the six substantial edges drawn here are supported by SNPs fixed within the *H. e. lativitta* samples, and spans in disjunction (except edge *iv*) throughout the region. This is in agreement with theoretical expectations in a swept genomic region where uninterrupted edges are expected to exist with multiple SNPs supporting them (edge *iv*, nine SNPs, *iii*, 22; *ii*, 16; *i*, 14). In addition to these four blocks, we also see more recent blocks with greater spans, explained by singletons (see Figure S6: Appendix S2) and doubletons (*edges v* and *vi* in Figure 5). Edge *vi* specifically spans the furthest along the genome, since it is formed by a coalescent event between only two samples, suggesting that there has not been sufficient time for recombination to break it down.

Our argweaver analysis shows the complex relationships between SNPs, edges and haplotype blocks inferred from real data, but also demonstrates the possibility of visualizing haplotype blocks as edges, and utilizing statistics from these blocks as a signal to make further evolutionary inference. This could potentially produce alternative hypotheses, such as multiple selective sweeps occurring in the proximity of each other, or differentiate between sweeps and random shallow coalescence events. However, we should point out several limitations to such analyses. First, since there is an infinite number of possible genealogical histories, inference of the full ARG comes with a degree of uncertainty. Specifically, argweaver estimates a coalescent model from the data (set of SNPs) and produces a distribution of trees that is consistent with the data, but only supported strongly at and around the SNPs. In other genomic regions, the model produces a random distribution of trees given the parameters that are still consistent with the data but not necessarily supported by any SNPs (see Figure S8: Appendix S2, for comparison between haplotype block structures between Markov chain Monte Carlo [MCMC] iterations). This suggests inferences should only be made only from edges robustly supported by the SNP configuration, rather than the full ARG. Due to its computational tradeoffs, argweaver inference can also be prone to differences in user choices such as discrete time points, recombination/mutation rates, estimation of effective population size and MCMC parameters. Despite these limitations, features of haplotype block structures from our analysis can carry potentially important features in non‐neutral regions of the genome. Here, we simply demonstrate with a small example one way to identify significant edges and haplotype blocks from empirical data. Although beyond the scope of this paper, we hope that the rich haplotype structure revealed here can spur development of new methods that take advantage of different layers of information.

The computational requirement and feasibility of argweaver were addressed by two other methods—tsinfer and relate (Kelleher et al., 2019; Speidel et al., 2019)—that attempt to approximate the ARG in much larger populations with thousands of samples by focusing on topology (or “succinct tree sequences”), rather than a full inference of the ARG. They do so by representing genomes as a series of tree topologies: relate as distinct trees; tsinfer as “tree sequences” connected via ancestral haplotypes. Both achieve this remarkable speed‐up by relying on the Li and Stephens' hidden Markov model (Li & Stephens, 2003; see Box 3 for further details) to infer local pairwise distances (relate) or ancestral haplotypes (tsinfer). As an added advantage, tsinfer doubles as an efficient, lossless compression algorithm by indexing population genomic variation as SNPs‐on‐trees as opposed to the traditional (and highly redundant) SNP‐by‐individual matrix (implemented as a tskit library; Kelleher et al., 2019). Put another way, the tree sequence encoding can fully capture the variation data in entire populations, for a fraction of the storage space. Such a representation also effectively encapsulates a number of population genetics summary statistics (Kelleher et al., 2019; Ralph et al., 2020). These developments may prove essential, as sequencing of entire national populations increasingly becomes routine.

Among practical methods, tsinfer and relate are the state‐of‐the‐art in representing large populations. All three approaches, including argweaver, approximate some aspects of the ARG well, and give an accurate distribution of coalescence time under simulation of the standard coalescent (Brandt et al., 2021). For our purposes, they are also useful approximations to the ARG that highlight some of the key advantages we wish to emphasize in our haplotype block definition. For example, relate presents a suite of statistics that goes beyond SNP information. One advantage of relate is that branches are dated, as opposed to a strict encoding of topology alone in tsinfer. Having dated branches allows, among other things, the possibility of estimating temporal changes in mutation rates. Another useful feature, in our view, is placement of SNPs onto branches, which is the essential feature that distinguishes haplotype blocks from each other under our definition, even though our definition is independent of the SNPs themselves.

We note that efforts are already underway to bridge across methods and address their limitations. For instance, tsdate now adds coalescence times estimates and branch lengths from tsinfer's output (Wohns et al., 2021). In the context of our exploration of haplotype blocks and their overlapping structure (see C and D in Figure 3), we have noted that they may not be accurately captured under the Li–Stephens models in tsinfer and relate, in a way that may bias the inferred ARG. However, this is an open question, so more work is needed to understand how different methods perform across a range of parameters relevant to nonmodel organisms.

In summary, there has been a recent spurt in innovation in genealogy/ARG‐based methods. Among these, argweaver arguably comes closest to inferring the full ARG, but at considerable computational cost. Both tsinfer and relate are robust and scalable to thousands of samples with minimal, reasonable trade‐offs, but infer haplotype blocks only as an incidental output. Ultimately, we hope our discussion here will encourage development of new methods to infer haplotype blocks as we define them, and to use these for further explanation and inference.

Assuming that a method becomes available for inferring blocks as we have defined them, there are still practical considerations that we will need to face. For example, we see from Figures 3, 4, 5 that haplotype blocks, defined via edges in the ARG, have a complex structure, tracing back in time for a number of generations that varies along their span (e.g., blocks *ii* and *iii*). This makes it hard in practice to define the extent of haplotype blocks in any simple way, especially since they may be disjunct. Should this be their maximum length, or should it rather be weighted by the depth? It is not clear which description would be better for inference and this may even depend upon the specific process that we wish to infer. These kinds of issues could be investigated by estimating parameters under a variety of specific models in which case we can evaluate the strength and weaknesses of different descriptions of haplotype structure in characterizing different processes.

## 5 CONCLUSIONS AND FUTURE DIRECTIONS

In this article, we have outlined a definition of the haplotype block as an edge on the ARG, explored the implications of the definition with simple simulations, and considered how current methods can infer such blocks from empirical data. In our view, haplotypes and haplotype blocks should be the core concepts through which we understand population genetic processes. Under this view, it follows that, ideally, genomic data sets should come directly as resolved haplotypes, rather than diploid genotypes that require phasing and further processing. We therefore welcome new developments in linked‐ and long‐read sequencing techniques, analysis software, and visualization tools that are designed with sequencing and population data sets in mind (Davies et al., 2021; Meier et al., 2021).

Our simulations and empirical example show that haplotype blocks contain rich information about the demographic and selective history of the locus. Making the most of this information will require a fundamental rethink of our linear, reference‐based genome assemblies, and a move towards a graph‐ or tree‐based assembly standard to take advantage of their capability to natively encode variation (Eggertsson et al., 2017; Hickey et al., 2020). We will also need new concepts and vocabulary to describe features in these graphs (e.g., super‐graphs and “bubbles”; Cheng et al., 2021; Turner et al., 2018; Weisenfeld et al., 2017) informed by a robust understanding of the generative process discussed above, and we need to align our mental models with inference schemes and their encoding (as in, for example, tsinfer). For that reason, we hope our discussion here can focus our effort towards this new standard, as haplotype‐resolved sequencing becomes routine.

## AUTHOR CONTRIBUTIONS

All authors conceived the ideas and contributed to the writing of the manuscript. NHB conducted the simulations and AP implemented the practical example.

## CONFLICT OF INTEREST

The authors declare that they have no conflicts of interest.

## Supporting information


Appendix S1:

Appendix S2:

## Data Availability

The *Heliconius* sequence data used in this paper are associated with Meier et al. (2021) and were previously deposited at the National Center for Biotechnology Information Sequence Read Archive under the BioProject accession no. PRJNA670070.
