# Supplementary material for: On the origin and structure of haplotype blocks
Source: Mol Ecol. 2022 Dec 15;32(6):1441–57. doi: 10.1111/mec.16793 (PMC10946714; doi:10.1111/mec.16793)
Supplement: Supplementary file 1 — Appendix S1: [file MEC-32-1441-s002.pdf]

Summary of the simulation

The simulation generates the ancestral recombination graph, by tracking the ancestors of a sample of genomes back through time, until all ancestral genomes are ancestors to the whole sample. The simulation can be conditional on a selective sweep, which is defined by the numbers of copies of the favourable allele in the population. A Wright-Fisher model with constant populations size $2N$ haploid genomes is assumed. A region of genome of map length R is followed, with the selected locus at the leftmost point (i.e., at 0). For simplicity, we allow at most one crossover, with probability R; we simulate $R << 1$, so this is close to the case with no interference between crossovers. Once the ARG is constructed, genealogies along the genome can be constructed, and their branches identified. Neutral SNP can be added, assuming infinite-sites mutation; each SNP is associated with a branch in the ARG.

Each ancestor is represented by three elements: its genotype at the selected locus; a list of *junctions*, and a list of the sampled genomes that descend from each interval. For example, {0, {0.02, 0.035}, {{1, 3}, {}, {5, 6, 7}}} represents a genome divided into three regions {0, 0.02}, {0.02, 0.03}, {0.035, R}, which are ancestral to sampled genomes {1, 3}, {}, {5, 6, 7}, respectively; the selected locus at position 0 carries allele 0. For the neutral case, we simply set this genotype to 0 throughout.

In the sampled generation, a sample of k genomes is represented as {{0, {}, {{1}}}, …, {0, {}, {{k}}}: the j' th genome is ancestral to itself, (j), over its whole length. Stepping back, crossovers are generated for each genome, drawn from a Poisson with expectation R, and uniformly distributed. To be efficient, these are generated in a single draw. Genomes that experienced a crossover are replaced by two parent genomes. For example, if there were a crossover at j_1, {0, {}, {1}} would be replaced by genomes {0, {j_1}, {{1}, {}}} and {X, {j_1}, {{}, {1}}}. Here, the genotype X is chosen randomly with probability equal to the allele frequency in the whole population. Stepping back in time, coalescence is simulated by assigning each genome a parent with the same allelic state. If the population in the previous generation carries k copies with allele $X = 1$, then each current genome is assigned a random integer in {1, k}, and similarly for allele $X = 0$. Parent genomes are then assigned by merging ancestries. For example, if current genomes {0, {j_1}, {{1}, {}}} and {0, {j_2}, {{3}, {2, 3}}} share a parent, and $j_1 < j_2$, then that parent is assigned as {0, {j_1, j_2}, {{1, 3}, {3}, {2, 3}}}. Note that coalescence can only occur within a genetic background (defined by $X = 0$ or 1), and that multiple coalescences between multiple genomes can occur: we simulate the Wright-Fisher model, not the standard coalescent, which only applies in the limit of a large population. This is important, since in the early stages of a sweep, the new allele is present in few copies. In each generation, the list of ancestral genomes is tidied by deleting genomes that are not ancestral to the sample, and deleting junctions that separate regions with the same ancestry. The algorithm is iterated back, until every ancestor is ancestral to the whole sample, which implies that all genealogies have reached their single common ancestor.

Very large populations can be simulated, since we only track ancestors. The computation is limited by the number of ancestors, which increases with $2N_e R$, and the time to complete coalescence, whioch increases with $4N_e$. For a given $2N_e R$, it is most efficient to choose a modest N_e and a large R, but N_e should not be so small that results deviate from the standard coalescent. This can be determined by making some runs for larger N_e, and by checking against theoretical predictions for

the standard coalescent.

In the neutral case, the allelic state is simply set to $X = 0$. To simulate conditional on a sweep, the trajectory is first generated, by simulating forwards from a single mutation, according to the Wright-Fisher model, and conditioning on fixation. The trajectory is then augmented back in time, by setting it at one copy into the indefinite past. (It is not obvious what the correct choice should be here, but since the genealogy at the selected locus must have coalesced completely at the time of the sweep, and since linked lineages are unlikely to coalesce into the ancestor of the mutation (assumed to be in a single copy) the choice should not make much difference). By first drawing the trajectory, and then simulating coalescence and recombination conditional on it, we can separate random variation in the trajectory, from random variation in the outcome for a given trajectory. We can only infer properties of the single actual trajectory, which may well deviate substantially from expectation.

The core structure, which defines the ARG, is the list of ancestors, traced back through time. From this, we generate a list of all distinct junctions, and thus, of all intervals along the genome; for each of these, a genealogy is constructed. We then construct a list of branches, each branch being generated by a coalescence event that occurs at a specific time, and that brings together a specific set of sampled genomes. For each branch, we list the regions of genome that it covers, in which generation, in the form $\{\{t, \{\{r_0, r_1\}, \ldots\}\}, \ldots\}$; note that regions may be disjunct. The full list of branches covers the whole ancestry.

Neutral SNP are generated, assuming infinite-sites mutation. For every branch, SNP are generated with density μ / r per map length; these are then combined into a list of SNP, each defined by its time of origin, its map position, and its branch, $\{t, x, b\}$. In practice, of course, we must infer branches from the SNP that they carry.

Notes on executing the code

To execute the examples here, initialise the accompanying package **GenealogiesNewV5** as well as this notebook; this sets up definitions for handling genealogies.

This notebook is large, but could be reduced by deleting graphics, or converting them to bitmap.

Data from specific examples in the paper are stored as Mathematica definitions:

Neutral example: ”pl10 31 Oct”, “snp10full 3 Dec”

Sweep example: “Big sweeps s=0.1, 2N=400 R=0.2 20 genomes 2 Jan”

For the sweep example, replicate genealogies were generated, and stored in “Big sweeps s=0.1, 2N=400 R=0.2 20 genomes reps 4 Jan”. This is not provided, since it is enormous (3.9Gb). However, replicates conditional on the given sweep can be generated quickly.
Make sure to use SetDirectory to specify where these are stored on your machine.

Neutral case

Example with 10 genomes, sampled from 2N=100 on R=0.1 (i.e. 10cM); SNP generated with μ/r=2

Setting up, and saving results

This is very fast. It takes 268 generations until complete coalescence:

```
In[971]:= Timing[time = 0; pl10 = makeC[10, 0.1, 100]; Length[pl10]]
Out[971]= {0.377784, 269}
```

The simulation stores individuals that are represented as $\{X, \{r_{1,2}, \ldots\}, \{a_{1.}, a_2, \ldots\}\}$, where X is the genotype at the selected locus, $\{r_{1,2}, \ldots\}$ lists the breakpoints, and $\{S_{1.}, S_2, \ldots\}$ lists the sets of sampled genomes that each interval is ancestral to. The list below goves the lengths of these sets, for individuals 10 generations from the end, when there are 8 ancestral genomes, and much of the genome has coalesced (represented by sets S of length 10).

```
In[399]:= Map[Length, pl10[[-10, All, 3]], {2}]
Out[399]= {{0, 3, 0, 6, 10, 0, 10, 0}, {0, 10}, {0, 10, 0}, {0, 10, 0},
  {0, 10, 7, 10, 0, 10, 4, 0}, {0, 10, 0}, {10, 0}, {0, 10, 0}}
```

The # of genomes that carry any ancestral material drops rapidly at t=100, but then increases. The number of ancestral lineages would keep fluctuating even after full coalescence.

```
In[976]:= ListLinePlot[Length /@ pl10,
  PlotRange -> {{1, 300}, {1, 10}}, PlotStyle -> PointSize[0.01]]
```

Out[976]=

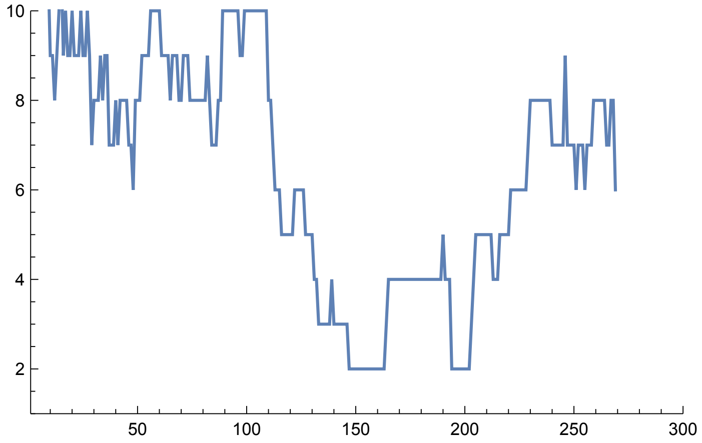

This saves the simulated ancestry (pl10)

```
Save["pl10 31 Oct", pl10];
```

This reads the data back in, to recover this example.

```
<< "pl10 31 Oct";
```

Notes on definition of branches

Initially, branches were defined by their descendant genomes. However, this may conflate branches that bring together the same lineages via different coalescence events. The simplest

solution is to also require distinct branches to be generated at different times. There is still the possibility that two coalescence events occur in the same generation and bring together the same lineages, but this seems very unlikely; it is not clear how to tag coalescence events to avoid this.

New functions are defined with the suffix ... Full that distinguish blocks that originate at different times, t. branchListFull returns branches in the form $\{S, t, \{\{r_1, r_2\}, \ldots\}\}$, allowing for the possibility that they may fall in multiple intervals (though this does not happen in this example). posBlockFull[pop, $\{S, t, \{\{r_1, r_2\}, \ldots\}\}$, R] returns the instances of the branch in the form $\{t, \{r_1, r_2\}\}$. These ..Full versions should be used throughout.

Making the genealogies

This sets up a list of the 34 genealogies along the 10cM stretch of genome:

```
In[417]:= Timing[gl10 = makeGenealogy[pl10, #, 0.1] & /@ intervals[pl10, 0.1];]
Out[417]= {2.04559, Null}
```

These are the 34 non-recombined intervals:

```
In[812]:= intervals[pl10, 0.1] // TableForm
Out[812]//TableForm=
```

0	0.000485901
0.000485901	0.00215397
0.00215397	0.00269484
0.00269484	0.00368421
0.00368421	0.00868471
0.00868471	0.00912131
0.00912131	0.0140003
0.0140003	0.015999
0.015999	0.0184439
0.0184439	0.0189914
0.0189914	0.0193689
0.0193689	0.021544
0.021544	0.0216572
0.0216572	0.0253309
0.0253309	0.0446031
0.0446031	0.0453142
0.0453142	0.0494893
0.0494893	0.0497073
0.0497073	0.0505236
0.0505236	0.050558
0.050558	0.0607542
0.0607542	0.0615051
0.0615051	0.0625631
0.0625631	0.0631933
0.0631933	0.0712151
0.0712151	0.0735098
0.0735098	0.0745833
0.0745833	0.074802
0.074802	0.0779924
0.0779924	0.0786718
0.0786718	0.0826169
0.0826169	0.0965451
0.0965451	0.0991522
0.0991522	0.1

This is a movie of the genealogies along the genome. Numbers label the branches, and the vertical scale is proportional to coalescence times. The vertical axis is always 270 (the time for all of the genome to coalesce.

In[2041]:=
```
ListAnimate[Show[PlotGenealogy[#, NodeFunction -> plotCoords[1]],
    PlotRange -> {{0, 11}, {0, 270}}, AspectRatio -> 0.7] & /@ gl10]
```

Out[2041]=

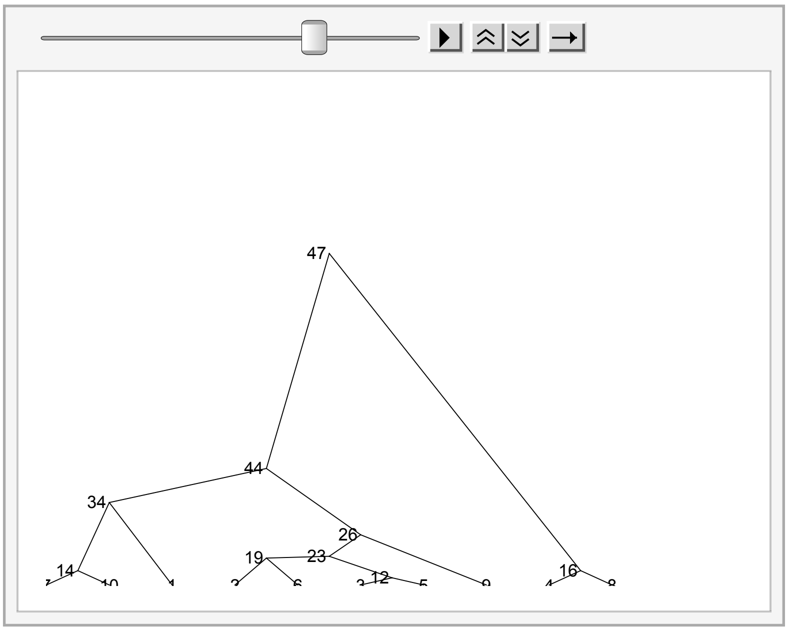

There are 34 intervals, 24 distinct genealogies, and 15 distinct topologies:

In[3202]:=
```
Length /@ {gl10, Union[gl10], Union[gl10 /. {{_Integer, {}} -> {τ, {}}}]}
```

Out[3202]=
```
{34, 24, 15}
```

Throwing down SNP: μ/r=2

This sets up a list of the branches (branchList); generates SNP, listing them as {t, x, k} for branch ♯k (makeSNP), and throws them onto the 100 genomes (addSNP):

In[2083]:=
```
blf10 = branchListFull[pl10, 0.1];
snp10full = makeSNP[posBlockFull[pl10, #, 0.1], 2] & /@ blf10;
pop10full = addSNP[blf10, snp10full];
```

This saves this set of SNP; the specific example used can be recovered using <<“snp10full 3 Dec”

In[2086]:=
```
Save["snp10full 4 Dec", snp10full];
```

In[2068]:=
```
<< "snp10full 3 Dec";
```

There are 87 SNP on 34 intervals. Each SNP is represented on average 254/87 times:

In[2087]:=
```
{Length /@ {Flatten[pop10full, 1], Union[Flatten[pop10full, 1]]},
 Length[intervals[pl10, 0.1]]}
```

Out[2087]=
```
{{254, 87}, 34}
```

This is the SFS:

In[2088]:=
```
sfs = Last /@ Tally[Flatten[pop10full, 1]];
Tally[sfs] // Sort
Histogram[sfs]
```

Out[2089]= {{1, 33}, {2, 13}, {3, 8}, {4, 16}, {5, 5}, {6, 5}, {7, 5}, {8, 1}, {9, 1}}

Out[2090]=

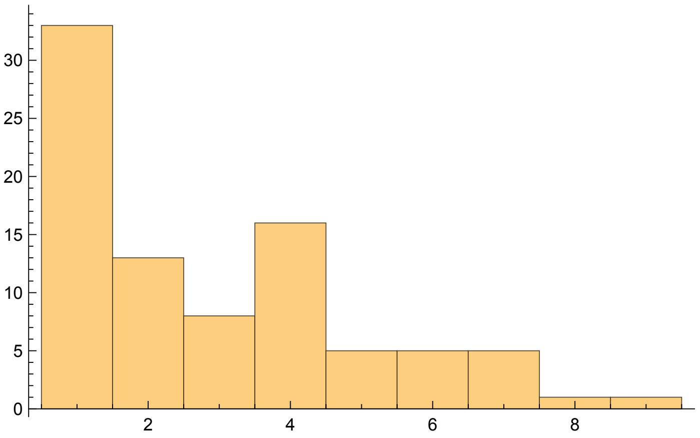

Finding the 9 branches with ≥4 SNP

There are 87 SNP on 54 branches, of which 53 are on the 9 branches that have ≥4 SNP:

In[2091]:=
```
big2full = Pick[Range[Length[blf10]], (Length[#] ≥ 4) & /@ snp10full];
{{Length[blf10], Length[big2full]},
  {Total[Length /@ snp10full], Total[Length /@ snp10full[[big2full]]]}} //
 TableForm
```

Out[2092]//TableForm=

54	9
87	53

These are the index; the # SNP (up to 10); the timespan; and the width on the map. There is an inverse relation between map length and timespan for these 9 longest branches

In[2093]:=
```
tt = {big2full, Length /@ snp10full[[big2full]],
    branchLength[posBlockFull[pl10, #, 0.1]] & /@ blf10[[big2full]],
    branchWidth[posBlockFull[pl10, #, 0.1]] & /@ blf10[[big2full]]};
tt // TableForm
```

Out[2094]//TableForm=

1	3	4	9	17	25	28
10	4	4	8	6	4	9
64	64	7	184	183	49	248
0.0637979	0.0180866	0.0984601	0.0107261	0.00815806	0.0240657	0.0[illegible]

In[2095]:=
```
ListLogLogPlot[Flatten /@ tt⟦{3, 4}⟧ // Transpose, PlotStyle -> PointSize[0.02],
 PlotRange -> All, AxesLabel -> {"time", "map\nlength"}]
Fit[Log[Flatten /@ tt⟦{3, 4}⟧ // Transpose], {1, t}, t]
```

Out[2095]=

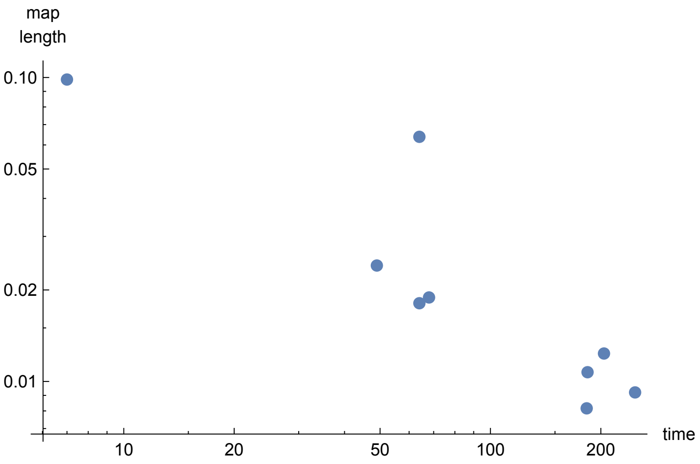

Out[2096]= $-0.870116 - 0.690694\, t$

inverse relation between block length and timespan

This was generated from a different example, in which all 100 genomes were sampled. It is included to support the argument that, because the slope of the relation between map length and time span is weaker than inverse (i.e. slope > -1), deep branches will tend to have a larger area and carry more SNP.

This generates a table txal of the time spanned by the branch; the mean map length covered by it (averaged over generations); and its area (which determines the expected # of SNP that it carries). txalm takes the mean values, when branches are grouped by timespan.

In[287]:=
```
<< "pl2 27 Oct";
tb = branchListFull[pl2, 0.1];
txal = (pb = posBlockFull[pl2, #, 0.1];
     {Length[Union[pb⟦All, 1⟧]],
      Mean[pb⟦All, 2⟧.{-1, 1}], Total[pb⟦All, 2⟧.{-1, 1}]}) & /@ tb;
txalm = Mean /@ GatherBy[txal, Log[#⟦1⟧] &];
```

It is not obvious which direction causality runs, and so we plot map length against the time spanned by the branch (left) as well as time span against map length (right). The 482 branches are shown by blue dots, and the mean for each timespan is shown by the red dots. The two fits (top, bottom row in the table) are to the blue vs the red dots. In each plot, the black line is $\sim t^{-1}$, to indicate the slope expected with a simple inverse relation. There is an inverse relation, but the least-squares fit is $x \sim t^{-0.68}$(left), or $t \sim x^{-0.54}$rather than the expected simple inverse.

Here, the exponent has magnitude less than 1 due to regression to the mean. Suppose that we have two variables, $\log(t) = y + v_t$ and $\log(x) = \frac{1}{y} + v_x$, which are determined by an underlying inverse relationship plus independent random errors with variances $v_t,\ v_x$, then the regression coefficient of $\log(x)$ on $\log(t)$ is $-\frac{v_y}{v_t+v_y}$, and of $\log(t)$ on $\log(x)$ is $-\frac{v_y}{v_x+v_y}$ - both of which have magnitude less than 1.

In[294]:=
```
GraphicsRow[{Show[ListLogLogPlot[txal[[All, {1, 2}]]],
    ListLogLogPlot[txalm[[All, {1, 2}]], PlotStyle -> Red],
    LogLogPlot[0.3 t^-1, {t, 1, 500}, PlotStyle -> Black],
    AxesLabel -> {"time\nspan", "map\nlength"}],
  Show[ListLogLogPlot[Reverse /@ txal[[All, {1, 2}]]],
    ListLogLogPlot[Reverse /@ txalm[[All, {1, 2}]], PlotStyle -> Red],
    LogLogPlot[0.4 x^-1, {x, 10^-4, 0.1}, PlotStyle -> Black],
    AxesLabel -> {"map\nlength", "time\nspan"}]}]
{{Fit[Log[txal[[All, {1, 2}]]], {1, t}, t],
    Fit[Log[txalm[[All, {1, 2}]]], {1, t}, t]},
  {Fit[Log[Reverse /@ txal[[All, {1, 2}]]], {1, r}, r],
    Fit[Log[Reverse /@ txalm[[All, {1, 2}]]], {1, r}, r]}} // Transpose // TableForm
```

Out[294]=

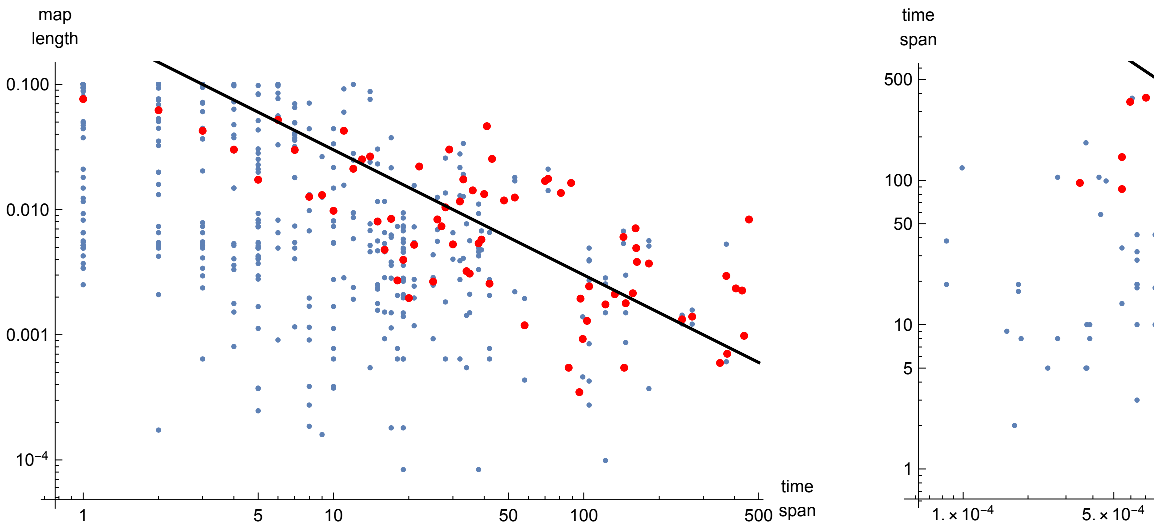

Out[295]//TableForm=

$-3.16957 - 0.681402\, t$	$-0.465778 - 0.542427\, r$
$-2.62644 - 0.656533\, t$	$-0.00112289 - 0.739972\, r$

The key question is, what kind of branches carry the total ancestry (measured by the area of the branch, and proportional to the expected # of SNP). The area of a branch increases with its timespan (as $\sim t^{0.34}$), and with the map length that it spans.

In[306]:=
```
GraphicsRow[{Show[ListLogLogPlot[txal[[All, {1, 3}]]],
    ListLogLogPlot[txalm[[All, {1, 3}]], PlotStyle -> Red],
    AxesLabel -> {"time\nspan", "area"}],
  Show[ListLogLogPlot[txal[[All, {2, 3}]]], ListLogLogPlot[txalm[[All, {2, 3}]],
     PlotStyle -> Red], AxesLabel -> {"map\nlength", "area"}]}]
{{Fit[Log[txal[[All, {1, 3}]]], {1, t}, t],
    Fit[Log[txalm[[All, {2, 3}]]], {1, t}, t]},
   {Fit[Log[Reverse /@ txal[[All, {1, 3}]]], {1, r}, r],
    Fit[Log[Reverse /@ txalm[[All, {2, 3}]]], {1, r}, r]}} // Transpose // TableForm
```

Out[306]=

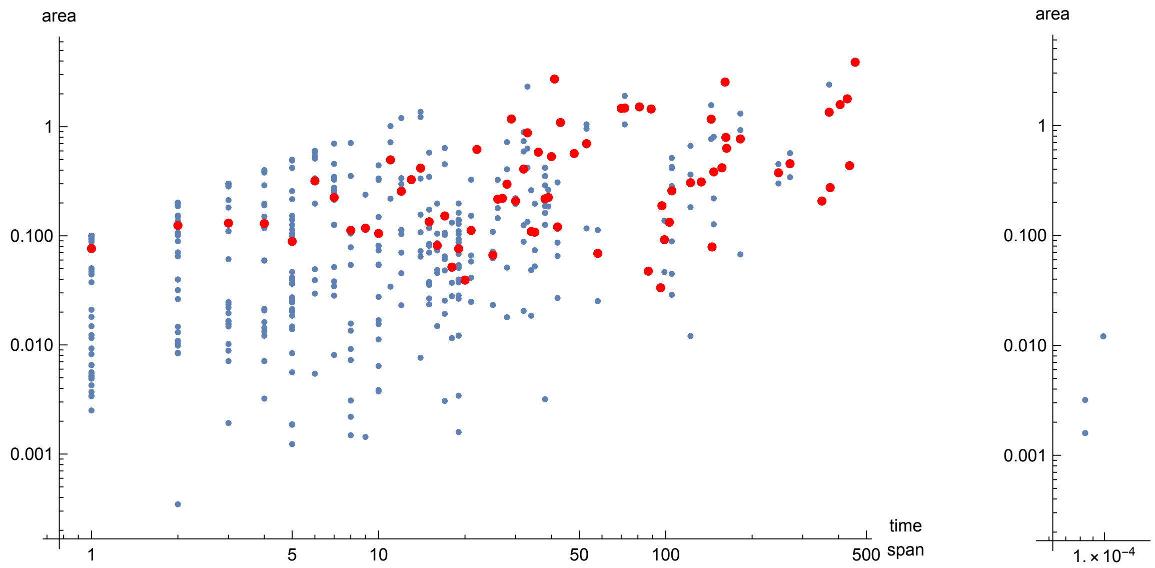

Out[307]//TableForm=

-3.17497 + 0.342694 t	2.91104 + 0.369998 r
0.124608 + 0.262843 t	-4.68366 + 0.347307 r

The left plot shows the distribution of areas; note the peaks at 0.1, 0.2 which correspond to branches that start at the tips and go back 1 or 2 generations before the first coalescence. The right plot shows the cumulative distribution of area. The total ancestry (map length*generations) is 105.3, on 488 branches; 44 branches with area>0.58 carry half of this, and 243 with area>0.1 carry 90% of the total ancestry.

In[359]:=
```
sa = Sort[txal[[All, 3]]]; ta = FoldList[Plus, 0, sa];
nb = Length[txal];
n50 = nb - findT[ta, Last[ta] / 2];
n90 = nb - findT[ta, Last[ta] / 10];
GraphicsRow[{Histogram[Log[txal[[All, 3]]], 40,
   Ticks -> {{{Log[0.001], "0.001"}, {Log[0.01], "0.01"}, {Log[0.1], "0.1"},
      {Log[1], "1"}}, {0, 50, 100}}, AxesLabel -> {"area", "#"}],
  Show[ListPlot[ta, PlotRange -> All], Plot[Last[ta] {1/2, 1/10}, {x, 0, 488}]]}]
{{"", "50%", "90%"}, {nb, n50, n90},
  {Last[ta], sa[[-n50]], sa[[-n90]]}} // TableForm
```

Out[361]=

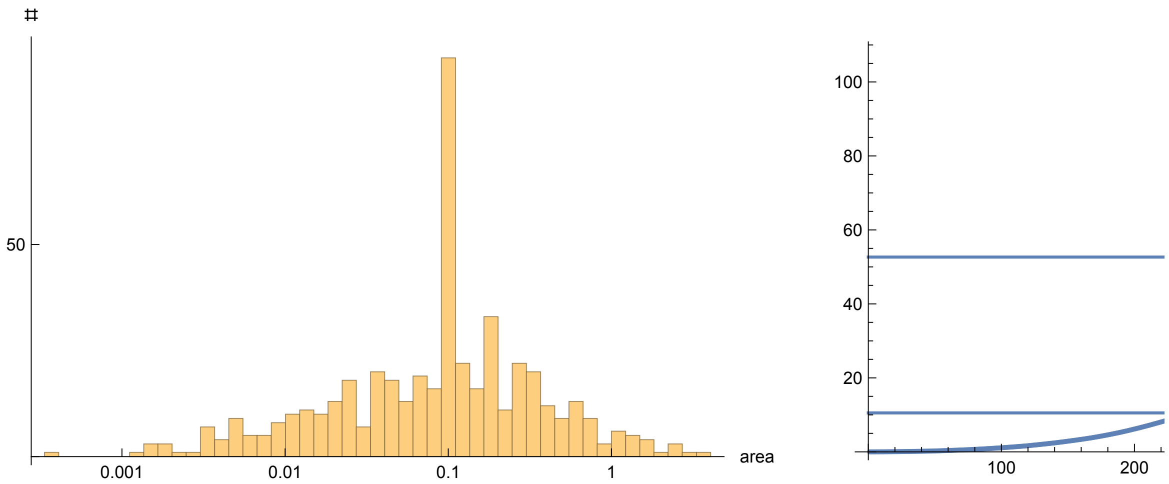

Out[362]//TableForm=

	50%	90%
488	44	243
105.298	0.578474	0.1

Histograms of time span (left) and map length (right). Blue: all 488 branches; yellow: the 243 branches that carry 90% of the ancestry; red: the 44 branches that carry 50% of the ancestry. The 44 most substantial branches (red) span a wide range, but tend to have longer timespans and span wider map lengths than for the branches as a whole.

In[398]:=
```
txal50 = Select[txal, #[[3]] > sa[[-n50]] &];
txal90 = Select[txal, #[[3]] > sa[[-n90]] &];
GraphicsRow[
 {Histogram[{Log[txal[[All, 1]]], Log[txal90[[All, 1]]], Log[txal50[[All, 1]]]},
   60, ChartStyle -> {Blue, Yellow, Red}, AxesLabel -> {"time\nspan", "#"}, Ticks ->
    {{{Log[1], "1"}, {Log[2], "2"}, {Log[3], "3"}, {Log[4], "4"}, {Log[5], "5"},
      {Log[10], "10"}, {Log[30], "30"}, {Log[100], "100"}}, {0, 50, 100}}],
  Histogram[{Log[txal[[All, 2]]], Log[txal90[[All, 2]]], Log[txal50[[All, 2]]]},
   60, ChartStyle -> {Blue, Yellow, Red},
   AxesLabel -> {"map\nlength", "#"}, Ticks -> {{{Log[0.001], "0.001"},
      {Log[0.01], "0.01"}, {Log[0.1], "0.1"}}, {0, 50, 100}}]}]
```

Out[399]=

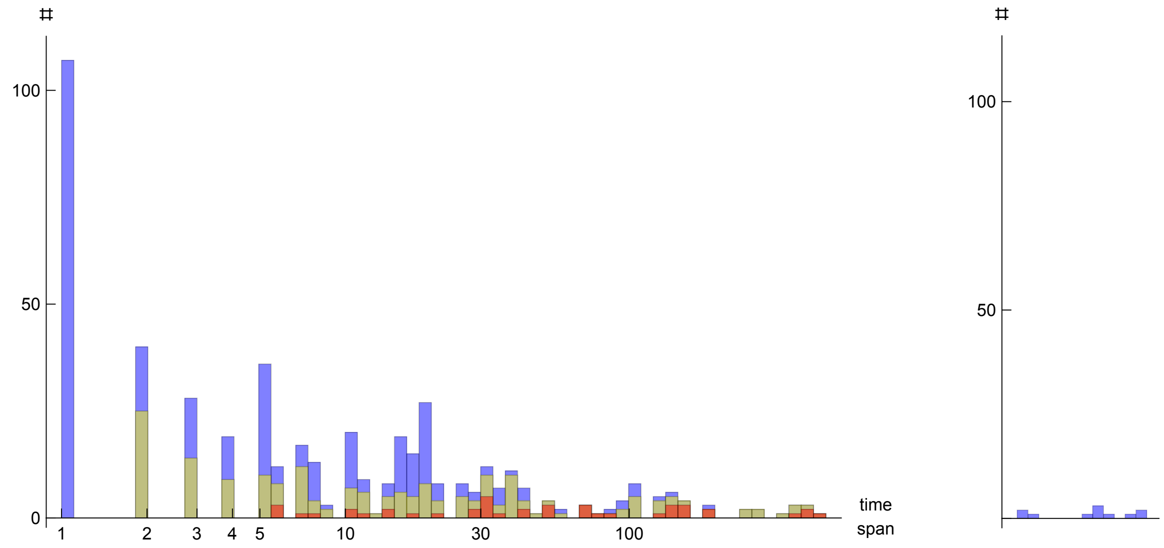

Table of branch properties

Note that all branches are on a single interval

In[2097]:=
```
tallyIntervalNumber[blf]
```

Out[2097]= {{1, 54}}

In[2071]:=
```
TableForm[
 Prepend[blf10, {"Clade", "time of origin", "map interval"}], TableDepth -> 2]
```

Out[2071]//TableForm=

Clade	time of origin	map interval
{1}	1	{{0, 0.1}}
{2}	1	{{0, 0.1}}
{3}	1	{{0, 0.1}}
{4}	1	{{0, 0.1}}
{5}	1	{{0, 0.1}}
{6}	1	{{0, 0.1}}
{7}	1	{{0, 0.1}}
{8}	1	{{0, 0.1}}
{9}	1	{{0, 0.1}}
{10}	1	{{0, 0.1}}
{1, 9}	3	{{0.00269484, 0.0216572}}
{3, 5}	4	{{0.0615051, 0.1}}
{3, 5}	12	{{0.015999, 0.0615051}}
{1, 4}	4	{{0, 0.00269484}}
{7, 10}	8	{{0, 0.1}}
{1, 4, 8}	8	{{0, 0.00269484}}
{4, 8}	8	{{0.00269484, 0.1}}
{6, 9}	10	{{0, 0.00215397}}
{2, 7, 10}	15	{{0.0140003, 0.0253309}}
{2, 6}	15	{{0.0253309, 0.1}}
{1, 4, 5, 8}	16	{{0, 0.000485901}}
{5, 6}	16	{{0.00215397, 0.015999}}
{3, 5, 6}	16	{{0.015999, 0.0253309}}
{3, 5, 6}	65	{{0.0140003, 0.015999}}
{2, 3, 5, 6}	16	{{0.0253309, 0.1}}
{2, 3, 5, 6}	65	{{0.00215397, 0.0140003}}
{2, 4, 7, 8, 10}	21	{{0.0189914, 0.0253309}}
{4, 7, 8, 10}	21	{{0.0253309, 0.0712151}}
{2, 3, 5, 6, 9}	28	{{0.0453142, 0.0965451}}
{2, 3, 5, 6, 9}	37	{{0.0446031, 0.0453142}}
{6, 7, 9, 10}	29	{{0, 0.00215397}}
{7, 9, 10}	29	{{0.00215397, 0.00269484}}
{1, 7, 9, 10}	29	{{0.00269484, 0.0140003}}
{1, 7, 9, 10}	131	{{0.0991522, 0.1}}
{1, 2, 7, 9, 10}	29	{{0.0140003, 0.0189914}}
{1, 2, 4, 7, 8, 9, 10}	29	{{0.0189914, 0.0216572}}
{1, 2, 4, 7, 8, 9, 10}	41	{{0.0140003, 0.0189914}}
{2, 4, 7, 8, 9, 10}	29	{{0.0216572, 0.0253309}}
{4, 7, 8, 9, 10}	29	{{0.0253309, 0.0446031}}
{1, 7, 10}	34	{{0.074802, 0.1}}
{1, 7, 10}	46	{{0.0712151, 0.074802}}
{2, 3}	37	{{0, 0.0140003}}
{1, 4, 7, 8, 9, 10}	41	{{0.00912131, 0.0140003}}
{2, 3, 4, 5, 6, 8, 9}	48	{{0.0745833, 0.0965451}}
{2, 3, 4, 5, 6, 8}	48	{{0.0965451, 0.1}}
{1, 2, 3, 4, 5, 8}	65	{{0, 0.000485901}}
{2, 3, 5}	65	{{0.000485901, 0.00215397}}
{1, 3, 5, 6}	65	{{0.0216572, 0.0253309}}
{1, 2, 3, 5, 6}	65	{{0.0253309, 0.0446031}}
{1, 2, 3, 5, 6, 9}	65	{{0.0446031, 0.0712151}}
{1, 2, 3, 5, 6, 7, 9, 10}	65	{{0.0712151, 0.0745833}}
{1, 2, 3, 5, 6, 7, 9, 10}	133	{{0.00269484, 0.00912131}}
{1, 2, 3, 4, 5, 6, 7, 8, 10}	116	{{0.0965451, 0.0991522}}
{2, 3, 5, 6, 7, 9, 10}	133	{{0.000485901, 0.00269484}}

Plotting the SNP on the most substantial branches

These are the SNP on the 9 longest branches (coded by colour):

In[2098]:=
```
colsF = {Black, Gray, Brown, Red, Orange, Green, Cyan, Magenta, Purple(*,Blue*)};
{big2full, colsF} // TableForm
```

Out[2099]//TableForm=

1	3	4	9	17	25	28	44	50
■	■	■	■	■	■	■	■	■

In this diagram, genome #1 is the bottom row, #10 the top. Thus, branch 1 leads down to to 1 (black), branch 14 leads down to {7, 10} (grey), and so on. These sets can be seen in the genealogies above.

In[2100]:=
```
ggBig = Graphics[Join[{PointSize[0.01]},
    Join @@ MapThread[{#2, plotSNP[#1, pop10full]} &, {big2full, colsF}],
    {LightBlue}, plotGenomes[10, 0.1]], AspectRatio -> 0.3]
```

Out[2100]=

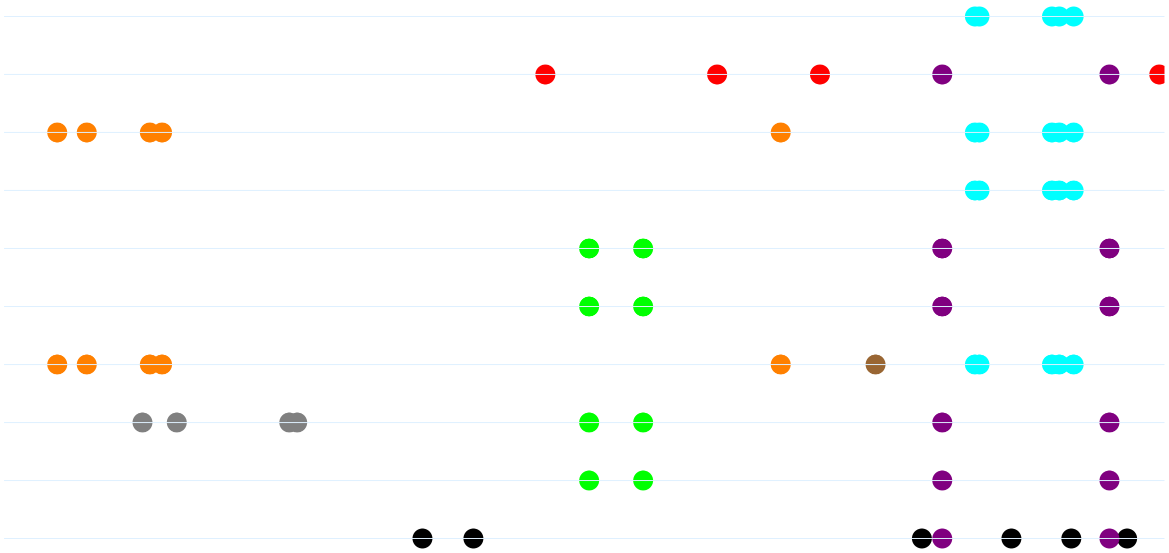

This uses the colour code above.   The blocks are shown in two different orders, to make clear that they often obscure each other:

In[2101]:=
```
gr = MapThread[Show[plotBranchFull[blf10[[#1]], #2, pl10, 0.1],
      Graphics[Prepend[Table[Disk[snp10full[[#, j, {2, 1}]], {0.001, 3}],
         {j, Length[snp10full[[#]]]}], Black]],
      PlotRange -> {{0, 0.1}, {0, 270}}, AspectRatio -> 1] &, {big2full, colsF}];
GraphicsRow[{Show[gr], Show[Reverse[gr]]}]
```

Out[2102]=

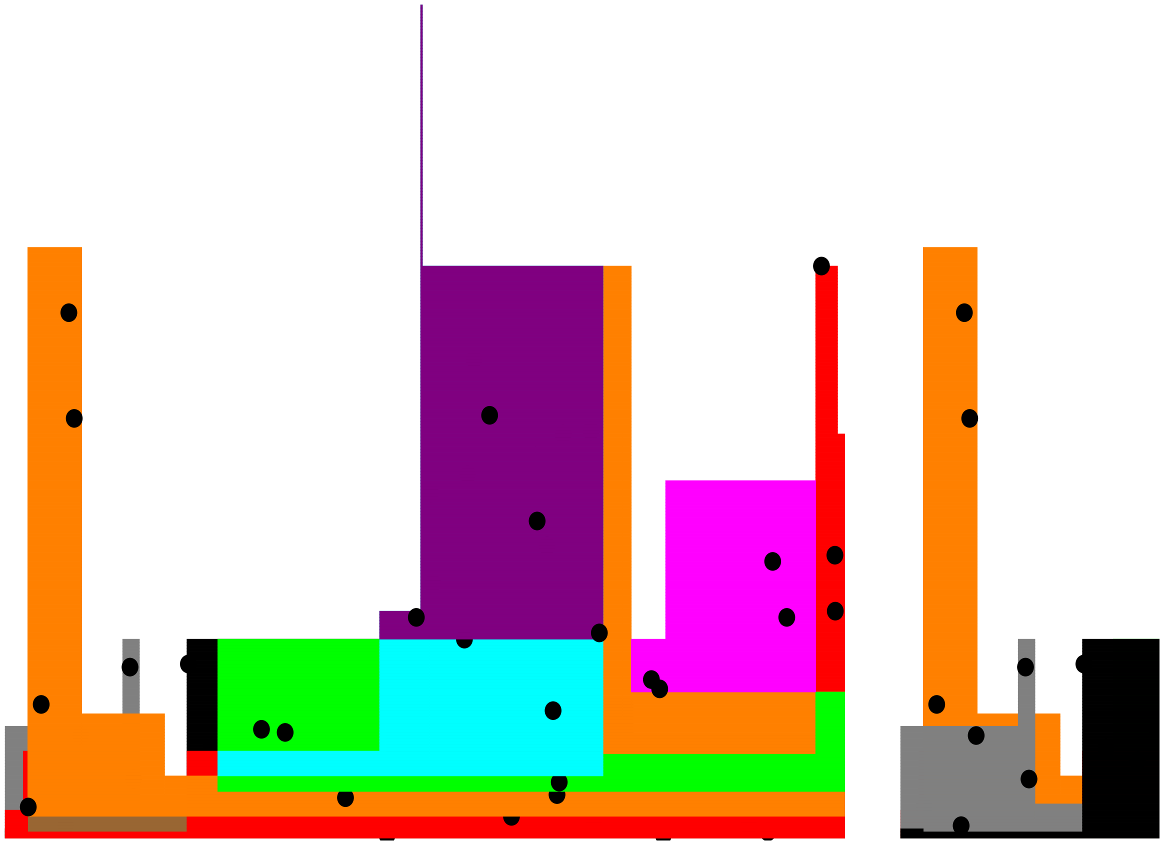

These blocks may be overlaid. This animation shows them separately, along with SNP superimposed:

In[2103]:=
```
ListAnimate[MapThread[
  Show[plotBranchFull[blf10[[#1]], #2, pl10, 0.1], Graphics[Prepend[Table[Disk[
        snp10full[[#, j, {2, 1}]], {0.001, 3}], {j, Length[snp10full[[#]]]}], White]],
    PlotRange -> {{0, 0.1}, {0, 270}}, AspectRatio -> 1] &, {big2full, colsF}]]
```

Out[2103]=

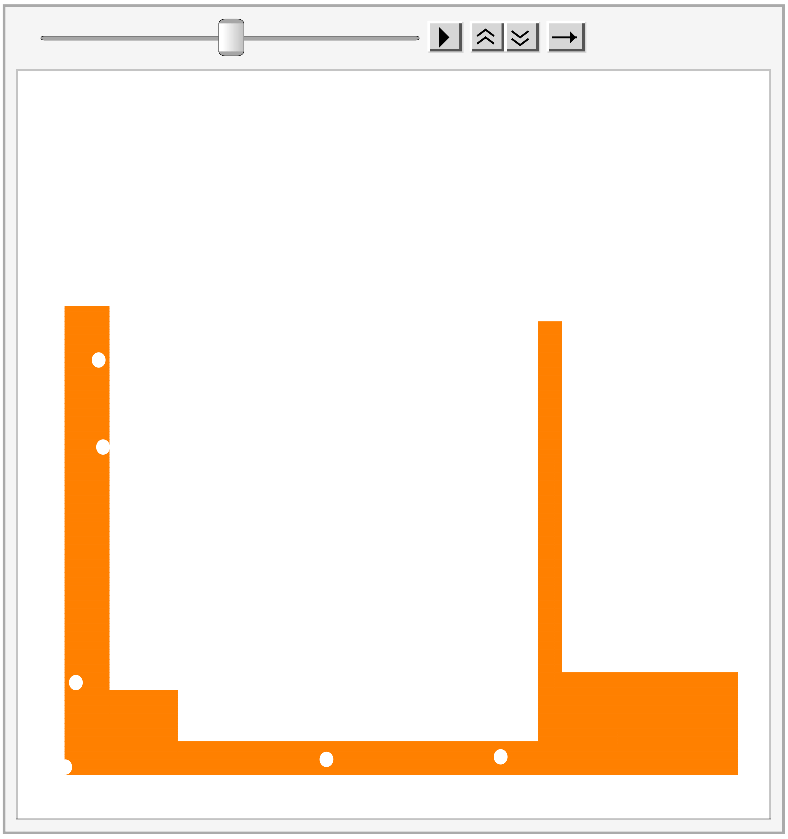

This shows all the 54 blocks:

In[2104]:=
```
cf = ColorData["DarkRainbow"];
gg[k_] := Show[plotBranchFull[blf10⟦k⟧, cf[(k - 1)/54], pl10, 0.1],
   Graphics[Prepend[Table[Disk[snp10full⟦k, j, {2, 1}⟧, {0.001, 3}],
      {j, Length[snp10full⟦k⟧]}], Black]], PlotRange -> {{0, 0.1}, {0, 270}},
   AspectRatio -> 1, PlotLabel -> "branch " ~~ ToString[k]];
ListAnimate[Table[gg[k], {k, 1, 54}]]
```

Out[2105]=

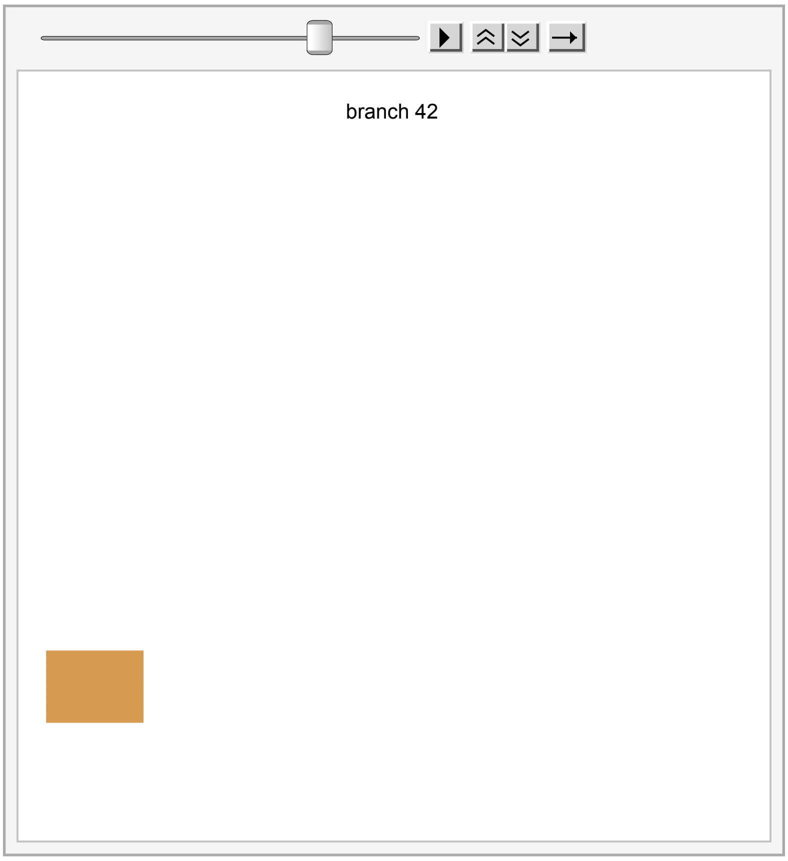

These are the data used in the block diagrams in the paper. The two versions only differ in the order with which blocks are overlaid.

Out[2102]=

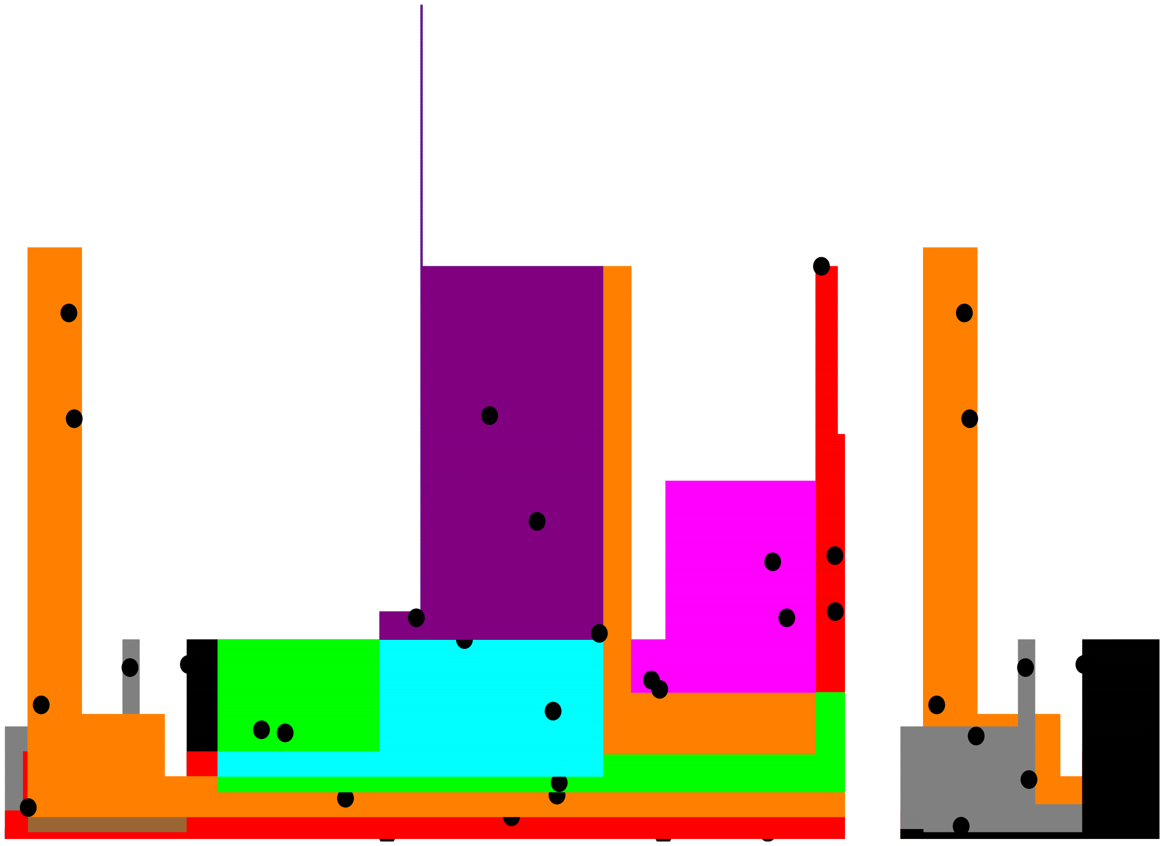

Selective sweep

Example with s=0.1, 2N=400, R=0.2, 20 genomes

Setting up the sweep

There is substantial variation between replicate sweeps, which arises mainly from random variation in the time at which exponential growth is established. The probability of fixation is ~2 s, and conditional on fixation, the sweep is established *as if* starting at an initial frequency $p_0$that is exponentially distributed, with mean 1/(2 s). This particular sweep has a rather long delay. The trajectory itself is spliced onto a very long run of 1’s, so that the simulation can continue back until all lineages have coalesced.

In[3093]:=
```
mrx = makeRepsFix[0.1, 400, 1, 1];
selB = Join[ConstantArray[1, 10^6], mrx[[1]]];
GraphicsRow[{Show[ListLinePlot[mrx[[1]]], PlotRange -> All],
  Show[ListLogPlot[mrx[[1]]], PlotRange -> All]}]
Length /@
 mrx
```

Out[3095]=

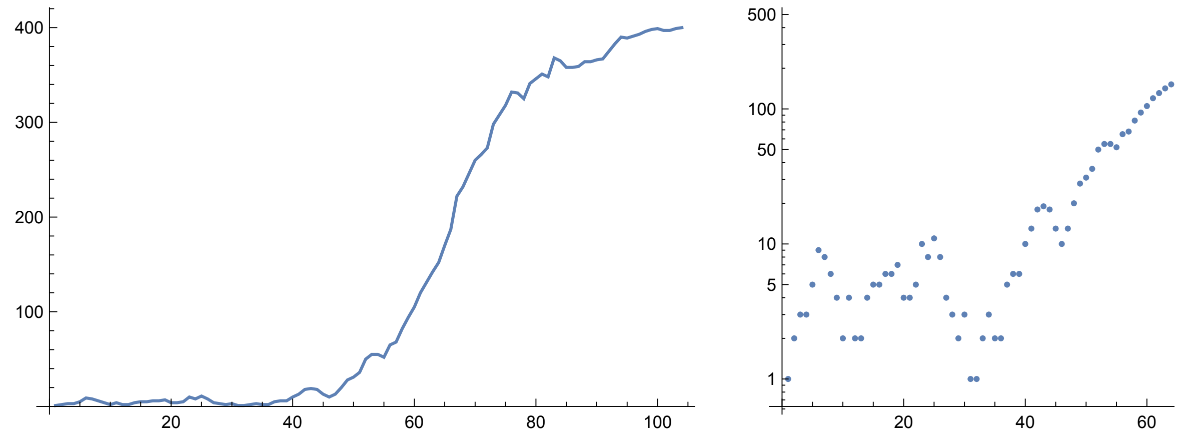

Out[3096]= {104}

Setting up ancestries and genealogies, and throwing down SNP: μ/r=2

We use the same sweep as above, as coded in selB; it takes 104 generations to fixation.

This iterates 20 lineages back through time, to the beginning of the sweep. R=0.1, $2N = 400$; $2NR = 40$. Complete coalescence takes 4272 generations in total.

In[768]:=
```
Timing[time = 0; sweepB20 = makeC[20, 0.2, 400, selB];
 Length[sweepB20]]
```

Out[768]= {39.6301, 4272}

This sets the ancestral genotype, prior to the mutation, to zero:

In[1060]:=
```
tMut = bounds[Reverse[selB]][[-1, 1]];
sweepB20 = Join[sweepB20[[1 ;; tMut - 1]],
   Replace[sweepB20[[tMut ;; -1]], {1, x__} :> {0, x}, {2}]];
```

This sets up a list of genealogies for each of the 428 intervals along the 20cM stretch of genome. We track yy, the interval it is working on. This is quite slow (and an inefficient algorithm, since it works independently on each genealogy).

```
ints20 = intervals[sweepB20, 0.2];
Timing[genB20 = (yy = #;
      makeGenealogyFull[sweepB20, yy, 0.2]) & /@ ints20;]
```

Out[1044]= {6394.2, Null}

This generates a set of genealogies that have an outgroup added, and also that specify the associated genotype at the selected locus. It takes some time to add the ancestries:

In[661]:=
```
genB20New =
  Table[addOutgroup[addGenotype[genB20[[j]], sweepB20, 20, ints20[[j]], 0.2],
    {10^5, {}, 0}], {j, Length[ints20]}];
```

This throws 937 SNP onto the 730 branches and 20 genomes (using addSNPFull). There are 4762 segregating alleles:

In[1100]:=
```
blB20 = branchListFull[sweepB20, 0.2];
snpB20 = makeSNP[posBlockFull[sweepB20, #, 0.2], 2] & /@ blB20;
popB20 = addSNP[blB20, snpB20];

{Length[snpB20], Total[Length /@ snpB20],
 Dimensions[popB20], Total[Length /@ popB20]}
```

Out[1103]=
```
{730, 937, {20}, 4762}
```

This saves the example. Note that there are two sets of genealogies: genB20 which includes only the sampled 20 genomes, and genB20New which includes the selected background and an out-group. blB20 lists branches only from the first set.

In[1062]:=
```
Save["Big sweeps s=0.1, 2N=400 R=0.2 20 genomes 2 Jan",
  {selB, sweepB20, genB20, genB20New, blB20, snpB20, popB20, ints20}];
```

In[620]:=
```
<< "Big sweeps s=0.1, 2N=400 R=0.2 20 genomes 2 Jan";
```

Description of the genealogies

This shows depth (top right) and mean pairwise divergence (lower left) for the genealogies. The lower right plot shows SNP patterns, comparing the # of segregating sites (black) with π (blue) along the genome. The classic theory works on average, but not necessarily in any one instance, even for a rather clear sweep..

In[707]:=
```
dgf = genFunction[DepthOfGenealogy, x, genB20, intervals[sweepB20, 0.2]];
pwf = genFunction[MeanPairwiseDivergence, x, genB20, intervals[sweepB20, 0.2]];
GraphicsGrid[{{ListLinePlot[Take[selB, -110]],
    LogPlot[dgf, {x, 0, 0.2}, PlotRange -> {{0, 0.2}, {40, 4000}}]},
   {LogPlot[pwf, {x, 0, 0.2}, PlotRange -> {{0, 0.2}, {40, 4000}}],
    Show[ListLogPlot[Transpose[{Range[δ/2, 0.2 - δ/2, δ], nSites /@ snpW}],
      PlotStyle -> Black, Joined -> True, PlotRange -> {{0, 0.2}, {0.4, 50}}],
     ListLogPlot[Transpose[{Range[δ/2, 0.2 - δ/2, δ], piSNP /@ snpW}],
      Joined -> True]]}}]
```

Out[709]=

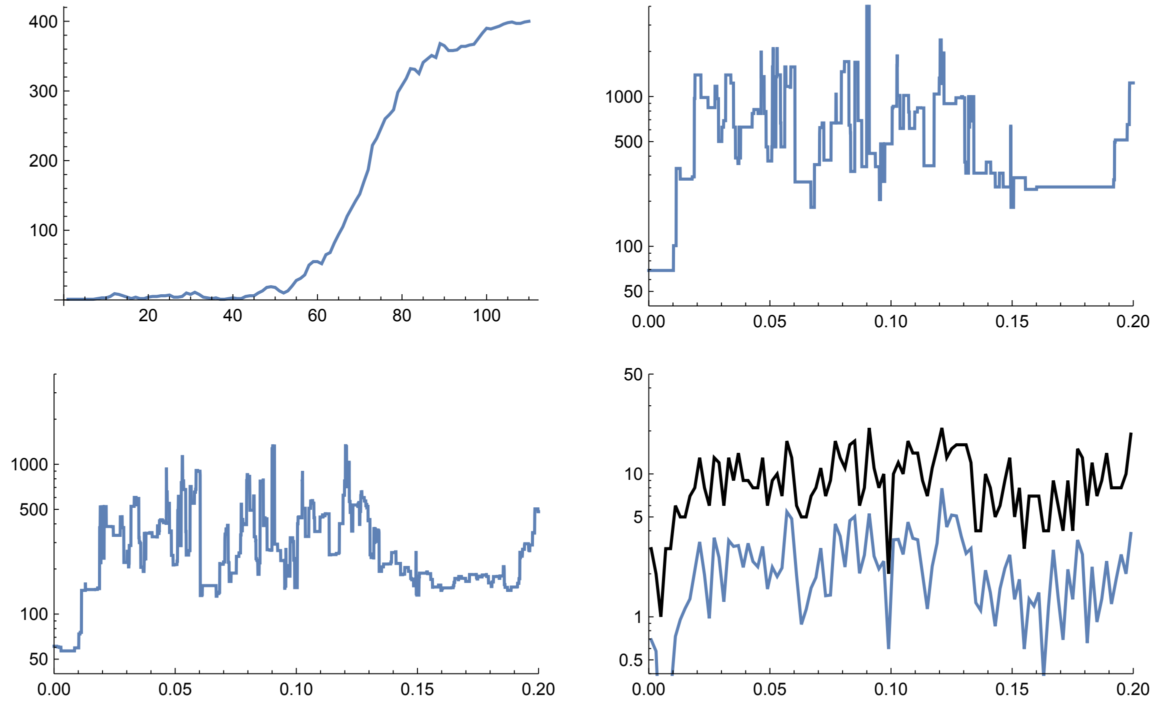

The left plot is a histogram of the block areas, on a $\log_{10}$ scale - centred on about 0.1; the right two plots give the distribution of # SNP (log and normal scale). Most branches have no SNP; 604/937 SNP are on 51 branches with at least 5, 438 are on the 25 branches with at least 10, and 209 are on the 8 branches with at least 20.

In[1220]:=
```
GraphicsRow[{Histogram[Log[10, branchAreaFull[sweepB20, 0.2]]],
  Histogram[Log[10, Length /@ snpB20], 20], Histogram[Length /@ snpB20, 20]}]
```

Out[1220]=

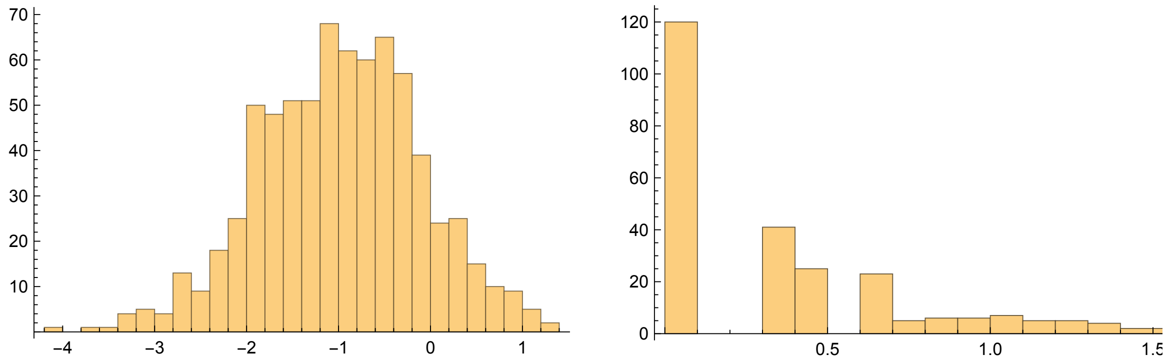

Genealogies along the genome

This shows how the genealogy along the genome, on a log scale. The genomic interval is shown below each plot, and the numbers label the branches. Red indicates branches that originate on the new (fitter) genetic background.

Out[821]=

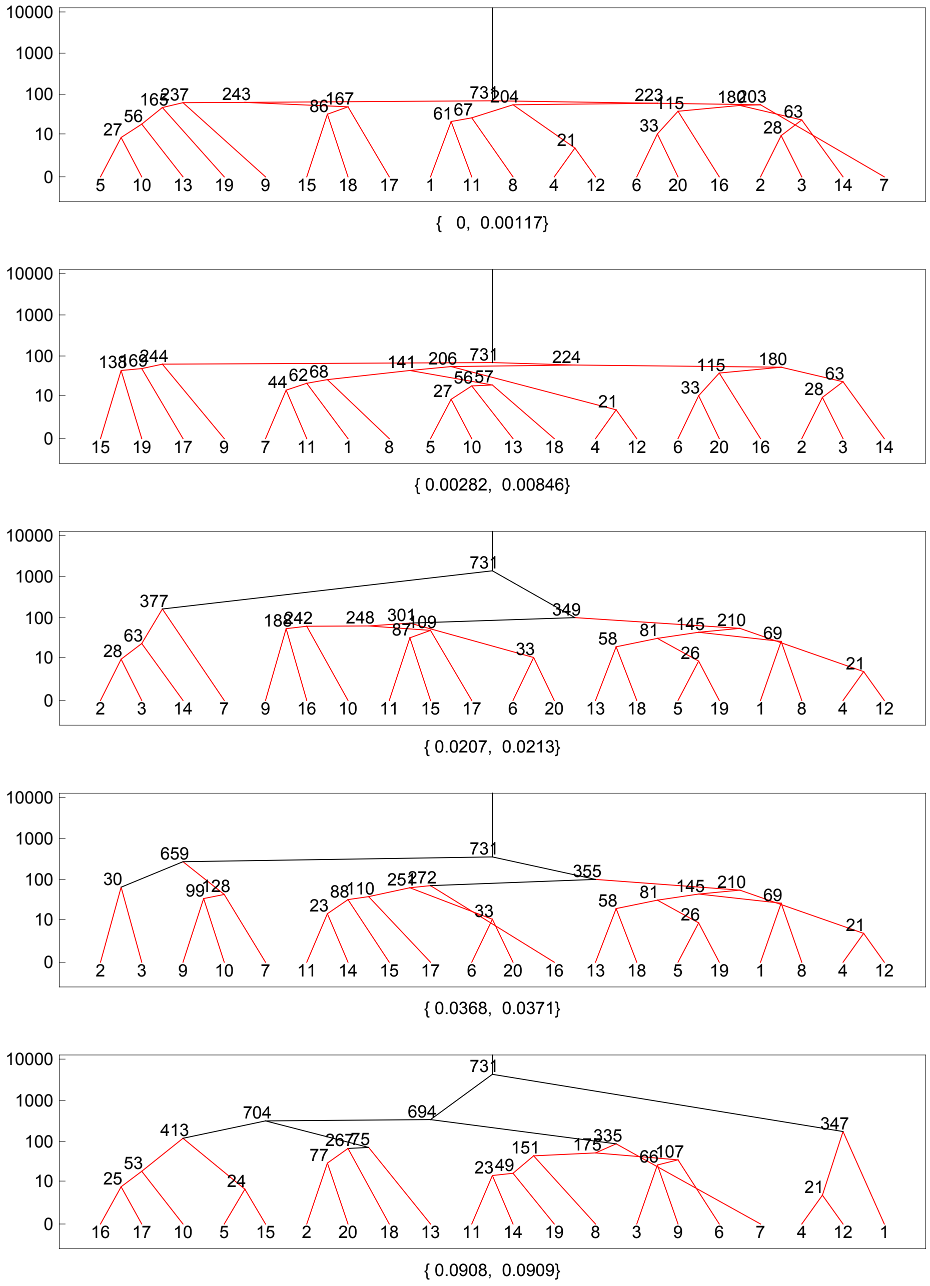

Below, doing the same, colouring in the branches properly (by showing where the recombination onto a different background happens), and marking times when $p = \frac{1}{400}$, 0.1, 0.5, 0.9 (at {22,38,53,110 generations; dashed lines}). Note that coalescence can only occur within the same background (i.e. black with black or red with red). In the recent past, recombinations may occur within the derived (red) background; further back, when the fitter allele is still rare, recombination will almost certainly be onto the ancestral background (red→black), rather than in the opposite

direction. Recombination out is likely when *p* is below 0.5, and must happen before the origin of the mutation if coalescence is to be avoided. Up to map position ~0.01, there is some rearrangement amongst recent genealogies, but all lineages coalesce in a MRCA associated with the new mutation (marked 731 in the top left and top middle panels). The first recombination splits the genealogy into clades of size {4,16}; the number of deep branches (that trace back >110 generations, deeper than the top dotted line) for the 9 trees shown below is {1,1,2,2,3,3,5,6,6}. Note that under the standard coalescent, the expected time for a large sammple to coalesce down to 6 lineages is 120 generations - so we expect that large samples will already be substantially thinned before the sweep is reached. By map position 0.0129 (top right), we have had 5 recombinations out (and one back) at {{38,45},47,65,90}. At the end of the genome (map position 0.2; bottom right) there have been about 7 recombination events. One can accurately calculate the expectations for these numbers using the standard coalescent.

Out[1167]=

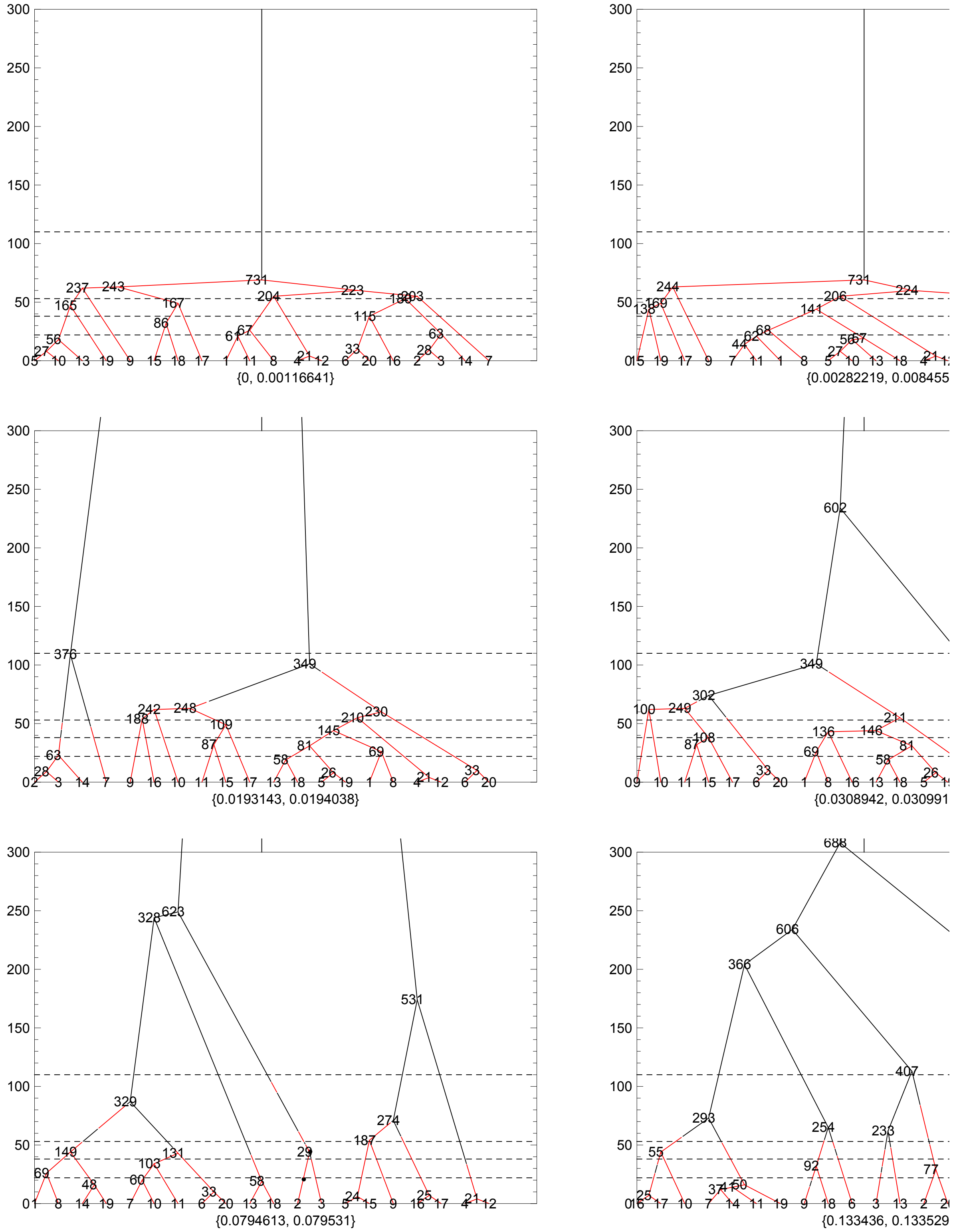

Out[1168]= {{0, 0.00116641}, {0.00282219, 0.00845516}, {0.0129117, 0.0129652}, {0.0193143, 0.0194038}, {0.0308942, 0.0309919}, {0.0463784, 0.0464419}, {0.0794613, 0.079531}, {0.133436, 0.133529}, {0.197523, 0.198033}}

General considerations

The following sections try various ways of identifying sets of “interesting” branches.

A first step in identifying a selective sweeps is to find branches that are associated with that sweep. The simplest case is where a single mutation sweeps through an extremely large population, which is sampled immediately after the sweep. Then, lineages either coalesce soon after the mutation arises, when it is still in small numbers, or they recombine out and coalesce much further back. However, a large sample will coalesce down to k lineages in $2\,N_e/k$ generations, where N_e is the haploid effective population size. Thus, there can be appreciable coalescence more recently than the sweep, which would happen even in its absence. This is seen in the example here, where N_e is only 400; at the focal locus we have only 10 lineages out of the original 20, at the time when the sweeping allele is at 50%.

Branches that are associated with the selected locus

Identifying branches with coalescences that occurred at or more recently than the sweep seems problematic. Here, we find 18 branches that cover the selected locus; 8 of these are not nested within others. The table shows the genomic interval; the time of the coalescence; and the set of descendant branches. Note that this list includes sets of descendants that are nested within other sets for some of the genome; however, we list them separately, because they are not nested over their whole extent. For example, at the selected locus, {5,10} coalesce first, at generation 8, and then, at generation 18 back from the present, {5,10,13} coalesce. However, the branch {5,10} extends out to 1.68cM, whereas {5.10.13} extends back only out to 0.846cM. Thus, {5,10} is only nested within {5,10,13} over part of its extent.

In[1166]:=
```
blB20sel = DeleteCases[Cases[blB20, {_, _, {{0, _}, ___}}], {{_}, _, _}];
blB20notNested = Select[blB20sel, Not[nestedQ[#, blB20sel]] &];
TableForm[Sort[Reverse /@ blB20notNested], TableDepth -> 2]
Length /@ {blB20sel, blB20notNested}
```

Out[1168]//TableForm=

{{0, 0.00282219}}	60	{1, 2, 3, 4, 6, 7, 8, 11, 12, 14, 16, 20}
{{0, 0.00282219}}	63	{5, 9, 10, 13, 15, 17, 18, 19}
{{0, 0.00845516}}	18	{5, 10, 13}
{{0, 0.0107685}}	53	{2, 3, 6, 14, 16, 20}
{{0, 0.0168376}}	8	{5, 10}
{{0, 0.0278147}}	23	{2, 3, 14}
{{0, 0.0905804}}	10	{6, 20}
{{0, 0.163959}}	4	{4, 12}

Out[1169]= {18, 8}

For the 8 branches, these are the # of descendants; times of origin; extent; # SNP; and areas. Only three carry more than 1 SNP, and those three make up all but 3% of the area. However, only the last three branches are actually generated by the sweep - that is, coalesce within the favourable background whilst it is still rare:

In[1231]:=
```
xx = Flatten[Position[blB20, #] & /@ blB20notNested];
TableForm[{Length /@ blB20notNested[[All, 1]],
  blB20notNested[[All, 2]], blB20notNested[[All, 3]], Length /@ snpB20⟦xx⟧,
  branchAreaFull[sweepB20, 0.2][[xx]]}, TableDepth -> 2]
```

Out[1232]//TableForm=

2	2	2	3	3
4	8	10	18	23
{{0, 0.163959}}	{{0, 0.0168376}}	{{0, 0.0905804}}	{{0, 0.00845516}}	{{0,
35	1	10	1	4
18.6594	0.537201	2.88222	0.0638996	2.52

This shows the 8 non-nested branches that are associated with the focal genealogy. Only four blocks are really visible.   The light pink area at left is where the whole genealogy coalesces in the sweep

In[1182]:=
```
ppb = Flatten[Position[blB20, #] & /@ blB20notNested];
cf = ColorData["DarkRainbow"];
cols = cf /@ Range[1, 0, -1/(Length[ppb] - 1)];
nb = Length[snpB2];
gb[b_, c_] := Show[plotBlockFull[blB20[[b]], Lighter[c, 0.7], sweepB20, 0.2],
   Graphics[{c, PointSize[0.007], plotSNP[b, popB20]}],
   PlotRange -> {{0, 0.21}, {0, 21}}];
cr = coalescedRegions[sweepB20[[70]], 20];
Show[Graphics[
  plotSNP[Complement[Range[nb], ppb], ConstantArray[Gray, nb], 0.004, popB20]],
 MapThread[gb, {ppb, cols}],
 Graphics[
  Join[{Opacity[0.3, Red]}, Rectangle[{#[[1]], 0}, {#[[2]], 20}] & /@ cr]],
 AspectRatio -> 0.2, Frame -> True]
```

Out[1186]=

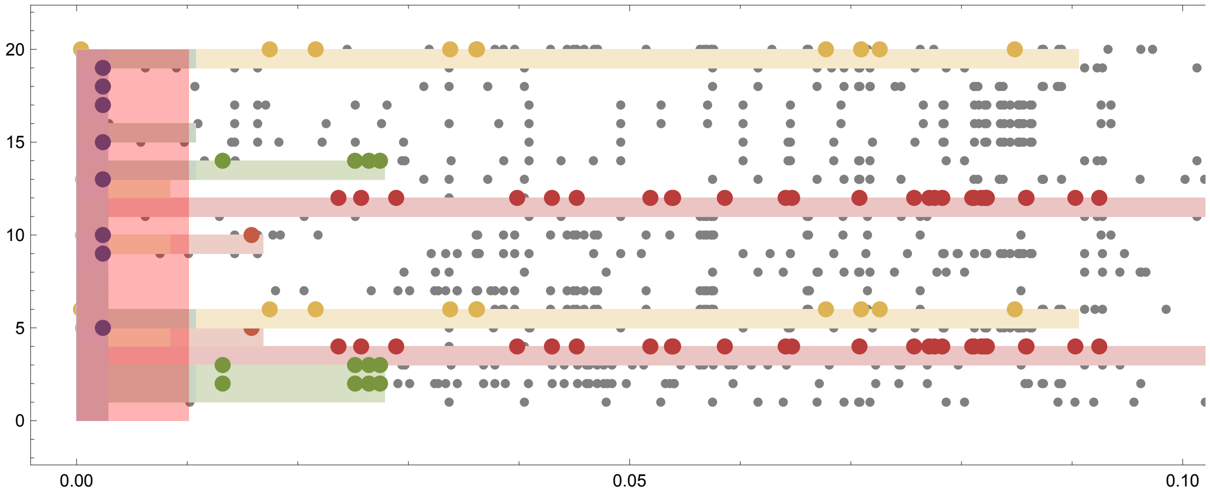

Showing the largest branches: 30 non-tip branches, area>2

These are the # of blocks and their areas, for different threshold areas. 7 of the branches give 22% of the area, whilst 22 give 44%. I choose a threshold area of 2, and restrict to non-tips which gives 33% of the area.

In[587]:=
```
ba = branchAreaFull[sweepB20, 0.2];
ss[th_] := (bas = Select[ba, # ≥ th &];
   {th, Length[bas], Total[bas], Total[bas] / Total[ba]});
TableForm[ss /@ {0.5, 1, 2, 5, 10, 15}]
```

Out[589]//TableForm=

0.5	149	381.959	0.847243
1	90	340.934	0.756243
2	49	281.021	0.623347
5	22	199.645	0.442843
10	7	98.4184	0.218307
15	2	36.7701	0.0815617

In[590]:=
```
big = Select[Range[Length[blB20]], (ba[[#]] >= 2) ∧ (blB20[[#, 2]] > 1) &];
{Length[big], Total[ba[[big]]] / Total[ba], Total[Length /@ snpB20[[big]]] / Total[Length /@ snpB20] // N}
```

Out[591]= {32, 0.329255, 0.308431}

Identifying branches with coalescences at or more recently than the sweep seems problematic. Here, I find 32 non-tip branches that cover 33% of the area and 31% of SNP; two of these are nested, and so dropped, leaving 30 branches.

In[592]:=
```
blB20notNested = Select[blB20[[big]], Not[nestedQ[#, blB20[[big]]]] &];
TableForm[Sort[Reverse /@ blB20notNested], TableDepth -> 2]
Length /@ {blB20[[big]], blB20notNested}
```

Out[593]//TableForm=

{{0, 0.0278147}}	23
{{0, 0.0905804}}	10
{{0, 0.163959}}	4
{{0.00845516, 0.0826403}}	19
{{0.0129117, 0.0203284}}	109
{{0.0129117, 0.0330864}}	101
{{0.0207029, 0.0306668}}	164
{{0.0352883, 0.049621}}	65
{{0.0377105, 0.0699155}}	44
{{0.0377105, 0.2}}	6
{{0.040501, 0.0509682}}	101
{{0.040501, 0.0555959}}	43
{{0.0422193, 0.185441}}	7
{{0.0555959, 0.0867949}}	43
{{0.0684998, 0.0836397}}	44
{{0.0720953, 0.0862156}}	71
{{0.080859, 0.0823478}}	316
{{0.0899643, 0.0922881}}	340
{{0.0935484, 0.122784}}	43
{{0.100693, 0.105872}}	150
{{0.1016, 0.105872}}	174
{{0.10589, 0.122784}}	73
{{0.106769, 0.111089}}	175
{{0.117552, 0.126048}}	144
{{0.122784, 0.133868}}	73
{{0.126275, 0.13198}}	86
{{0.130813, 0.163959}}	10
{{0.138979, 0.163959}}	32
{{0.180743, 0.195878}}	14
{{0.00116641, 0.0129117}, {0.158675, 0.180407}}	14
{{0.0579233, 0.0602473}, {0.0610882, 0.0672732}}	109
{{0.0823478, 0.0840355}, {0.0868214, 0.0894669}, {0.0899643, 0.0922881}}	174

Out[594]= {32, 32}

For the 30 branches, these are the # of descendants; times of origin; extent; # SNP; and areas. One branch(#347) is disjunct. Not many are generated by the sweep, or associated with it, in the sense that only 3 begin at zero, and only 9 originate between 40 and 80 generations back (which is when the sweep is inducing most coalescence)

In[597]:=
```
ppb = Flatten[Position[blB20, #] & /@ blB20notNested];
cols = ColorData["DarkRainbow"] /@ Range[1, 0, -1/(Length[ppb] - 1)];
TableForm[Prepend[Transpose[{ppb, cols, Length /@ blB20notNested[[All, 1]],
    blB20notNested[[All, 2]], Length /@ snpB20[[ppb]],
    branchAreaFull[sweepB20, 0.2][[ppb]], blB20notNested[[All, 3]]}],
  {"index", "colour", "# desc", "t", "# SNP", "area", "{r0,r1}"}], TableDepth -> 2]
```

Out[598]//TableForm=

index	colour	# desc	t	# SNP	area	$\{r_0,r_1\}$
21		2	4	35	18.6594	{{0, 0.163959}}
24		2	6	20	11.7729	{{0.0377105, 0.2}}
25		2	7	17	6.41979	{{0.0422193, 0.185441}}
29		2	44	3	3.85298	{{0.0684998, 0.0836397}}
30		2	65	10	5.08574	{{0.0352883, 0.049621}}
33		2	10	10	2.88222	{{0, 0.0905804}}
35		3	10	3	2.10807	{{0.130813, 0.163959}}
40		2	14	10	2.81302	{{0.180743, 0.195878}}
44		2	14	5	2.73779	{{0.00116641, 0.0129117}, {0
58		2	19	5	2.81091	{{0.00845516, 0.0826403}}
63		3	23	4	2.52994	{{0, 0.0278147}}
93		5	32	7	2.72372	{{0.138979, 0.163959}}
130		4	43	8	6.99008	{{0.040501, 0.0555959}}
131		5	43	10	4.0976	{{0.0555959, 0.0867949}}
132		3	43	7	2.39647	{{0.0935484, 0.122784}}
147		5	44	4	2.0393	{{0.0377105, 0.0699155}}
274		5	71	13	3.86921	{{0.0720953, 0.0862156}}
292		6	73	7	4.93033	{{0.10589, 0.122784}}
293		7	73	2	2.33088	{{0.122784, 0.133868}}
326		3	86	5	2.28132	{{0.126275, 0.13198}}
347		3	174	8	5.69969	{{0.0823478, 0.0840355}, {0.
349		16	101	23	13.2438	{{0.0129117, 0.0330864}}
358		14	101	12	5.05503	{{0.040501, 0.0509682}}
376		4	109	4	2.88023	{{0.0129117, 0.0203284}}
377		4	164	12	7.41051	{{0.0207029, 0.0306668}}
379		11	109	5	3.09476	{{0.0579233, 0.0602473}, {0.
465		6	144	7	2.56487	{{0.117552, 0.126048}}
492		14	150	8	3.78986	{{0.100693, 0.105872}}
534		6	174	8	3.04678	{{0.1016, 0.105872}}
544		13	175	5	2.09885	{{0.106769, 0.111089}}
680		18	316	2	2.07546	{{0.080859, 0.0823478}}
694		17	340	10	4.14525	{{0.0899643, 0.0922881}}

This shows the 30 non-nested, non-tip branches that are associated with the focal genealogy. The light pink rectangle at left is where the whole genealogy coalesces in the sweep. The substantial branches seem to be concentrated near the sweep, even though SNP diversity is high all along the genome, except for the leftmost 1cM

In[1299]:=
```
ppb = Flatten[Position[blB20, #] & /@ blB20notNested];
cf = ColorData["DarkRainbow"];
cols = cf /@ Range[1, 0, -1/(Length[ppb] - 1)];
nb = Length[snpB2];
gb[b_, c_] := Show[plotBlockFull[blB20[[b]], Lighter[c, 0.7], sweepB20, 0.2],
    Graphics[{c, PointSize[0.005], plotSNP[b, popB20]}],
    PlotRange -> {{0, 0.21}, {0, 21}}];
cr = coalescedRegions[sweepB20[[70]], 20];
Show[Graphics[
   plotSNP[Complement[Range[nb], ppb], ConstantArray[Gray, nb], 0.003, popB20]],
  MapThread[gb, {ppb, cols}],
  Graphics[
   Join[{Opacity[0.3, Red]}, Rectangle[{#[[1]], 0}, {#[[2]], 20}] & /@ cr]],
  AspectRatio -> 0.2, Frame -> True]
```

Out[1303]=

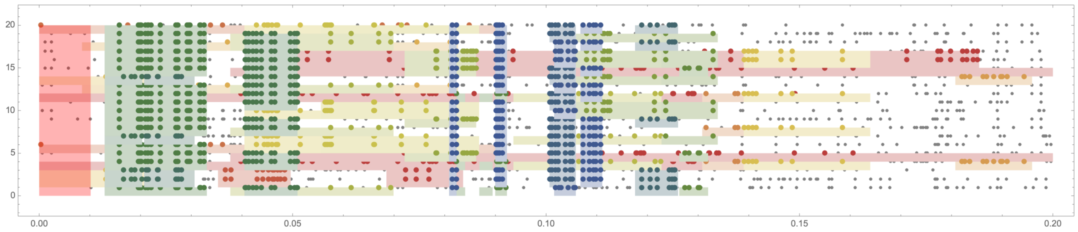

This shows genealogies at 0, 0.01, 0.02, 0.05, 0.20, on two different vertical scales. The first “escape” is just beyond 1cM, but the genealogy still retains the effects of the sweep out to 5cM or so:

```
In[1325]:= hh[j_, ym_] := Show[PlotGenealogy[genB20[[j]], NodeFunction -> plotCoords[1]],
               PlotRange -> {{-1, 20}, {0, ym}}, Frame -> True,
               FrameTicks -> {None, Automatic}, AspectRatio -> 0.7];
           ii = {1, 6, 22, 87, -1};
           GraphicsGrid[{hh[#, 150] & /@ ii, hh[#, 800] & /@ ii}]
           ints20[[ii]]
```

Out[1327]=

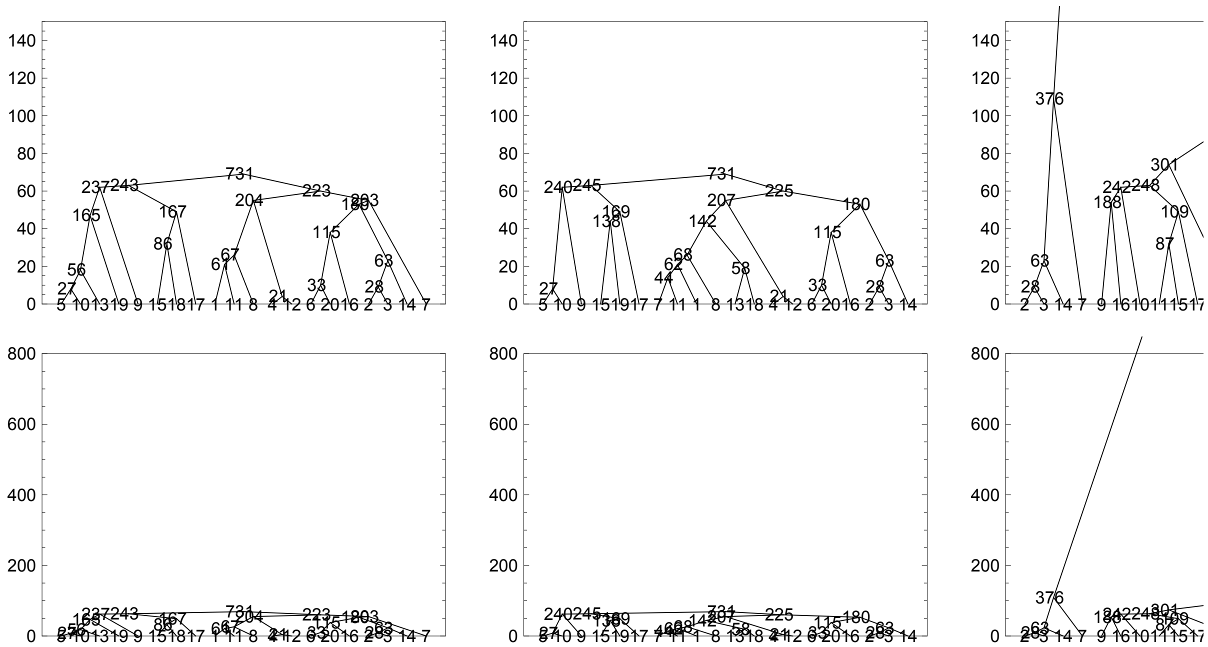


```
Out[1328]= {{0, 0.00116641}, {0.00845516, 0.0101709},
            {0.0199083, 0.0203284}, {0.049621, 0.0504018}, {0.199885, 0.2}}
```

These are the 30 branches; the vertical scale is truncated at 800:

In[1337]:=
```
gr = MapThread[
   Show[plotBranchFull[blB20[[#1]], #2, sweepB20, 0.2], Graphics[Prepend[Table[
        Disk[snpB20[[#, j, {2, 1}]], {0.001, 3}], {j, Length[snpB20[[#]]]}], Black]],
     PlotRange -> {{0, 0.2}, {0, 800}}, AspectRatio -> 1] &, {ppb, cols}];
GraphicsRow[{Show[gr], Show[Reverse[gr]]}]
```

Out[1338]=

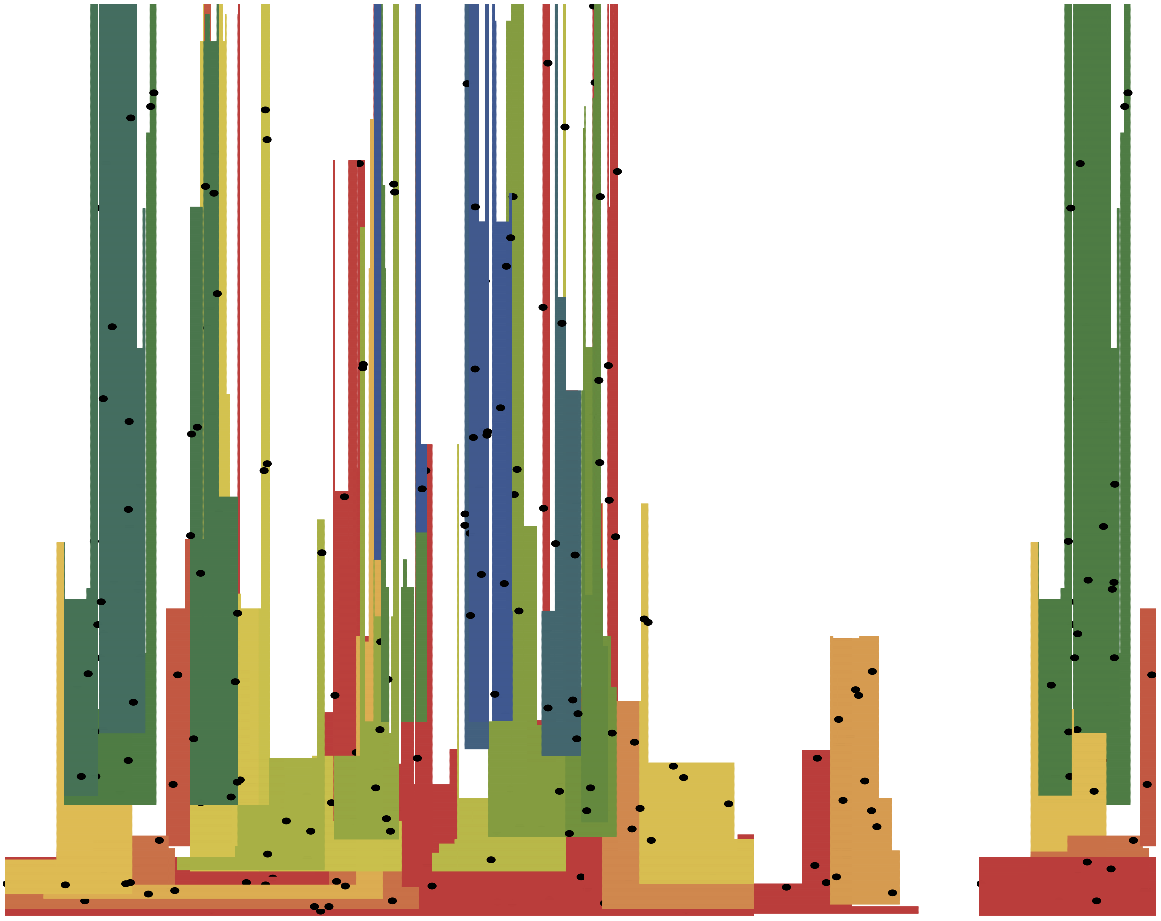

This shows all 30 of the “substantial” blocks

In[1343]:=
```
cf = ColorData["DarkRainbow"];
gg[k_] := Show[plotBranchFull[blB20⟦ppb[[k]]⟧, cf[(k - 1)/Length[ppb]], sweepB20, 0.2],
  Graphics[Prepend[Table[Disk[snpB20⟦ppb[[k]], j, {2, 1}⟧, {0.001, 3}],
     {j, Length[snpB20⟦ppb[[k]]⟧]}], Black]], PlotRange -> {{0, 0.2}, {0, 800}},
  AspectRatio -> 1, PlotLabel -> "branch " ~~ ToString[ppb[[k]]]];
ListAnimate[Table[gg[k], {k, 1, Length[ppb]}]]
```

Out[1344]=

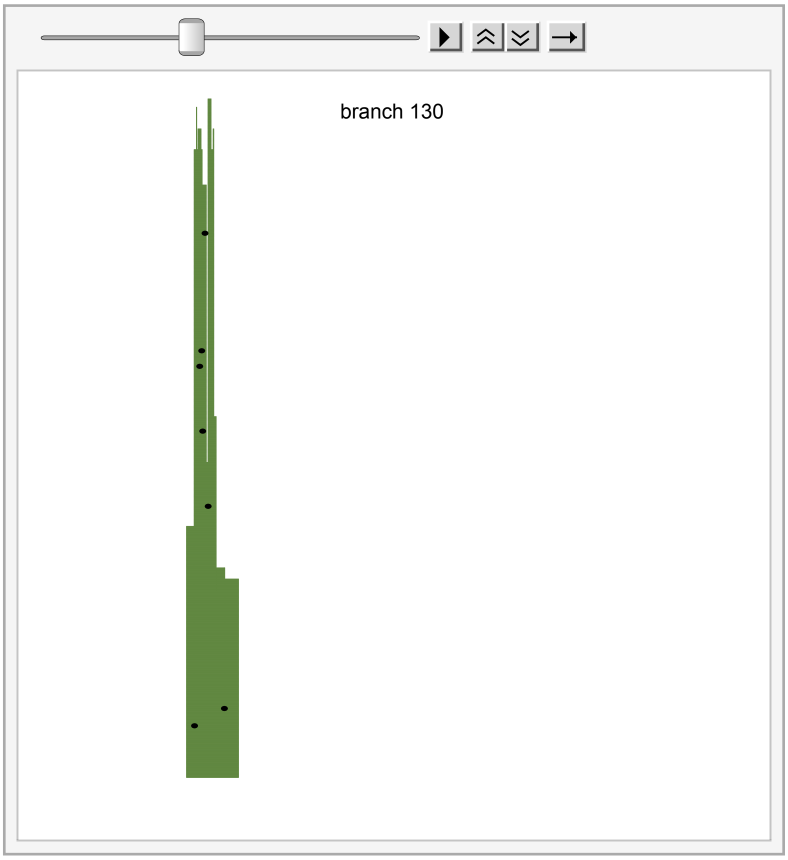

Showing the largest branches: 49 branches, area>2

With a threshold area of 2, including tip branches, we include 62% of the area and 61% of SNP:

In[601]:=
```
ba = branchAreaFull[sweepB20, 0.2];
big = Select[Range[Length[blB20]], (ba[[#]] >= 2) &];
{Length[big], Total[ba[[big]]] / Total[ba], Total[Length /@ snpB20[[big]]] / Total[Length /@ snpB20] // N}
```

Out[603]= {49, 0.623347, 0.614728}

None of these are nested. This sorts by left edge of the branch, and gives the extent, the time of origin, the descendants, and the area:

In[611]:=
```
blB20notNested = Select[blB20[[big]], Not[nestedQ[#, blB20[[big]]]] &];
tt = Sort[Reverse /@ blB20notNested];
TableForm[Transpose[{tt[[All, 1]], tt[[All, 2]], tt[[All, 3]], ba[[big]]}],
 TableDepth -> 2]
Length /@ {blB20[[big]], blB20notNested}
```

Out[613]//TableForm=

{{0, 0.0278147}}	23
{{0, 0.0905804}}	10
{{0, 0.163959}}	4
{{0, 0.2}}	1
{{0, 0.2}}	1
{{0, 0.2}}	1
{{0, 0.2}}	1
{{0, 0.2}}	1
{{0, 0.2}}	1
{{0, 0.2}}	1
{{0, 0.2}}	1
{{0, 0.2}}	1
{{0, 0.2}}	1
{{0, 0.2}}	1
{{0, 0.2}}	1
{{0, 0.2}}	1
{{0, 0.2}}	1
{{0, 0.2}}	1
{{0, 0.2}}	1
{{0, 0.2}}	1
{{0.00845516, 0.0826403}}	19
{{0.0129117, 0.0203284}}	109
{{0.0129117, 0.0330864}}	101
{{0.0207029, 0.0306668}}	164
{{0.0352883, 0.049621}}	65
{{0.0377105, 0.0699155}}	44
{{0.0377105, 0.2}}	6
{{0.040501, 0.0509682}}	101
{{0.040501, 0.0555959}}	43
{{0.0422193, 0.185441}}	7
{{0.0555959, 0.0867949}}	43
{{0.0684998, 0.0836397}}	44
{{0.0720953, 0.0862156}}	71
{{0.080859, 0.0823478}}	316
{{0.0899643, 0.0922881}}	340
{{0.0935484, 0.122784}}	43
{{0.100693, 0.105872}}	150
{{0.1016, 0.105872}}	174
{{0.10589, 0.122784}}	73
{{0.106769, 0.111089}}	175
{{0.117552, 0.126048}}	144
{{0.122784, 0.133868}}	73
{{0.126275, 0.13198}}	86
{{0.130813, 0.163959}}	10
{{0.138979, 0.163959}}	32
{{0.180743, 0.195878}}	14
{{0.00116641, 0.0129117}, {0.158675, 0.180407}}	14
{{0.0579233, 0.0602473}, {0.0610882, 0.0672732}}	109
{{0.0823478, 0.0840355}, {0.0868214, 0.0894669}, {0.0899643, 0.0922881}}	174

Out[614]= {49, 49}

For the 49 branches, these are the # of descendants; times of origin; extent; # SNP; and areas. One branch(#347) is disjunct. Not many are generated by the sweep, or associated with it, in the sense that only 3 begin at zero, and only 9 originate between 40 and 80 generations back (which is when

the sweep is inducing most coalescence)

In[615]:=
```
ppb = Flatten[Position[blB20, #] & /@ blB20notNested];
cols = ColorData["DarkRainbow"] /@ Range[1, 0, -1/(Length[ppb] - 1)];
TableForm[Prepend[Transpose[{ppb, cols, Length /@ blB20notNested[[All, 1]],
    blB20notNested[[All, 2]], Length /@ snpB20[[ppb]],
    branchAreaFull[sweepB20, 0.2][[ppb]], blB20notNested[[All, 3]]}],
  {"index", "colour", "# desc", "t", "# SNP", "area", "{r0,r1}"}], TableDepth -> 2]
```

Out[616]//TableForm=

index	colour	# desc	t	# SNP	area	$\{r_0,r_1\}$
1	■	1	1	36	18.1107	{{0, 0.2}}
2	■	1	1	26	10.734	{{0, 0.2}}
3	■	1	1	27	14.7598	{{0, 0.2}}
6	■	1	1	17	7.57225	{{0, 0.2}}
7	■	1	1	19	8.80658	{{0, 0.2}}
8	■	1	1	16	7.79242	{{0, 0.2}}
9	■	1	1	21	8.53991	{{0, 0.2}}
10	■	1	1	21	11.1378	{{0, 0.2}}
11	■	1	1	14	6.59035	{{0, 0.2}}
13	■	1	1	14	8.79924	{{0, 0.2}}
14	■	1	1	14	5.38478	{{0, 0.2}}
15	■	1	1	7	2.16372	{{0, 0.2}}
16	■	1	1	8	3.74816	{{0, 0.2}}
17	■	1	1	7	4.05649	{{0, 0.2}}
18	■	1	1	15	5.86948	{{0, 0.2}}
19	■	1	1	16	5.21093	{{0, 0.2}}
20	■	1	1	9	3.30748	{{0, 0.2}}
21	■	2	4	35	18.6594	{{0, 0.163959}}
24	■	2	6	20	11.7729	{{0.0377105, 0.2}}
25	■	2	7	17	6.41979	{{0.0422193, 0.185441}}
29	■	2	44	3	3.85298	{{0.0684998, 0.0836397}}
30	■	2	65	10	5.08574	{{0.0352883, 0.049621}}
33	■	2	10	10	2.88222	{{0, 0.0905804}}
35	■	3	10	3	2.10807	{{0.130813, 0.163959}}
40	■	2	14	10	2.81302	{{0.180743, 0.195878}}
44	■	2	14	5	2.73779	{{0.00116641, 0.0129117}, {0
58	■	2	19	5	2.81091	{{0.00845516, 0.0826403}}
63	■	3	23	4	2.52994	{{0, 0.0278147}}
93	■	5	32	7	2.72372	{{0.138979, 0.163959}}
130	■	4	43	8	6.99008	{{0.040501, 0.0555959}}
131	■	5	43	10	4.0976	{{0.0555959, 0.0867949}}
132	■	3	43	7	2.39647	{{0.0935484, 0.122784}}
147	■	5	44	4	2.0393	{{0.0377105, 0.0699155}}
274	■	5	71	13	3.86921	{{0.0720953, 0.0862156}}
292	■	6	73	7	4.93033	{{0.10589, 0.122784}}
293	■	7	73	2	2.33088	{{0.122784, 0.133868}}
326	■	3	86	5	2.28132	{{0.126275, 0.13198}}
347	■	3	174	8	5.69969	{{0.0823478, 0.0840355}, {0.
349	■	16	101	23	13.2438	{{0.0129117, 0.0330864}}
358	■	14	101	12	5.05503	{{0.040501, 0.0509682}}
376	■	4	109	4	2.88023	{{0.0129117, 0.0203284}}
377	■	4	164	12	7.41051	{{0.0207029, 0.0306668}}
379	■	11	109	5	3.09476	{{0.0579233, 0.0602473}, {0.
465	■	6	144	7	2.56487	{{0.117552, 0.126048}}
492	■	14	150	8	3.78986	{{0.100693, 0.105872}}
534	■	6	174	8	3.04678	{{0.1016, 0.105872}}
544	■	13	175	5	2.09885	{{0.106769, 0.111089}}
680	■	18	316	2	2.07546	{{0.080859, 0.0823478}}
694	■	17	340	10	4.14525	{{0.0899643, 0.0922881}}

This shows all 49 of the “substantial” blocks

In[618]:=
```
cf = ColorData["DarkRainbow"];
gg[k_] := Show[plotBranchFull[blB20⟦ppb[[k]]⟧, cf[(k - 1)/Length[ppb]], sweepB20, 0.2],
  Graphics[Prepend[Table[Disk[snpB20⟦ppb[[k]], j, {2, 1}⟧, {0.001, 3}],
     {j, Length[snpB20⟦ppb[[k]]⟧]}], Black]], PlotRange -> {{0, 0.2}, {0, 800}},
  AspectRatio -> 1, PlotLabel -> "branch " ~~ ToString[ppb[[k]]]];
ListAnimate[Table[gg[k], {k, 1, Length[ppb]}]]
```

Out[619]=

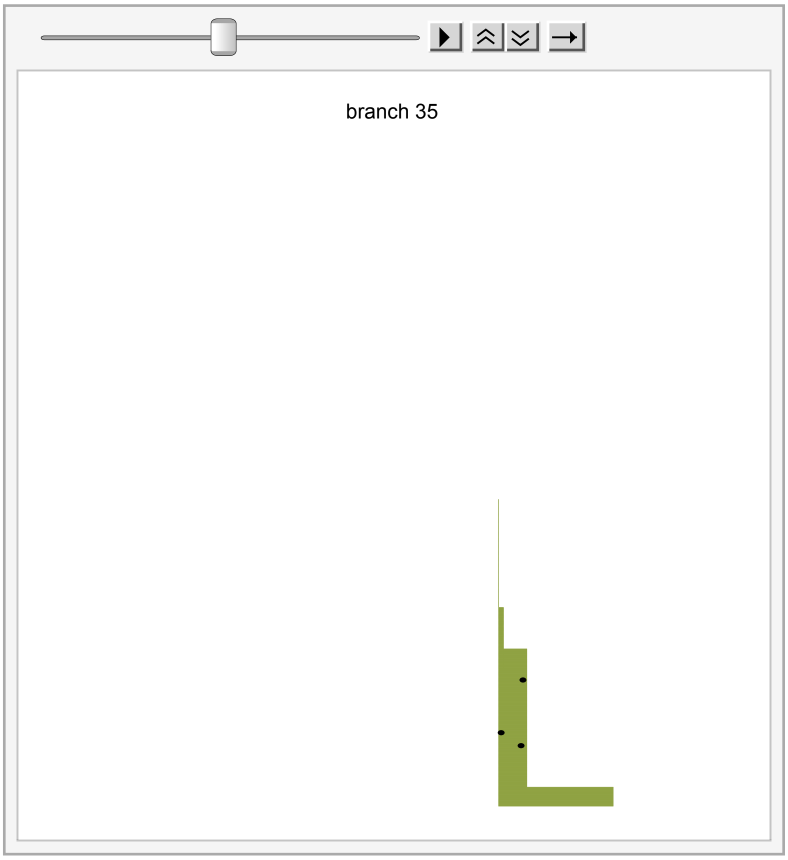

This shows the 30 non-nested, non-tip branches that are associated with the focal genealogy. The light pink rectangle at left is where the whole genealogy coalesces in the sweep. The substantial branches seem to be concentrated near the sweep, even though SNP diversity is high all along the genome, except for the leftmost 1cM

In[632]:=
```
ppb = Flatten[Position[blB20, #] & /@ blB20notNested];
cf = ColorData["DarkRainbow"];
cols = cf /@ Range[1, 0, -1/(Length[ppb] - 1)];
nb = Length[snpB20];
gb[b_, c_] := Show[plotBlockFull[blB20[[b]], Lighter[c, 0.7], sweepB20, 0.2],
   Graphics[{c, PointSize[0.005], plotSNP[b, popB20]}],
   PlotRange -> {{0, 0.21}, {0, 21}}];
cr = coalescedRegions[sweepB20[[70]], 20];
Show[Graphics[
  plotSNP[Complement[Range[nb], ppb], ConstantArray[Gray, nb], 0.003, popB20]],
 MapThread[gb, {ppb, cols}],
 Graphics[
  Join[{Opacity[0.3, Red]}, Rectangle[{#[[1]], 0}, {#[[2]], 20}] & /@ cr]],
 AspectRatio -> 0.2, Frame -> True]
```

Out[636]=

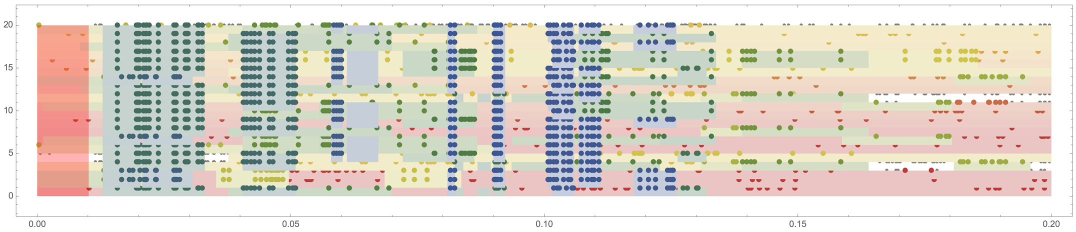

This shows each of the 49 branches separately, together with their time of origin and area:

In[738]:=
```
ListAnimate[Table[Show[gb[ppb[[j]], cols[[j]]], AspectRatio -> 0.2, Frame -> True,
   ImageSize -> Large, PlotLabel -> ("branch " ~~ ToString[ppb[[j]]] ~~
      " t=" ~~ ToString[blB20[[ppb[[j]], 2]]] ~~ " area=" ~~
      ToString[branchAreaFull[sweepB20, 0.2][[ppb[[j]]]]])], {j, Length[ppb]}]]
```

Out[738]=

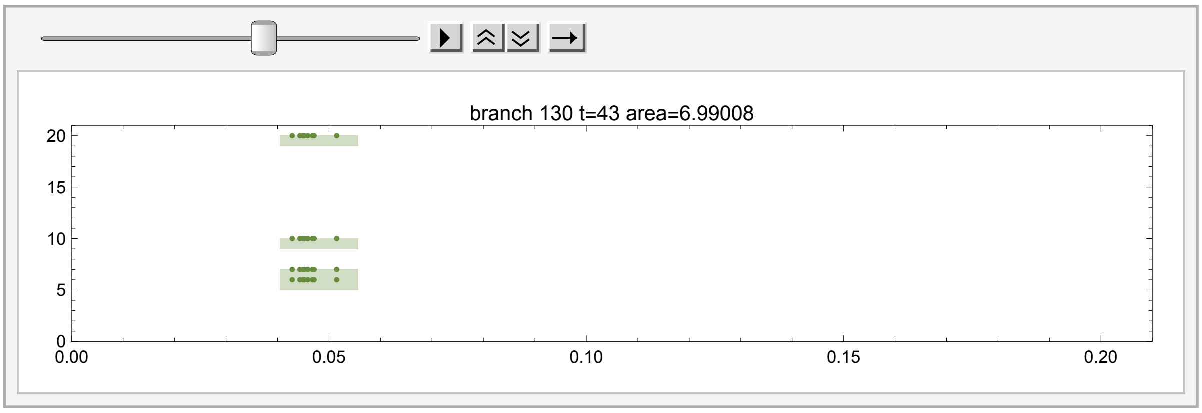

This would give the time spanned by the block - but it is not very helpful

In[735]:=
```
MinMax[posBlockFull[sweepB20, 0.2][[ppb[[2]], All, 1]]]
```

Out[735]=
```
{1, 460}
```

These are all the SNP:

In[640]:=
```
Show[Graphics[plotSNP[Range[nb], ConstantArray[Black, nb], 0.002, popB20]],
 AspectRatio -> 0.2, Frame -> True]
```

Out[640]=

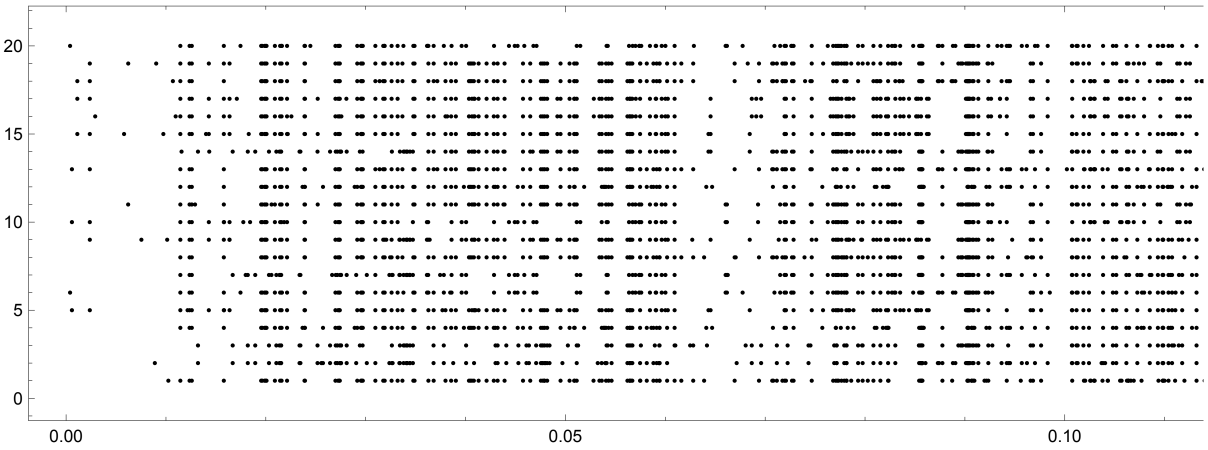

This shows genealogies at 0, 0.01, 0.02, 0.05, 0.20, on two different vertical scales. The first “escape” is just beyond 1cM, but the genealogy still retains the effects of the sweep out to 5cM or so:

In[1325]:=
```
hh[j_, ym_] := Show[PlotGenealogy[genB20[[j]], NodeFunction -> plotCoords[1]],
   PlotRange -> {{-1, 20}, {0, ym}}, Frame -> True,
   FrameTicks -> {None, Automatic}, AspectRatio -> 0.7];
ii = {1, 6, 22, 87, -1};
GraphicsGrid[{hh[#, 150] & /@ ii, hh[#, 800] & /@ ii}]
ints20[[ii]]
```

Out[1327]=

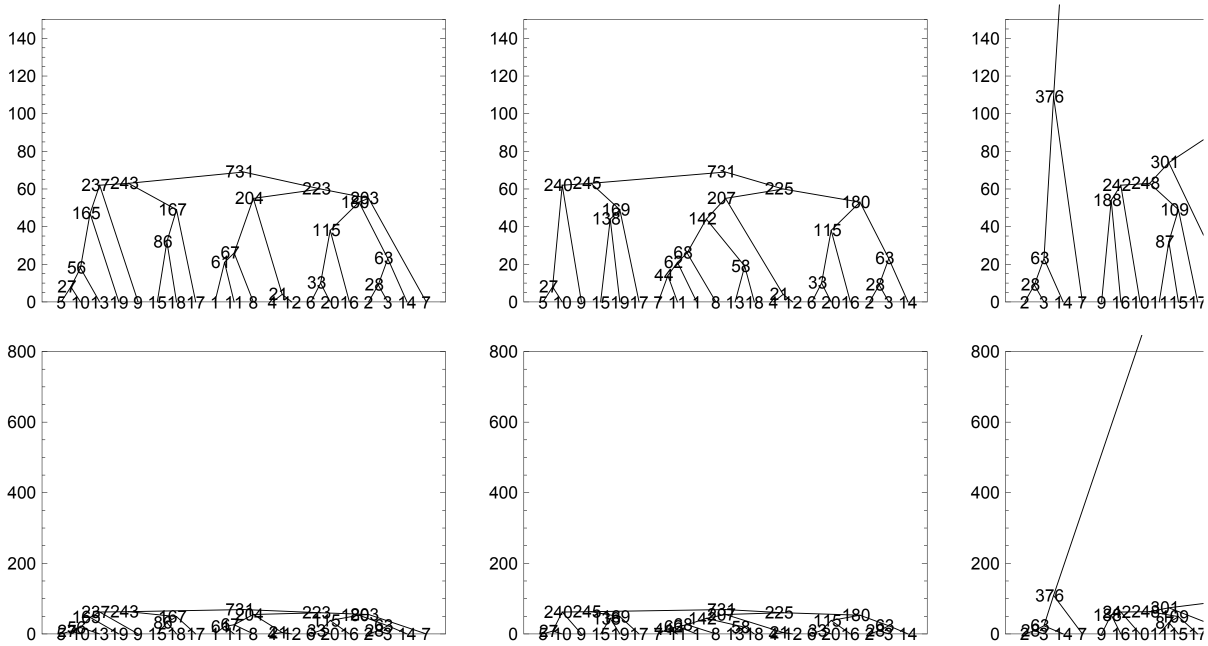

Out[1328]=
```
{{0, 0.00116641}, {0.00845516, 0.0101709},
 {0.0199083, 0.0203284}, {0.049621, 0.0504018}, {0.199885, 0.2}}
```

This shows all 30 of the “substantial” blocks

```
In[1343]:= cf = ColorData["DarkRainbow"];
gg[k_] := Show[plotBranchFull[blB20⟦ppb[[k]]⟧, cf[(k - 1)/Length[ppb]], sweepB20, 0.2],
   Graphics[Prepend[Table[Disk[snpB20⟦ppb[[k]], j, {2, 1}⟧, {0.001, 3}],
      {j, Length[snpB20⟦ppb[[k]]⟧]}], Black]], PlotRange -> {{0, 0.2}, {0, 800}},
   AspectRatio -> 1, PlotLabel -> "branch " ~~ ToString[ppb[[k]]]];
ListAnimate[Table[gg[k], {k, 1, Length[ppb]}]]
```

Showing branches with many descendants

This is the set of branches used in the final figure

This shows the 9 branches with >8 descendants, that originate at or more recently than the mutation; and that have area >0.5. The branches with the largest area, longest extent, and the most descendants tend to be near the sweep, but there are three small branches to the right with 9 or 10 descendants:

```
In[957]:= rn = Range[Length[blB20]]; ba = branchAreaFull[sweepB20, 0.2];
bx = Mean[Flatten[#[[3]]]] & /@ blB20;
blen = (#[[3, -1, -1]] - #[[3, 1, 1]]) & /@ blB20;
long = Select[rn,
    (Length[blB20[[#, 1]]] > 8) ∧ (blB20[[#, 2]] ≤ 101) ∧ (ba[[#]] > 0.5) &];
GraphicsRow[{ListPlot[Transpose[{bx, ba}][[long]], AxesLabel ->
     {"map/position", "branch\narea"}, PlotRange -> {{0, 0.2}, {0, 15}}],
   ListPlot[Transpose[{bx, blen}][[long]], AxesLabel ->
     {"map/position", "branch\nlength"}, PlotRange -> {{0, 0.2}, {0, 0.025}}],
   ListPlot[Transpose[{bx[[long]], Length /@ blB20[[long, 1]]}],
    AxesLabel -> {"map\nposition", "# desc."}, PlotRange -> {{0, 0.2}, {0, 20}}]}]
```

Out[960]=

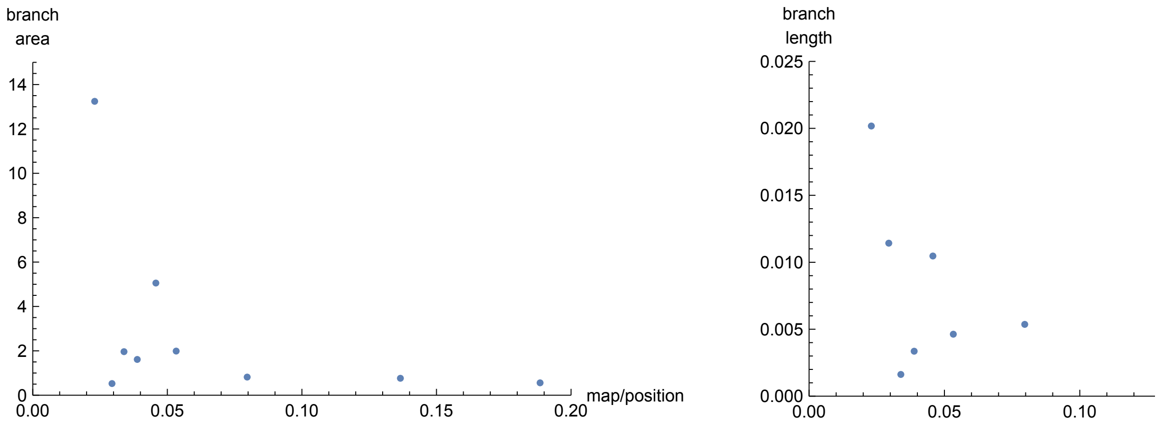

This shows the 20 branches with more than 5 descendants, area >0.5 and originating after the sweep.

Out[1050]=

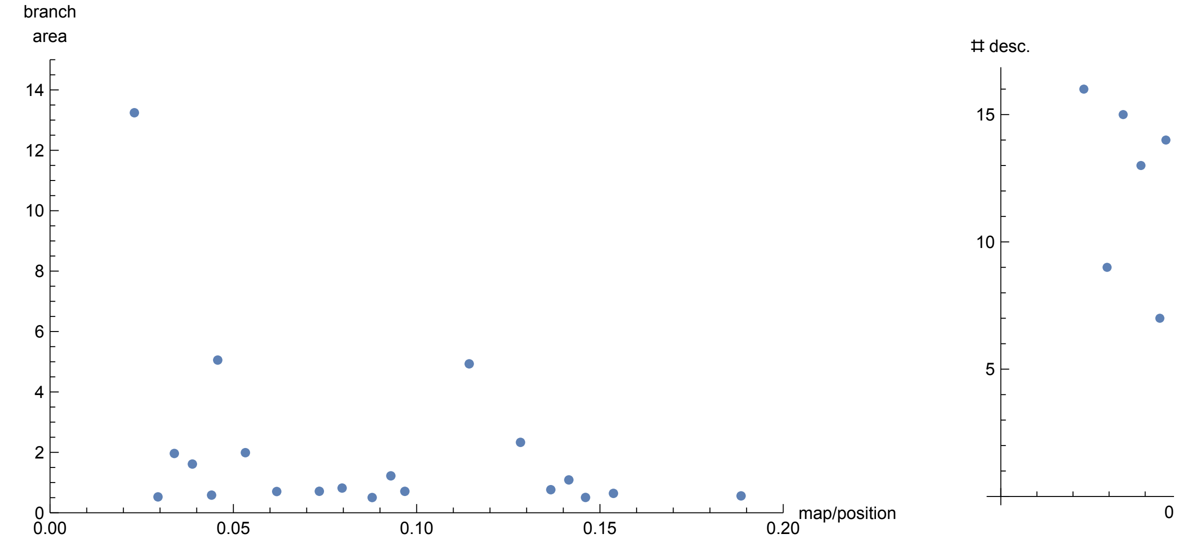

This shows the 9 branches individually. They are typically narrow, consistent with having many descendants and therefore being prone to recombination.

In[1029]:=
```
cf = ColorData["DarkRainbow"];
gg[k_] := Show[plotBranchFull[blB20⟦long[[k]]⟧, cf[(k - 1)/Length[long]], sweepB20, 0.2],
   Graphics[Prepend[Table[Disk[snpB20⟦long[[k]], j, {2, 1}⟧, {0.001, 3}],
      {j, Length[snpB20⟦long[[k]]⟧]}], Black]], PlotRange -> {{0, 0.2}, {0, 800}},
   AspectRatio -> 1, PlotLabel -> "branch " ~~ ToString[long[[k]]] ~~
     " at " ~~ ToString[PaddedForm[blB20⟦long[[k]], 3⟧, 3]]];
ListAnimate[Table[gg[k], {k, 1, Length[long]}]]
```

Out[1030]=

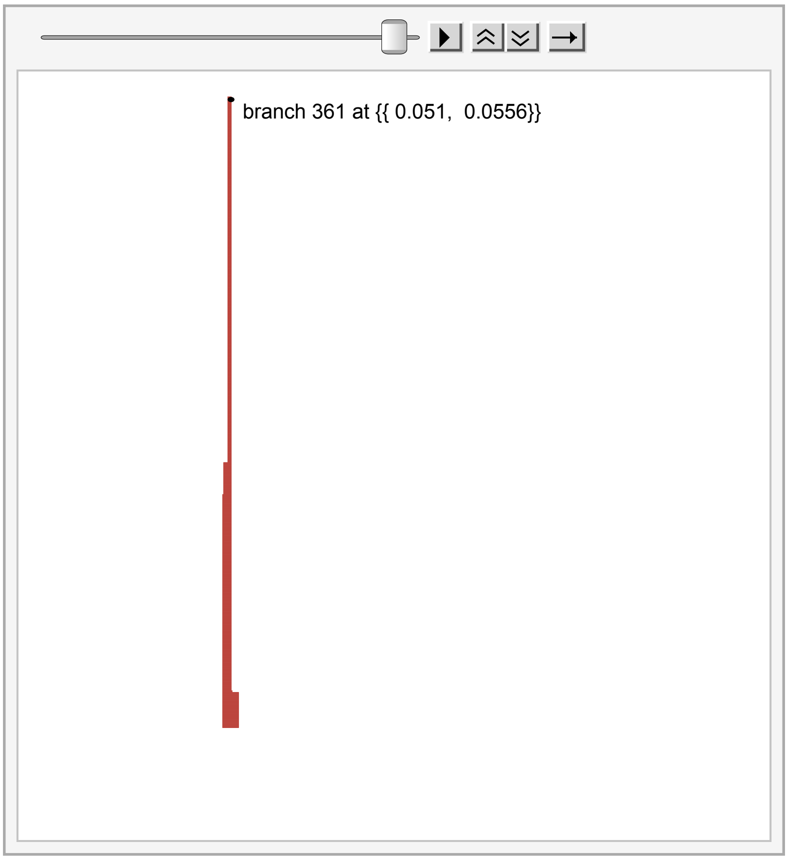

Making replicates

We wish to plot the time to MRCA for ~4 replicate sweeps, to superimpose on the focal sweep. The ancestral genotype, prior to the mutation, is set to zero:

In[1070]:=
```
tMut = bounds[Reverse[selB]][[-1, 1]];
Timing[time = 0; Do[sweepB20rep[j] = makeC[20, 0.2, 400, selB];
   sweepB20rep[j] = Join[sweepB20rep[j][[1 ;; tMut - 1]],
     Replace[sweepB20rep[j][[tMut ;; -1]], {1, x__} :> {0, x}, {2}]], {j, 4}];
 Table[Length[sweepB20rep[j]], {j, 4}]]
```

Out[1071]= {131.056, {5369, 2899, 2560, 2751}}

In[1142]:=
```
cols = {Black, Blue, Purple, Brown};
dgf = genFunction[DepthOfGenealogy, x, genB20, ints20];
Show[LogPlot[dgf, {x, 0, 0.2}, AspectRatio -> 0.15,
   PlotStyle -> Black, PlotRange -> {{0, 0.15}, {40, 4000}}],
  Table[ListLogPlot[Transpose[{Mean /@ intervals[sweepB20rep[j], 0.2],
       timeToMRCA[sweepB20rep[j], 20, 0.2]}], PlotStyle -> Lighter[cols[[j]], 0.6],
    AspectRatio -> 0.15, Joined -> True], {j, 3}],
  LogPlot[110, {x, 0, 0.2}, PlotStyle -> {Black, Dashing[{0.01, 0.01}]}]]
```

Out[1144]=

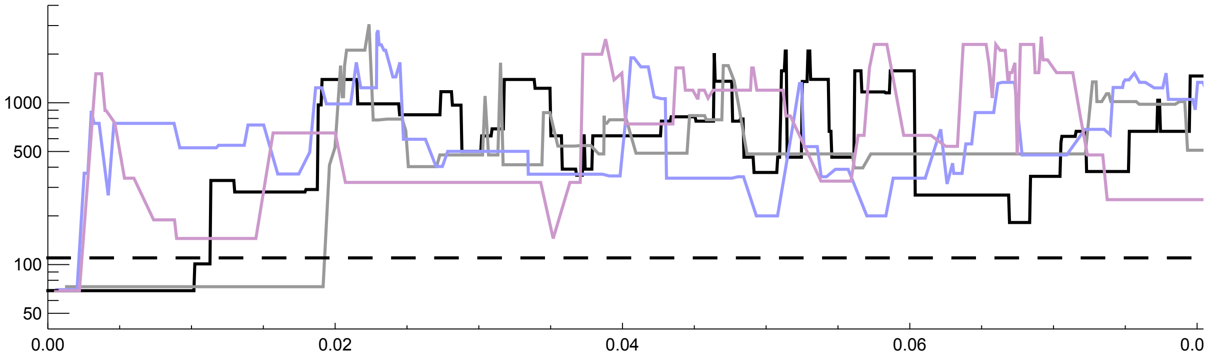

In[1148]:=
```
cols = {Black, Blue, Purple, Brown};
dgf = genFunction[DepthOfGenealogy, x, genB20, ints20];
Show[Plot[dgf, {x, 0, 0.2}, AspectRatio -> 0.15,
   PlotStyle -> Black, PlotRange -> {{0, 0.15}, {0, 2500}}],
  Table[ListPlot[Transpose[{Mean /@ intervals[sweepB20rep[j], 0.2],
       timeToMRCA[sweepB20rep[j], 20, 0.2]}], PlotStyle -> Lighter[cols[[j]], 0.6],
    AspectRatio -> 0.15, Joined -> True], {j, 3}],
  Plot[110, {x, 0, 0.2}, PlotStyle -> {Black, Dashing[{0.01, 0.01}]}]]
```

Out[1150]=

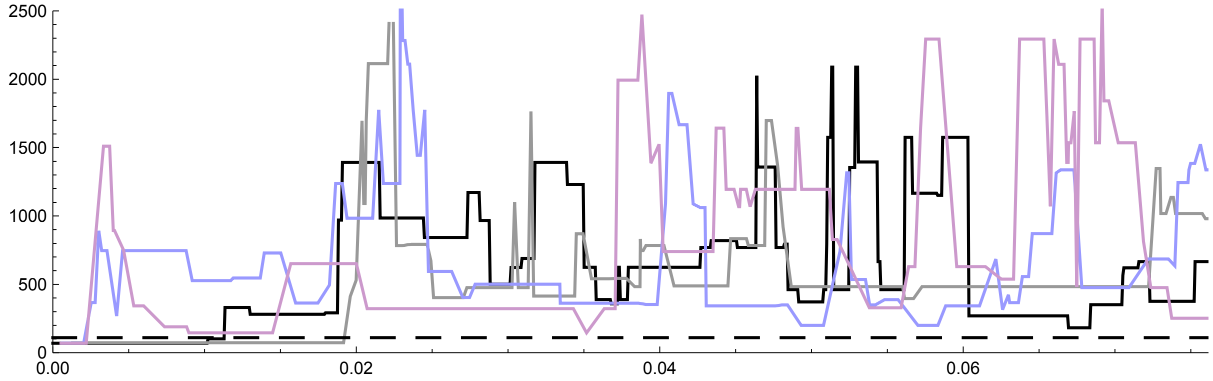

This saves the example: ancestry is enormous, apparently, so we did not save it. Note that there are two sets of genealogies: genB20 which includes only the sampled 20 genomes, and genB20New which includes the selected background and an outgroup. blB20 lists branches only from the first set. *I do not save the genealogies, since these take a long time to construct, and are not needed for the plots. The ancestry stored here also includes ancestry for the main example.*

In[1132]:=
```
Save["Big sweeps s=0.1, 2N=400 R=0.2 20 genomes reps 4 Jan",
  {sweepB20rep, ints20rep}];
```

```
<< "Big sweeps s=0.1, 2N=400 R=0.2 20 genomes reps 4 Jan";
```

Making the figure

This takes a while, starting from the initialisation plus saved data:

In[621]:=
```
rn = Range[Length[blB20]];
ba = branchAreaFull[sweepB20, 0.2];
bx = Mean[Flatten[#[[3]]]] & /@ blB20;
long =
  Select[rn, (Length[blB20[[#, 1]]] > 8) ∧ (blB20[[#, 2]] ≤ 101) ∧ (ba[[#]] > 0.5) &];
```

Allele frequency reaches 0.9, 0.5, 0.1, 1/400 at 22, 29, 38, 53, 100, 110 generations back. One could draw dashed lines at these positions:

In[710]:=
```
110 - pos[Take[selB, -110] / 400, #] & /@ {0.9, 0.5, 0.1, 0.0024}
```

Out[710]= {22, 38, 53, 110}

This makes the genealogy plots:

In[1063]:=
```
mx = 200;
hh[j_] := Module[{mg},
   mg = MakeGenealogyPlot[genB20New[[j, 3, 2]]];
   xy = plotCoords[1][0, mg[[2]]];
   Show[PlotGenealogy[mg, MakeGenealogyPlot -> False,
     NodeLabels -> None, LeafLabels -> None, NodeFunction -> plotCoords[1],
     BranchGraphic -> mosaicBranch[mg, sweepB20,
       ints20[[j]], 0.2, {{Black}, {Red}}]],
    Graphics[{Black, Line[{xy, {xy[[1]], mx}}]}],
    (* vertical branch back from the root *)
    Graphics[Join[{Black, Dashing[{0.01, 0.01}]},
      Line[{{0, #}, {21, #}}] & /@ {110, 53, 38, 22}]],
    Frame -> True, FrameTicks -> {None, Automatic},
    PlotRange -> {{0, 21}, {0, mx}}, AspectRatio -> 0.7]];
ii = {1, 10, 100, 420};
genPlot = GraphicsRow[hh /@ ii]
ints20[[ii]]
```

Out[1066]=
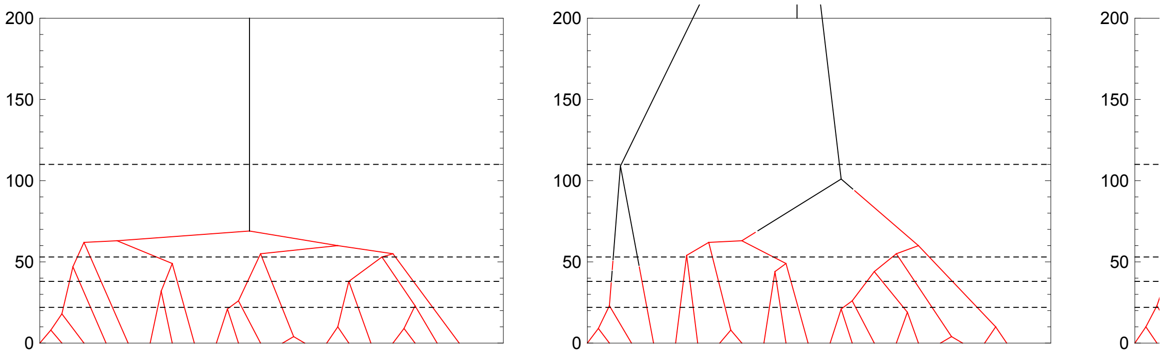

Out[1067]= {{0, 0.00116641}, {0.0129117, 0.0129652},
 {0.0524607, 0.0525217}, {0.197523, 0.198033}}

```
cf = ColorData["DarkRainbow"];
cols = cf /@ Range[1, 0, -1/(Length[long] - 1)];
nb = Length[snpB20];
gb[b_, c_] := Show[plotBlockFull[blB20[[b]], Lighter[c, 0.7], sweepB20, 0.2],
   Graphics[{c, PointSize[0.005], plotSNP[b, popB20]}],
   PlotRange -> {{0, 0.21}, {0, 21}}];
cr = coalescedRegions[sweepB20[[70]], 20, 0.2];
snpPlot = Show[Graphics[plotSNP[
      Complement[Range[nb], long], ConstantArray[Gray, nb], 0.003, popB20]],
   MapThread[gb, {long, cols}],
   Graphics[
    Join[{Opacity[0.3, Magenta]}, Rectangle[{#[[1]], 0}, {#[[2]], 20}] & /@ cr]],
   AspectRatio -> 0.2, Frame -> True];
dgf = genFunction[DepthOfGenealogy, x, genB20, ints20];
pwf = genFunction[MeanPairwiseDivergence, x, genB20, intervals[sweepB20, 0.2]];
```

In[1069]:=

```
GraphicsColumn[{genPlot,
  snpPlot,
  LogPlot[pwf, {x, 0, 0.2},
   AspectRatio -> 0.15, PlotRange -> {{0, 0.2}, {40, 4000}}],
  LogPlot[dgf, {x, 0, 0.2}, AspectRatio -> 0.15,
   PlotRange -> {{0, 0.15}, {40, 4000}}]}]
```

Out[1069]=

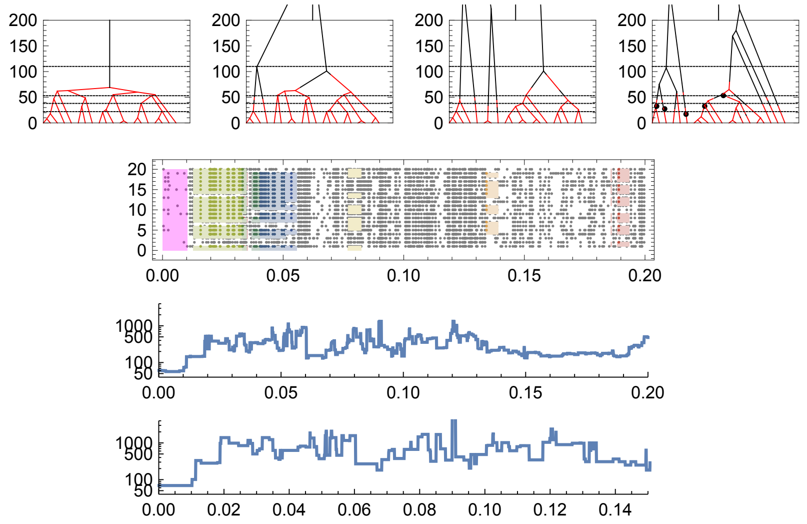

Figure. The top row shows four genealogies, at map positions 0, 0.013, 0.053, 0.198; the leftmost genealogy is at the selected locus. Red indicates lineages associated with the fitter allele, and black the ancestral allele; all lineages must recombine onto the ancestral (black) background by the time of the sweeping mutation, 110 generations into the past. I have not got the colouring of the lines quite right yet. The top left panel shows the (log) frequency of the sweeping allele on the same timescale. *It could be better to drop this panel, and instead draw horizontal dashed lines on the genealogies at times 110,53,38,22, when p=1/400, 0.1, 0.5, 0.9.* The second row shows the 20 genomes, highlighting the 9 branches that have more than 8 descendants, that formed by coalescence more recently than the sweeping mutation, and that have areas >0.5. The magenta block at

the right shows the region linked to the selected locus, that coalesces in a single common ancestor at generation 69, just after the sweeping mutation arose. Grey dots show SNP that are not on these 9 highlighted branches. Note that there is some diversity within the magenta area, due to mutation subsequent to the sweep. The third row shows mean pairwise divergence, and the fourth row the time back to the MRCA, bith on a log scale.

```
Out[906]= {{0, 0.00116641}, {0.0129117, 0.0129652},
 {0.0524607, 0.0525217}, {0.197523, 0.198033}}
```

Definitions

Generating the sweep trajectory

Simulating the ARG, conditional on a sweep

Adding SNP to the ARG

Describing the ARG

Constructing genealogies from the ARG

Plotting the ARG

Approximating probabilities of coalescence

Identity by descent in a selective sweep

General utilities
