# Supplementary material for: On the origin and structure of haplotype blocks
Source: Mol Ecol. 2022 Dec 15;32(6):1441–57. doi: 10.1111/mec.16793 (PMC10946714; doi:10.1111/mec.16793)
Supplement: Supplementary file 2 — Appendix S2: [file MEC-32-1441-s001.pdf]

MOLECULAR ECOLOGY

Supplemental Information for:

On the origin and structure of haplotype blocks

Daria Shipilina*[1,2,3$], Arka Pal[2]*, Sean Stankowski*[2], Yingguang Frank Chan†[4], Nicholas H. Barton†[2]

Table of Contents:

**Supplement 1:** Summary of the simulation	**Available on GitHub:** https://github.com/DaSh-bash/Suppl_Materials_On_the_origin_2022/Supplement_1.nb
**Supplement 2:** ARGweaver analysis	**1-11**

ARGweaver Analysis

This document outlines the pipeline followed to obtain **Figure 6**. The associated .Rmd file (https://github.com/DaSh-bash/Suppl_Materials_On_the_origin_2022/Supplement-Information-2.Rmd) provides the detailed code to reproduce results.

1. Running ARGweaver

1.1. Sample information

ARGweaver is applied on a phased SNP dataset of *Heliconius erato* butterflies, sequenced by *haplotagging*, a technique that produces linked-read sequence data. We used two previously published genomic regions - *Herato1801:1362067-1405605* (coincides with the previously identified gene *optix* that has undergone a selective sweep) and *Herato1603:3450000-3493538* (a neutral background region) (Meier et al, 2021). 10 individuals of *H.e.lativitta* and *H.e.notabilis* were chosen from opposite ends of the hybrid zone transect for ARG inference.

1.2. Preprocessing files for ARGweaver

To preprocess the input files for ARGweaver, we first subset the 20 diploid individuals from full VCF files (see Supplementary Information in Meier et al, 2021), and then convert the SNP information into the *.sites* format required by ARGweaver. The *.sites* format only contains information on the positions that are varying within the 20 individuals that we chose.

In the *optix* region (43538 bp long), there are 2812 sites altogether - 2426 are variant positions, while 330 and 56 sites are fixed for one or the other allele. Genomic positions that are neither variant nor fixed to one or the other allele (in other words, positions absent in the VCF file) are considered missing information and therefore masked from being used as input data for ARGweaver. Altogether, ARGweaver uses information of variant alleles and invariant alleles; whereas the rest is masked and treated as missing information.

Similiarly, the neutral region, *Herato1603:3450000-3493538* (43538 bp) has 6407 sites altogether - 4926 is variant positions, while 1405 and 76 sites are fixed for either allele.

1.3. Input parameters

In order to run ARGweaver, we consider mutation rate, $\mu = 2.9 \times 10^{-9}$ per bp per generation, and its ratio to recombination rate, $\frac{\mu}{r} = 1$. We estimate N_e by calculating Tajima's π $(= 4N_e r)$ from the neutral region. $\pi = 0.0225049$, $N_e = 1940078$. The total map length of both *optix* and neutral region is 0.01262602 cM.

```r
## Calculating Ne from Herato1603:3450000-3550000

r = 2.9e-09  # recombination rate
L = 3550000 - 3450000  # Genomic length
n = 40  # no. of samples
pi_sum = 2250.49  # Sum of pi estimates across all sites
pi = pi_sum/L  # pi estimate
S = 10571  # No of seggregating sites in this region
Ne_tajima = pi/(4 * r)  # Tajima's Ne
Ne_watterson = S/(4 * r * L) * (sum(c(1:(n - 1))^(-1)))^-1  # Waterson's Ne
```

```
## pi = 0.0225049
## Ne_tajima = 1940078
## Ne_watterson = 2142433
## Ne_tajima/Ne_watterson = 0.9055488
## Map Length = 0.01262602 cM
```

ARGweaver allows coalescence and recombination events to take place only at discretized time points, defined by the function, $t(i) = \frac{exp(\frac{i}{K1}log(1+\delta t_{max}))1}{\delta}$; for K time points and $i \in \{0, 1, \ldots, K1\}$. Very small values of $\delta(< \frac{1}{t_{max}})$ will yield roughly linear distribution of times, whereas larger values of δ will place more time points in recent past and less in deep past. For this analysis, we set $K = 30$, the maximum time for total coalescence $(t_{max}) = 20N_e$, and the shape parameter $(\delta) = 0.01$ (*Figure S1*).

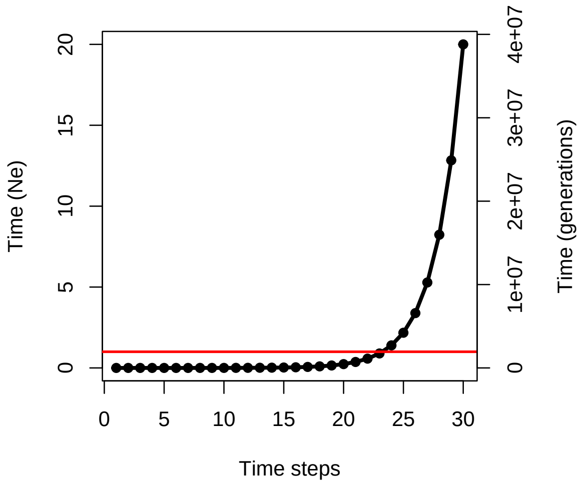

Figure S1: Discrete time points for ARGweaver analysis - 30 time steps, $N_e = 1940078$, Left y-axis shows the time points in N_e, while right y-axis in number of generations. Red line denotes $1N_e$.

1.4. Run ARGweaver

ARGweaver is run for 5000 iterations and then resumed for another 5000 iterations for the *optix* region, whereas only 5000 iterations for the neutral region.

```
# Run ARGweaver
~/.local/bin/arg-sample -s  sites/${regionName}_Herato.sites \
    --maskmap masks/${regionName}_Herato_mask.bed.gz \
    -N 1940078 -m 2.9e-9 -r 2.9e-9 --ntimes 30 \
    --maxtime $((1940078*20)) --iters 10000 --delta 0.01 \
    --output {regionName}/sample/${regionName}
```

2. Analysis of ARGweaver output

2.1. MCMC summary

After visualizing MCMC traces of likelihood, prior and joint probabilities, we decided to set the first 3000 iterations as burnin. The plot below is for the *optix* region (*Figure S2*).

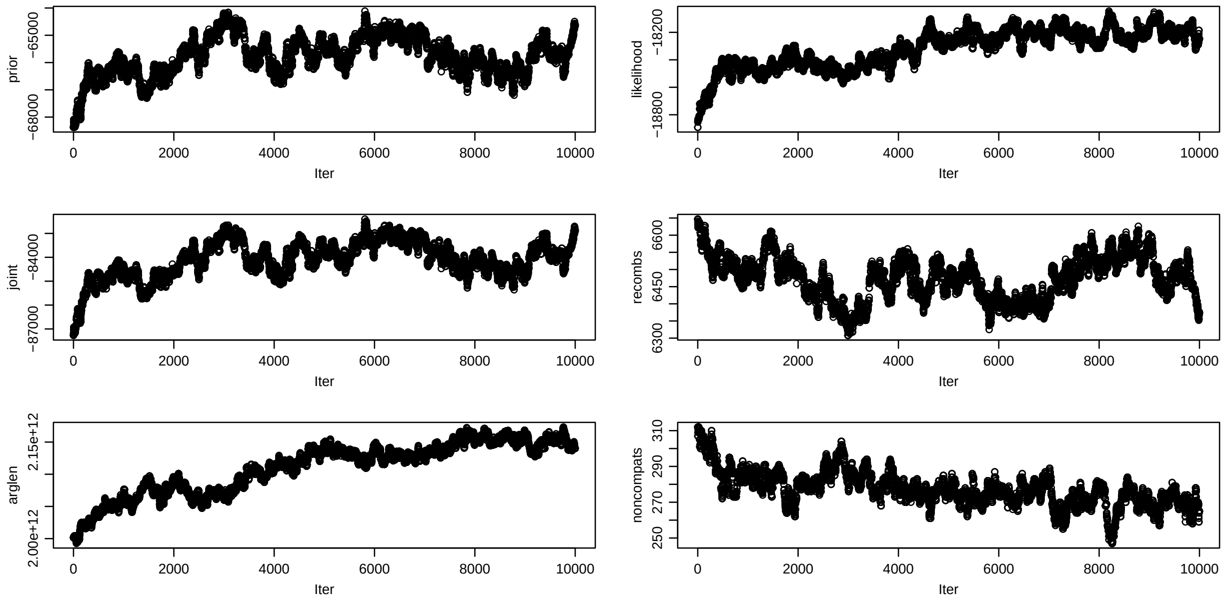

Figure S2: Traces across all MCMC iterations of prior (log probability of the sampled ARG given the model), likelihood (log probability of the data given the sampled ARG), joint - (total log probability of the ARG and the data; prior + likelihood), recombs - (number of recombination events in the sampled ARG), arglen (total length of all branches summed across sites) and noncompats (number of variant sites that cannot be explained by infinite sites mutation model).

2.2. ARGweaver output

The ARGweaver output, *.smc* files are then converted to *.bed* format to extract TMRCAs, trees, recomination breakpoints and total tree branch lengths across all iterations (except burnin). Two iterations (Iteration 8250 and 9200) are chosen for further analysis and identification of haplotype blocks.

2.3. TMRCA

We first examine the TMRCA of the total tree and the individual populations - *H.e.lativitta* (in red) and

H.e.notabilis (in yellow) (*Figure S3*). Unlike the neutral region (*Herato1603*) where all TMRCA estimates are fairly constrained between 1 and 10 N_e, TMRCA traces for individual populations in the *optix* region exhibit shallow coalescence times at multiple positions throughout the entire ~50kb region. In case of a selective sweeps, where a beneficial mutation sweeps through the population, TMRCA tends to be shallow since all samples in the swept population coalesces quickly to the initial “lucky” ancestor. Moving away from the focal mutations, lineages recombine away from the swept ancestral background. The TMRCA estimates from the *optix* region is consistent with the previously identified selected region in *optix*. To investigate further the haplotype block structures, we focus into a 3kb region at optix:1385966-1388966.

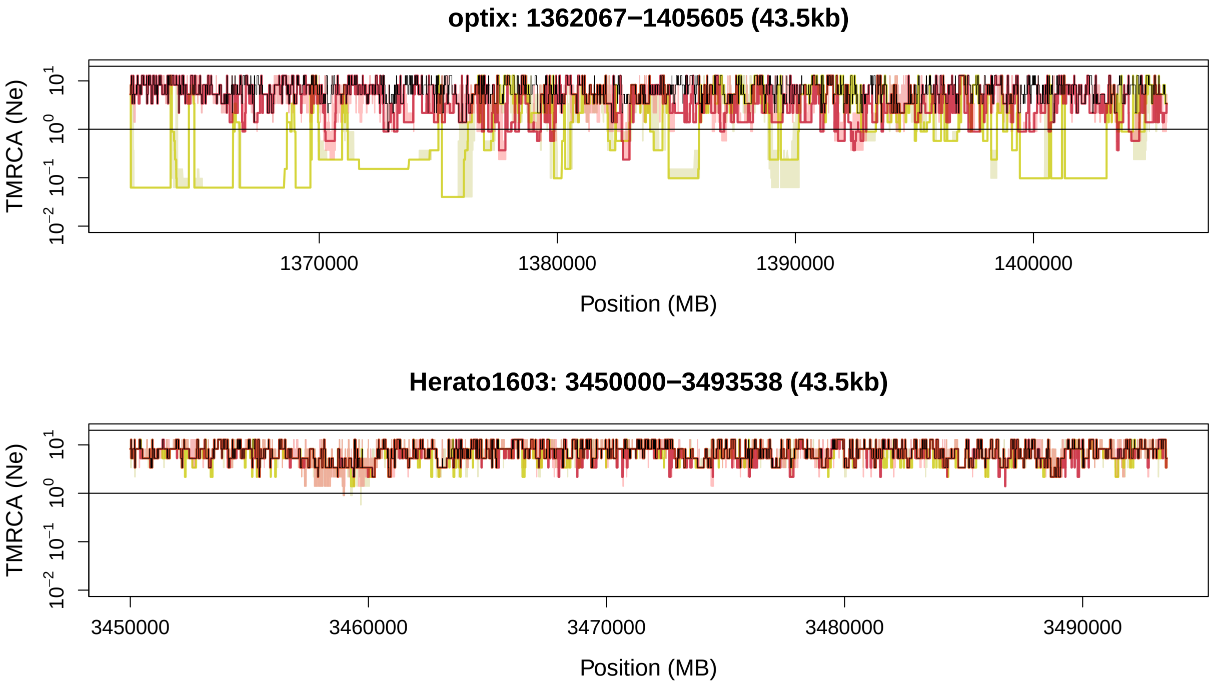

Figure S3: TMRCA (Ne) for each position in the *optix* and the *neutral* genomic region. Black line: total TMRCA, Red: Median TMRCA for *H.e.lativitta*, Yellow: TMRCA for *H.e.notabilis*. Corresponding shaded regions are the 0.05 and 0.95 percentile intervals.

In the focal region, there are 137 SNPs. SNPs are changed from its nucloetide asignment to 0/1; based on allele frequency within the *H.e.lativitta* samples. (Higher allele frequency - 1, Lower allele frequency - 0)

Masking sites can have a critical effect on sampling ARGs, since invariant sites can shift priors towards a recent coalescence (because there hasn’t been enough time yet for a mutation to occur in any of the branches), whereas missing information does not shift priors and therefore ARGs are sampled neutrally in those regions. In the above region of 3kb, there are 137 SNPs and 16 invariant positions. Out of the 137 SNPs, only 59 SNPs are segregating within the *H.e.lativitta* population in focus (*Figure S4*)

Hereafter, alleles with lower frequency within the *H.e.lativitta* population is referred to as the minor allele, and individuals who share the minor allele is the minor clade. Conversely, individuals that carry that major allele is called major clade. Minor allele is coded as 0 and major as 1.

2.4. General comments on identifying haplotype blocks as edges from empirical datasets

In a series of marginal trees along the genome, an edge is considered unique if it originates at a particular coalescent time point, and is ancestral to a given set of samples. Unique edges extend along the genome until

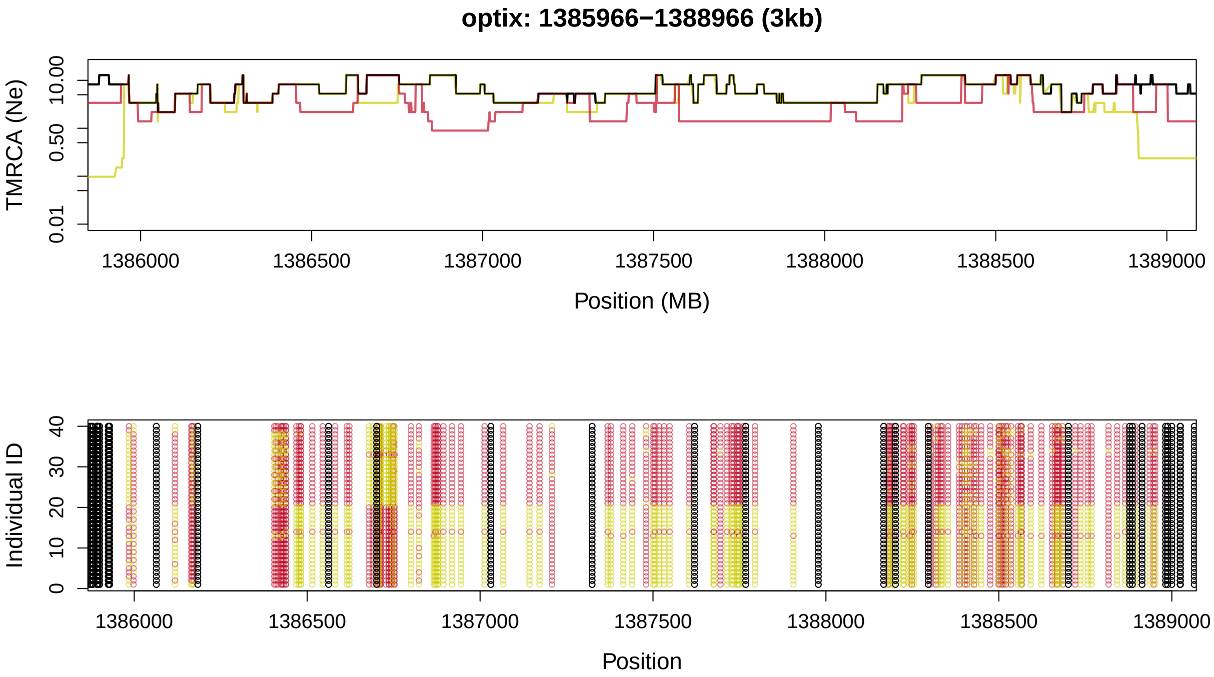

Figure S4: TMRCA for each position in focal genomic region (optix:1385966-1388966). Same colour schemes as above. **Top panel:** TMRCA plot, **Bottom panel:** SNPs in both populations; RED and YELLOW alleles are variant positions, coloured according to the respective higher and lower allele counts in the *H.e.lativitta* population. BLACK alleles are fixed (invariant) in both populations. All other positions (in white) are unknown, since those positions do not have SNP information. These unknown positions are masked while running ARGWeaver, therefore they are treated as missing information and NOT as invariant sites.

recombination breaks them down, but can further re-emerge in a disjunct genomic region if recombination brings the given set of samples back together. Moreover, each edge goes back in time until further coalescence events in deeper past. (*Figure 3* in Main Text, and *Box 2*). Unlike simulations where the ground truth about origin and extent of each unique edge is precisely known, inferred edges from sampled ARGs are consistent with the data, but not necessarily supported by the SNP configuration. Those edges on which mutations appear can be reliably inferred given the SNP configuration, whereas the rest are random samples from the posterior distribution and is subject to stochastic noise, suggesting inference be made from edges supported by SNPs.

ARGweaver outputs genealogical trees along the genome, each recombination breakpoint and its timepoint. For each tree, nodes and tips are labelled uniquely. For each recombination event, the new ancestral node ID of the recombined branch becomes the old node ID from the previous tree. Therefore, across trees, all nodes except the one that underwent recombination in the preceding tree shares the same node ID. Although it is easy to track unique nodes over a short genomic distance by just tracking the SPR (subtree prune and regraft) event; one needs to develop more sophisticated algorithm to track over longer distances than considered in this analysis (which we did not attempt here!). Instead, we leveraged the artifact left behind by time-discretization in ARGweaver to identify unique edges.

The exact step-by-step process that we used to identify edges supported by SNPs is detailed in the associated *.Rmd* file. We specifically chose 2 separate iterations (iteration IDs - 8250 and 9200), sufficiently separated to avoid autocorelations between them to demonstrate the noise in identifying haplotype blocks as edges based on ARG samples. We only focus on the *H.e.lativitta* population (*always in red labels*).

3. Specific MCMC iterations

3.1 Case 1: Iteration 8250

We extracted ARGs sampled by ARGweaver in its MCMC iteration: 8250, and estimated the TMRCA (total and within population), total branch length of each marginal tree and the recombination breakpoints. Altogether in the ~50kb region, there are 6571 trees (6570 recombination events), of which only 464 trees are present in the focal 3kb region, *optix:1385966-1388966*.

We investigate the distribution of branch length of trees with respect to their genomic spans (*Figure S5*). Average tree span in the whole region is ~7 bp. From theory assuming standard coalescence, $P_r(d \mid \tau)=\frac{\rho}{2}L(\tau)$ $\exp[-\frac{\rho}{2}L(\tau)d]$ where $L(\tau)$ is in coalescent unit of $2N_e$ generations and $\frac{\rho}{2}=2N_e r$ denotes the population-scaled recombination rate per basepair. Given the parameters, and mean $L(\tau) = 2$,mean $d = \frac{1}{\frac{\rho}{2}L(\tau)}$ should be ~ 45bp. In this region, ARGweaver seems to change the tree topology more often than expected. We have not explored closely the cause of this (since it is beyond the main message of this analysis), however, we note that although the ARGs are consistent with the data, we need to take caution in biological inference from these sampled ARGs.

For our analysis, we strictly focus on identifying edges supported by SNPs. In practice, this is done in few steps - First, for each sampled tree along the genome (= 464 trees), we extract the following information - ancestral and descendant node of each edge, edge height (=length), time-point of the descendant node, i.e., when the edge originated (=depth, NOTE: due to rounding errors in Newick format, we round the depth values to 3 significant digits) and the samples (=tips.from.dec) that each edge is ancestral to. NOTE: Although we are identifying haplotype blocks in the *H.e.lativitta* (red) population, we use genealogical trees that include both populations. This is done in order to estimate the edge height of the most recent common ancestor to all individuals in the red population. Ideally for making biological inferences from only one population, this need not be done. However in our analysis, in order to illustrate the haplotype block patterns generated in a selected genomic region, we decided to incorporate both populations to generate trees along the genome. Nevertheless, it is important to note that the identified haplotype blocks will stay the same irrespective of which and how many populations are included in the analysis; however, the edge height will change along the genome.

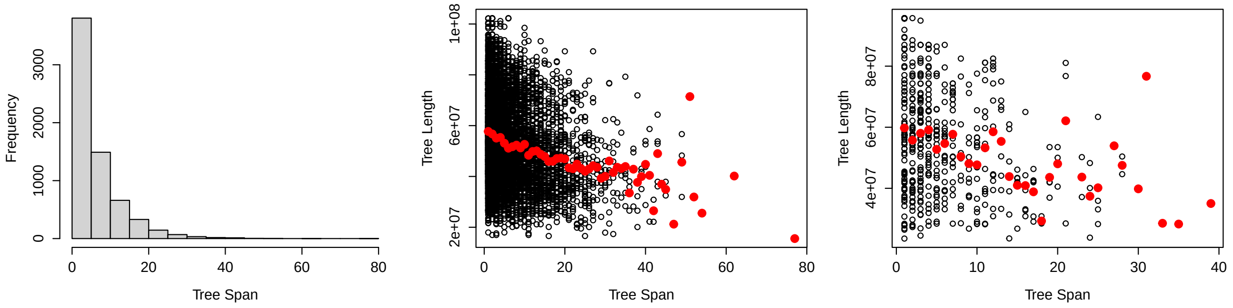

Figure S5: **Left:** Histogram of tree spans; **Center:** Branch length vs tree span for all 6571 marginal trees along the genome; **Right:** Branch length vs tree spans for 464 trees in the focal genomic region. RED points are average tree lengths for each tree span.

Second, for every tree at each SNP position (= 137 SNPs), we identify most recent tree node that is commonly ancestral to all individuals that share the same allele. (Note: this assumes infinite sites mutation model, which is generally the default option in ARGweaver). This allows identification of 1 node for the major and 1 for the minor allele at each SNP position, hereafter called major and minor node for each tree.

The table below illustrates information from the tree whose genomic span coincides with the first SNP position - 1385985.

anc.label	dec.label	length	depth	tips.from.dec
66	75	3.153	0.236	18, 22
75	18	0.236	0.000	18
75	22	0.236	0.000	22
66	72	2.494	0.895	39, 33, 11, 34, 0 , 14, 3 , 9 , 37, 15, 32, 25, 21, 19, 30, 17, 26, 13
72	76	0.798	0.097	39, 33
76	39	0.097	0.000	39
76	33	0.097	0.000	33
72	55	0.321	0.574	11, 34, 0 , 14, 3 , 9 , 37, 15, 32, 25, 21, 19, 30, 17, 26, 13
55	40	0.423	0.152	11, 34
40	11	0.152	0.000	11
40	34	0.152	0.000	34
55	68	0.000	0.574	0 , 14, 3 , 9 , 37, 15, 32, 25, 21, 19, 30, 17, 26, 13
68	0	0.574	0.000	0
68	63	0.000	0.574	14, 3 , 9 , 37, 15, 32, 25, 21, 19, 30, 17, 26, 13
63	14	0.574	0.000	14
63	46	0.206	0.369	3 , 9 , 37, 15, 32, 25, 21, 19, 30, 17, 26, 13
46	60	0.000	0.369	3, 9
60	3	0.369	0.000	3
60	9	0.369	0.000	9
46	44	0.000	0.369	37, 15, 32, 25, 21, 19, 30, 17, 26, 13
44	57	0.000	0.369	37, 15, 32, 25, 21, 19, 30, 17
57	37	0.369	0.000	37
57	77	0.000	0.369	15, 32, 25, 21, 19, 30, 17
77	52	0.000	0.369	15, 32, 25
52	15	0.369	0.000	15
52	54	0.329	0.040	32, 25
54	32	0.040	0.000	32
54	25	0.040	0.000	25

anc.label	dec.label	length	depth	tips.from.dec
77	53	0.132	0.236	21, 19, 30, 17
53	21	0.236	0.000	21
53	65	0.000	0.236	19, 30, 17
65	73	0.085	0.152	19, 30
73	19	0.152	0.000	19
73	30	0.152	0.000	30
65	17	0.236	0.000	17
44	71	0.271	0.097	26, 13
71	26	0.097	0.000	26
71	13	0.097	0.000	13
-1	66	38801556.610	3.390	18, 22, 39, 33, 11, 34, 0 , 14, 3 , 9 , 37, 15, 32, 25, 21, 19, 30, 17, 26, 13

Table S1: For the above tree at SNP position 1385985, individuals 18 and 22 share allele 0, and therefore their most recent common ancestor is node ID 66 which originated at time (in N_e) = 0.236 and has a length of 3.153 (in N_e). anc.label - ancestor ID, dec.label - Descendent ID, length - length of branch between ancestral node and descendent node, depth - Time of descendent node from present, tips.from.dec - all children ID that descendents from the descendent node.

Third, for each minor node (and major node if the SNP is fixed within the *H.e.lativitta* population) identified from trees at each of the 137 SNPs, we identify all trees along the genome which contains that unique ancestral node. Since we do not know the ancestral reference allele at each SNP position and we assume infinite sites mutation, any minor node that is ancestral exclusively to the minor clade is assumed to contain the causal alternate allele and is considered as a branch on which a mutation has occurred.

Following the above steps, we can identify each major/minor node ancestral to each major/minor clade and occurs at a particular time point in the past, that in practice informs us of all the edges informed by SNPs. With 137 SNPs, we have 36 edges supported by SNPs (table below).

depth	Tips from Descendents	snpID	#	edgeID
0.3686	9 , 3 , 11, 21, 14, 34, 32, 25, 26, 13	8, 11, 12	3	1
5.2829	13, 30, 9 , 25, 11, 18, 34, 17, 32, 26	111	1	2
0.5744	3 , 15, 19, 14, 25, 33, 9 , 21, 22	99, 100	2	3
0.8953	37, 3 , 19, 14, 21, 22, 0 , 39, 33	106	1	4
1.3954	30, 0 , 39, 33, 18, 34, 17, 32, 26	113	1	5
0.2365	39, 11, 18, 17, 34, 32, 26	102	1	6
2.1748	17, 33, 18, 15, 19, 22	7	1	7
0.3686	21, 14, 32, 25, 26, 13	13	1	8
0.8953	30, 0 , 39, 11, 19, 14	88	1	9
NA	19, 0, 33, 30, 22	6	1	10
NA	9, 13, 14, 37, 22	40	1	11
0.5744	30, 18, 32, 34	61	1	12
1.3954	34, 30, 17	9, 10, 15	3	13
0.2365	14, 9 , 37	43	1	14
0.2365	9 , 33, 19	80	1	15
0.2365	18, 22	1, 2, 3	3	16
0.1517	22, 17	56	1	17

depth	Tips from Descendents	snpID	#	edgeID
0.0624	21, 22	92, 122, 126	3	18
NA	9, 11	97	1	19
1.3954	30, 13	105	1	20
0.0000	11	5	1	21
0.0000	30	14, 34, 70, 91, 110, 120, 127, 131, 136	9	22
0.0000	34	17, 109	2	23
0.0000	13	25, 26, 27, 29, 32, 33, 35, 36, 37, 38, 41, 112, 116, 118	14	24
0.0000	17	31	1	25
0.0000	3	60	1	26
0.0000	15	108	1	27
0.0000	18	117	1	28
3.3896	ALL	4, 16, 42, 54, 62, 64, 65, 66, 93, 94, 95, 96, 98, 101	14	29
2.1748	ALL	18, 19, 21, 22, 23, 24, 63, 119, 121, 123, 124, 125, 128, 129, 135, 137	16	30
1.3954	ALL	20, 57, 58, 59, 67, 68, 69, 71, 72, 73, 74, 75, 76, 77, 78, 79, 81, 82, 83, 84, 85, 86	22	31
12.8325	ALL	28, 30, 39	3	32
0.8953	ALL	44, 45, 46, 47, 48, 49, 50, 51, 52	9	33
5.2829	ALL	53, 87, 103, 104, 130, 132	6	34
8.2336	ALL	55, 89, 90, 107, 114, 115, 133, 134	8	35

Table S2: All 36 haplotype blocks as edges from iteration 8250; each row represents an unique edge. As mentioned above, each edge is defined uniquely by its origin time-point (=depth) and the set of descendent individuals (=tips.from.dec). For example, edgeID 16 (same edge as the Table S1, S2 refers to) originates at 0.2365 (time in Ne), shared by individuals 18, 22 and is supported by 3 SNPs at position (rank sum order) 1,2,3. Edges with depth = NA refers to SNPs that are incompatible with the infinite sites mutation model, and hence cannot be explained by one mutation event.

3.1.1 Haplotype block visualization

For visualization, we choose to only plot the edges that are supported by 3 or more SNPs. Moreover, for SNPs that are fixed in the red population, we only show edges that originate at any time-point below $5N_e$. This leaves us with only 8 edges.

Now, ploting all the haploype blocks except singletons (*Figure S7*), same as the figure in main text (see Main Text for caption).

3.2. Case 2: iteration 9200

There are altogether 6457 trees in the ~50kb region, and 425 in the focal genomic region.

3.3 Hapotype blocks from both iterations

Figure S8 shows the haplotype blocks and the SNPs that support each block from both iterations.

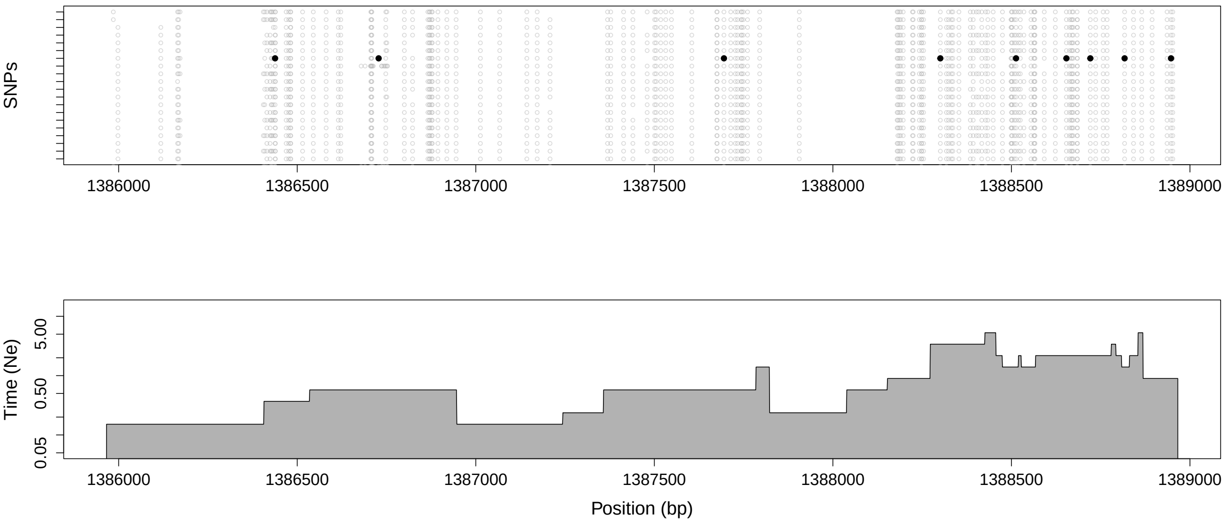

Figure S6: Haplotype block that is supported by singletons. These edges originate directly from the tree tips, ie, the samples and therefore extends all along the genomic region. Although for biological inference, singletons are often uninformative, this shows the feature of an edge supported by singletons, which extends all along the genome, is normally shallow with certain regions that go are high, where a lot of singleton clusters together. These are normally regions of the genome, which have recombined out and goes all the way back to an ancestor in the deep past. The orange block shows clustering of SNPs in the higher region of the edges, whereas, the green edge shows how SNPs can also occur by chance at other regions.

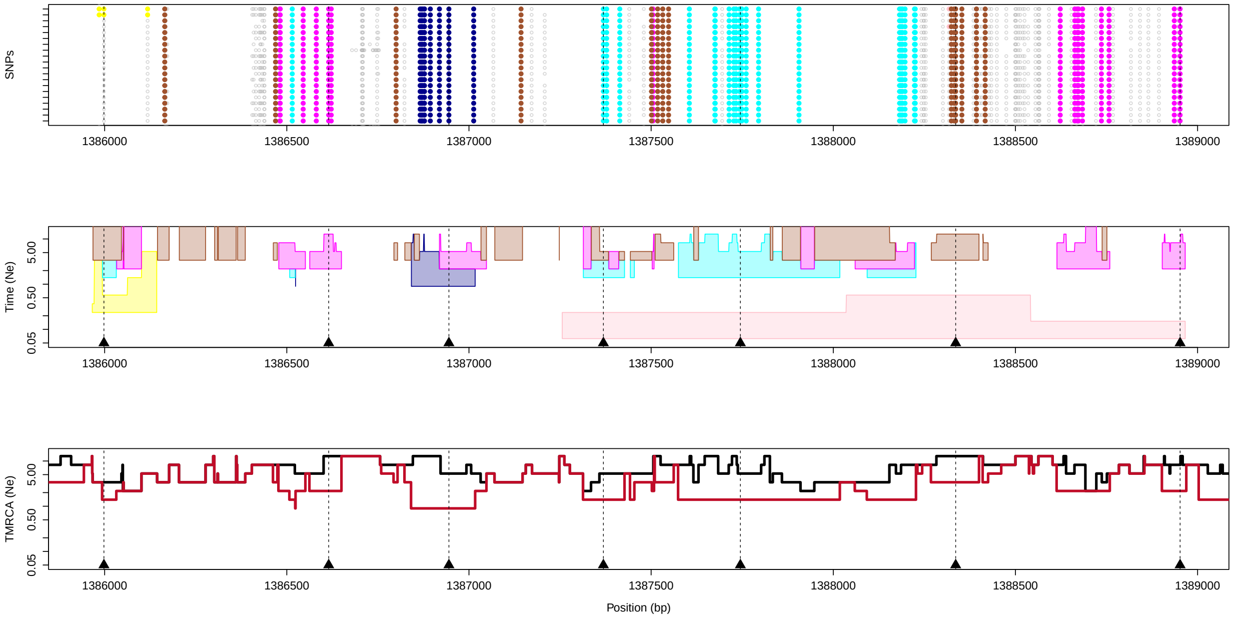

Figure S7: Figure 6 from Main Text

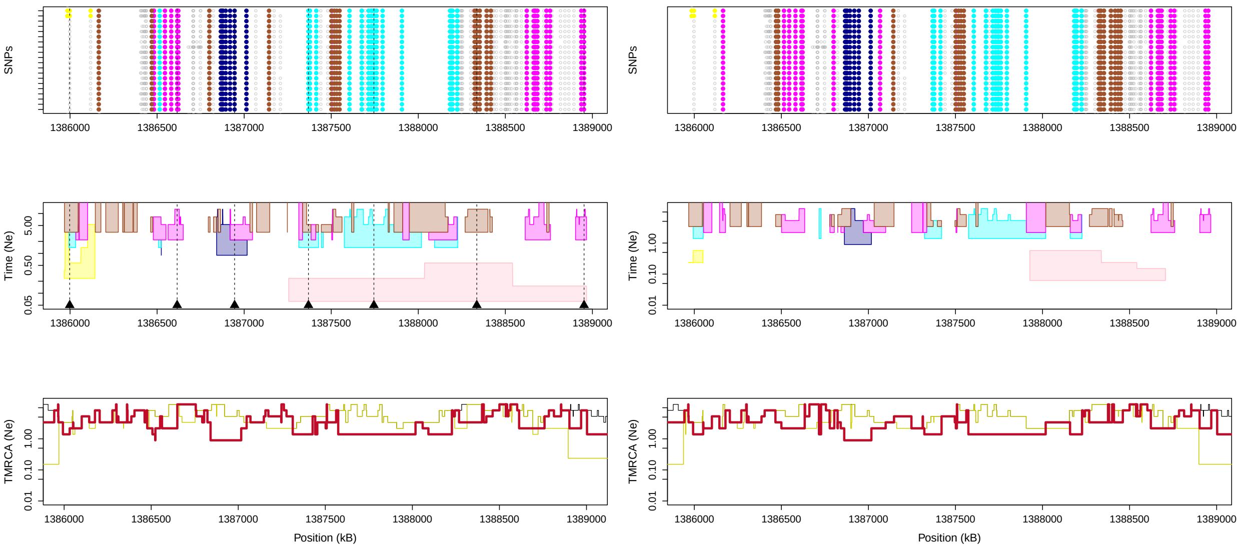

Figure S8: see Figure 6, Main Text for caption; Left panel - Iteration 8250, Right panel - Iteration 9200
